# In silico comparative genomic analysis unravels a new candidate protein arsenal specifically associated with *Fusarium oxysporum* f. sp. *albedinis* pathogenesis

**DOI:** 10.1038/s41598-022-21858-1

**Published:** 2022-11-09

**Authors:** Hafida Ayada, Boutayna Dhioui, Hamid Mazouz, Abdelhay El harrak, Fatima Jaiti, Bouchra Ouhmidou, Mohammed Diouri, Mohieddine Moumni

**Affiliations:** 1 Biotechnology and Bioresources Valorization Laboratory, Biology Department, Faculty of Sciences, Moulay Ismail University of Meknès, Meknès, Morocco; 2 Biodiversity, Environment and Plant Protection Team, Faculty of Sciences and Technologies, Moulay Ismail University of Meknès, Meknès, Morocco; 3 Microbial biotechnology and bioactive molecules laboratory, Faculty of Sciences and Technologies, Sidi Mohammed Ben Abdellah University of Fez, Fez, Morocco

**Keywords:** Genetics, Genomics, Comparative genomics

## Abstract

*Fusarium oxysporum* f. sp *albedinis (*Foa*)* is a devastating fungus of date palms. To unravel the genetic characteristics associated with its pathogenesis, the two available genomes of Foa 133 and Foa 9 were compared with 49 genomes of 29 other pathogenic *formae speciales* belonging to *Fusarium oxysporum* species complex (FOSC). Foa 133 and Foa 9 have genomes of 56.23 Mb and 65.56 Mb with 17460 and 19514 putative coding genes. Of these genes, 30% lack functional annotation with no similarity to characterized proteins. The remaining genes were involved in pathways essential to the fungi's life and their adaptation. Foa secretome analysis revealed that both Foa strains possess an expanded number of secreted effectors (3003 in Foa 133 and 2418 in Foa 9). Those include effectors encoded by Foa unique genes that are involved in Foa penetration (Egh16-like family), host defense mechanisms suppression (lysM family) and pathogen protection (cysteine-rich protein family). The accessory protein SIX6, which induces plant cell death, was also predicted in Foa. Further analysis of secreted CAZymes revealed an arsenal of enzymes involved in plant cell wall degradation. This arsenal includes an exclusively Foa-specific CAZyme (GH5-7). Transcription factors and membrane transporters (MFS) involved in fungicide efflux have been predicted in Foa, in addition to a variety of secondary metabolites. These comprise mycotoxins as well as chrysogin, the latter provides Foa with resistance against adverse environmental conditions. Our results revealed new Foa proteins that could be targeted in future research in order to manage Bayoud disease.

## Introduction

*Fusarium oxysporum* species complex (FOSC) is a ubiquitous group of pathogenic and putatively non-pathogenic soil-borne fungi. It is the most widespread in nature, it colonizes all soil types (cultivated and uncultivated soils) in all continents except Antarctica^1–3^. Among the FOSC, plant pathogenic fungi are known for causing significant ecological and socio-economic damage. In the agricultural field, Fusarium wilt caused by pathogenic fungi belonging to FOSC represents a veritable threat to production and profitability. The *formae speciales* of this species complex attack a multitude of crops, such as legumes (*Fusarium oxysporum* f. sp. *pisi* on peas)^1^, horticultural plants (*Fusarium oxysporum* f. sp. *lycopersici*)^4^, ornamental plants (*Fusarium oxysporum* f. sp. *dianthi* on carnations)^5^ and palm trees (*Fusarium oxysporum* f. sp. a*lbedinis*)^6^. On date palm, *Fusarium oxysporum* f. sp. a*lbedinis* (Foa) causes Fusarium wilt known as "Bayoud". This disease is fairly widespread in the main palm growing areas in North African countries^7^. In Morocco, Fusarium wilt due to Foa has caused the progressive disappearance of high quality and world renown date cultivars, mainly Mejhool^8,9^.

The typical external symptom of the Bayoud disease is hemiplegia character. In the affected palm leaf, the withering begins on one side of the leaf which becomes white; then the withering continues to the other side until the whole leaf dies. In the other hand the important internal symptom is the reddish-brown color of vascular bundles^10^.

Bayoud was first reported in 1870 in Zagora-Morocco. While the first precise description of Foa was done by Malençon in 1934^11^. It is a telluric fungus classified among the imperfect fungi of *Nectriaceae* family*.* The most important means of Foa infection are spores and mycelium. In fact, the infection occurs mainly through the roots and spreads inside the vascular system, leading to wilting and eventually to date palm death^12^. Foa transmission is particularly rapid and spectacular. It can be spread by infected shoots, soil, infected date tissues (especially pieces of infected rachis) and by irrigation water passing through infested fields. Foa can also be passed from one plant to another by contact between diseased and healthy roots^10^.

Given the dangerousness of this pathogen, measured in particular by the enormous damage that results therefrom, it is highly valuable to examine its genetic potential. In this regard, the comparative study of genomes at the structural and functional levels is decisive in such research. The development and/or use of algorithms and bioinformatic tools, dedicated to comparative genomics, has provided a better understanding of the genomes especially those of pathogenic organisms. In *F. graminearum*, comparative genomics has allowed the identification of genes that contribute to phenotypic variation and niche specialization^13^. Another study conducted on FOSC’s *formae speciales* infecting legumes revealed candidate effectors^14^. Regarding Foa, despite the fact that it has been the subject of several investigations for a long time^15–17^, information on its genomics has remained rather quite limited until now. Currently, progress on Foa could be achieved now that its genome is available in databases^18,19^. This will make it possible to meticulously discover phenomena that would otherwise go unnoticed and will thus open up new research avenues to develop novel control techniques that will allow to effectively manage this scourge.

Here, we performed in-depth comparative analysis of the first Foa genomes currently available and 29 other *formae speciales* of FOSC in order to inventory the genetic characteristics involved in Bayoud disease. To the best of our knowledge, this study is the first comparative analysis based on genomic approach conducted on Foa.

## Results

### Phylogeny and genome characteristics of the studied fungal strains

As part of a comparative genomic analysis, the first two Foa genomes publicly available were compared to 49 genomes from 29 other FOSC’s *formae speciales*. The two Foa genomes correspond to two strains originally isolated from infected date palms^18,19^. they were previously sequenced and assembled by the National Institutes of Agronomic Research of Rabat^18^ and Errachidia^19^, Morocco. The Foa genomes as well as the genomes of 29 other *formae speciales* of FOSC were annotated as described in “Material and methods” section.

**Figure 1:**
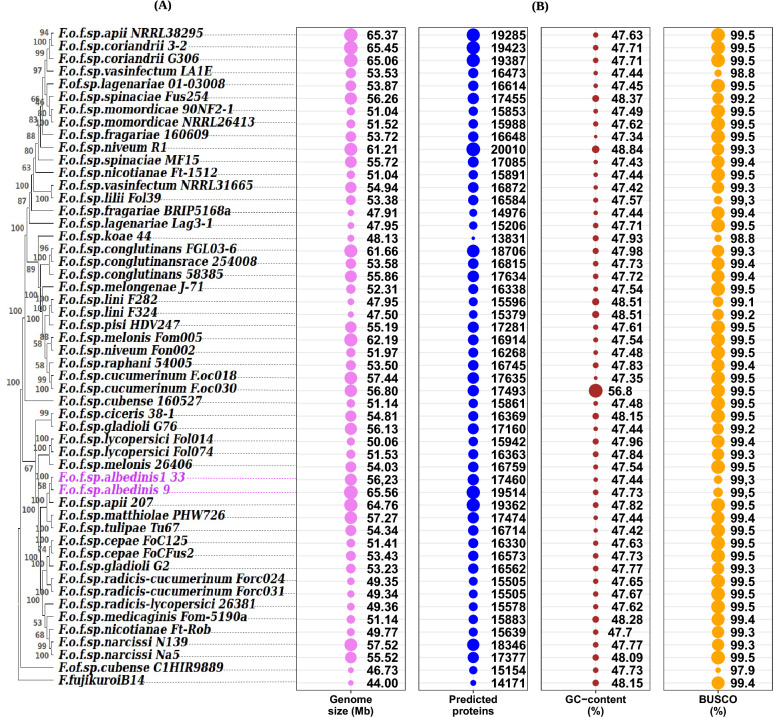
Bubble plots and Dendrogram illustrating the phylogenetic relationships between 49 genomes of the FOSC’s 29 *formae speciales* and two Foa genomes (*formae speciales* of interest). (**A**), phylogenetic tree based on 263 orthologous genes in a single copy. (**B**), four bubble plots showing descriptive statistics for each genome. Bubble sizes have been scaled to the categories and are not comparable between categories. Abbreviations: *F* (*Fusarium*); *o* (*oxysporum*); *f* (*forma*e); sp *(speciales*). The names following the abbreviations represent the names of the *formae speciales*; the abbreviations coming after the names of the *formae speciales* correspond to the strains belonging to these *formae speciales* (F.o.f.sp. *albedinis* 133: *Fusarium osysporum forma specialis albedinis* Foa 133).

Figure 1 showed the phylogeny of the 30 chosen *formae speciales* (A) besides genomic GC content, genome size, genome completeness and proteins putatively encoded by each genome (B). The phylogenetic relationship of the FOSC’s *formae speciales* was conducted based on the concatenated 263 genes that are present as a single copy gene in all genomes in our dataset. The result (Fig. 1A) revealed that the *formae specialis* of this study were not grouped into known clusters and formed distinct branches of their own. Moreover, the phylogenetic tree showed that Foa strains shared the same clade with *matthiolae*, *tulipae* and one strain of *apii,* while Foa strains were relatively distant from the other *formae speciales*.

In comparing statistics of the analyzed genomes, we remarked that some characteristics are quite similar and others are variable. Indeed, Benchmarking Universal Single-Copy Orthologs (BUSCO) analysis showed that more than 99% of the BUSCO genes were present in the most of the analyzed genomes as complete. However, in some strains the genomes covered less than 99% of BUSCO genes (*F.o*.f. sp. *Cubense* C1HIR9889 (BUSCO 97.9%), *F.o*.f. sp. *Koae* isolate 44 (BUSCO 98.8%) and *F.o.* f. sp *vasinfectum* LA1E (BUSCO 98.9%)). The similarity was also observed in genomic GC rate. GC content was consistent, with a small range from 47.34 to 48.84% in all strains except *F.o*.f. sp. *cucumerinum* Foc030 which has more than 50% GC (56.8%). in Foa genomes, GC content varied between 47.44% and 47.73%.

However, the fluctuations of genome sizes within the FOSC’s *formae speciales* were relatively high. The smallest genome was found in *F.o*.f. sp. *Cubense* C1HIR9889 (46.73 Mb) while the largest genome was detected in the *F.o*.f. sp. *albedinis* 9 (65.56 Mb). These fluctuations were also seen between some strains belonging to the same *formae speciales*. In the case of the Foa (*formae speciales* of interest), the genome size varied from 56.23 Mb to 65.56 Mb.

We further examined the gene content in the analyzed strains. The result revealed that there is considerable inter- and intra- *formae speciales* variations. The number of predicted genes ranged from 13831 (*F.o*.f.sp. *koaei* solate 44) to 20010 (*F.o*.f.sp. *niveum* R1) and from 17460 to 19514 genes within the Foa genomes.

Based on the BUSCO gene rate, the analyzed genomes were of a high enough quality for downstream analysis. Moreover, according the phylogenetic relationship and the noted variation in genome size and gene content, the 30 analyzed *formae speciales* seem to be relatively diverse.

### Homologous and specific genes analysis:

Given the inter- and intra- *formae speciales* genetic diversity signs within the FOSC’s *formae speciales*, we examined the extent of genome diversification. For this purpose, we performed a pan-genome analysis. We were interested in 4 conceptual groups of genes:

- The pan-genome: inventory of genes presents within the analyzed strains
- The core-genome: set of homologous genes that are present in all genomes of the analyzed dataset
- The accessory-genome: set of genes present within one or part of the analyzed genomes.
- The unique or specific-genome: set of genes found in only one strain and absent in others.

We first inventoried homologous (orthologous and paralogous) and specific genes using a BLASTp-based pipeline (see methods). Proteins whose sequences and functions were similar have been grouped in the same family.

**Figure 2:**
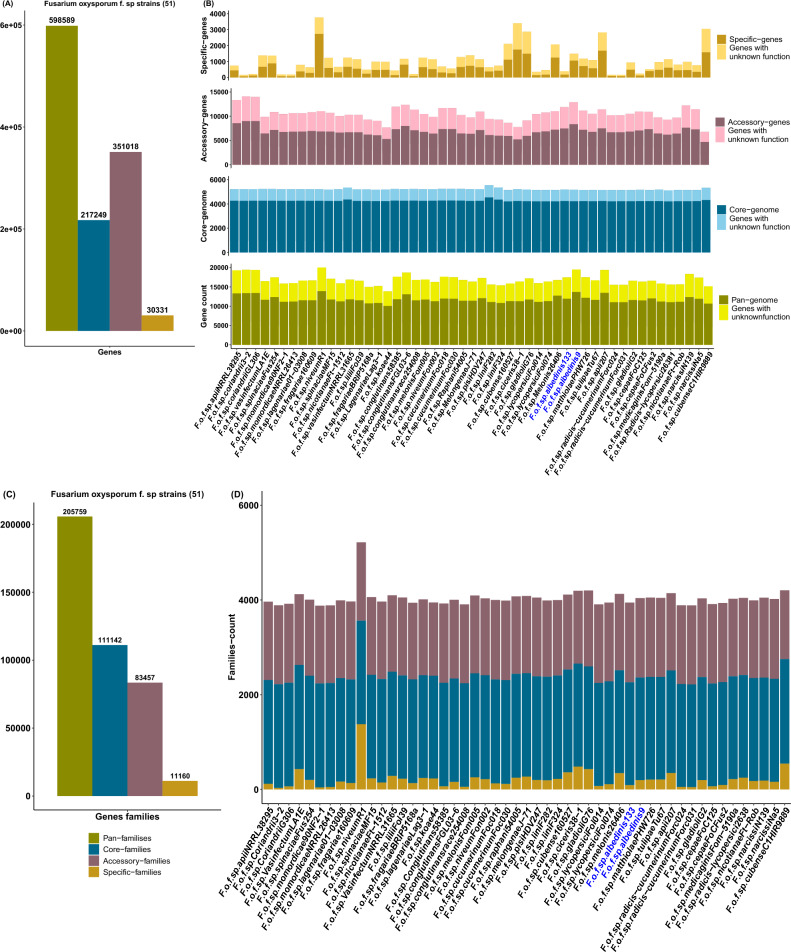
The pan-genome of the FOSC’s *formae speciales* including Foa. (**A**, **C**), histograms representing the total number of genes and families distributed over the pan-genome, core-genome, accessory-genome and unique-genome for the analyzed strains. (**B**), Stacked histograms of the gene numbers of the pan-genome, core-genome, accessory-genome and unique-genome for each strain. (**D**), Stacked histograms of the number of core-families, accessory-families and unique-families for each strain. Abbreviations: *F* (*Fusarium*); *o* (*oxysporum*); *f* (*forma*e); sp *(speciales*). The names following the abbreviations represent the names of the *formae speciales*; the abbreviations coming after the names of the *formae speciales* correspond to the strains belonging to these *formae speciales* (F.o.f.sp. *albedinis* 133: *Fusarium osysporum forma specialis albedinis* Foa 133).

The FOSC’s *formae speciales* pan-genome comprised a total of 598589 genes (Fig. 2A) consisting of 217249 genes in the core-genome and 351018 genes in the accessory-genome. With regard to the unique-genome, 30331 genes were without homologs in the other genomes, indicating the existence of specific genes among the FOSC’s *formae speciales*. The lowest numbers of unique genes belonged to the *F.o*.f.sp. *Coriandrii*3–2, with 155 genes. Strain *F.o*.f.sp. *niveum*R1 contained the highest numbers of specific genes as well (Fig. 2B and Supplementary Data 2: Table 1). Within Foa *formae speciales*, represented here by strain 133 and 9, the pan-genome consisted of 17460 and 19514 genes respectively (Fig. 2B and Supplementary Data 2: Table 1). In both Foa strains, the core-genome was comprised of an average of 5168 genes. However, the large portion of Foa pan-genome was predicted as accessory-genes.

**Table 1:** KEGG pathways enriched in unique genes of the 133 and 9 Foa strains.

Categories	*F. oxysporum* f. sp. *albedinis* 133		*F. oxysporum* f. sp*. albedinis* 9	
	Gene numbers	Percentage (%)	Gene numbers	Percentage (%)
**Metabolism**	**41**		**5**	
Carbohydrate metabolism	9	21.9	2	40
Lipid metabolism	13	31.7	0	0
Amino acid metabolism	9	21.9	1	20
Glycan biosynthesis and metabolism	1	2.4	0	0
Metabolism of cofactors and vitamins	1	2.4	0	0
Metabolism of terpenoids and polyketides	0	0	1	20
Biosynthesis of other secondary metabolites	2	4.9	1	20
Xenobiotics biodegradation and metabolism	4	9.8	0	0
Enzyme families	2	4.9	0	0
**Genetic information Processing**	**12**		**2**	
Transcription	1	8.3	0	0
Translation	3	25	0	0
Folding, sorting and degradation	5	41.7	1	50
Replication and repair	3	25	1	50
**Environmental information Processing**	**8**		**5**	
Membrane transport	7	87.5	3	60
Signal transduction	1	12.5	2	40
**Cellular processes**	2		**1**	
Transport and catabolism	2	100	1	100
**Unclassified**	**5**		**1**	
Unclassified	**5**	100	**1**	100

Values in bold are significant values, they indicate the total number of unique genes involved in each KEGG pathway reported.

They represent 68% (11884 genes) of Foa 133’s genome and cover 66% (12873 genes) of Foa 9’s genome. As for the unique genome, Foa 133 contained a low number of unique genes (396 genes) compared to Foa 9 whose genome included 1,483 specific genes.

To assign biological functions to the three genomic fractions (core, accessory, and unique), Interproscan 5 was used. The resulting annotations revealed that the pan-genome of the analyzed strains was grouped into 205759 gene families (Fig. 2C). Of those families, 54% were found in all genomes (111142 families). They contained proteins encoded by the core genes. Despite the high gene numbers in the accessory genome, the total of predicted gene families was lower than that of the core families. With regard to the unique genome, 11160 specific gene families were found. An examination of gene repertoire of Foa strains showed that about 30% of pan-genome lacked annotations. In both strains 82% of the core genes were identified. They were classified into an average of 2161 families (Fig. 2D and Supplementary Data 2: Table 2) and were primarily associated with transporters (200–207 genes) and fungal-specific transcription factors (127 genes). For the accessory-genome 35–37% of the genes lacked InterProscan assignations in both Foa strains. In addition to transporters and transcription factors, the annotated proportion of Foa accessory genes were assigned to P450 cytochromes (305 and 312 genes in strains 133 and 9 respectively) (Fig. 2D and Supplementary Data 2: Table 3). Though 52% of Foa 133-specific genes and 28% of Foa 9-specific genes were not assigned to the Interproscan annotations, the remaining specific genes in both Foa strains fall into different families (Fig. 2D and Supplementary Data 2: Table 4). They primarily encoded endonucleases (41 and 273 genes respectively) and the hAT activator family (21 and 334 genes respectively).

Through these results, an abundance of accessory genes was revealed in Foa genomes. These genes provide information about horizontal transfers dynamics because they are often associated with potentially transferable elements. Moreover, the presence of specific genes in Foa genomes suggested that these strains harbored a high level of genomic diversity and uniqueness of each strain, showing their ability to acquire specific characteristics.

### TEs in the analyzed genomes

Another conducted analysis was the search for transposable elements (TEs) in the studied strains. These are DNA fragments that can move and multiply in genomes. As such, TEs can have functional and structural impacts on genomes^34^. They are also considered to be the main driver of genome inflation^35^.

**Figure 3:**
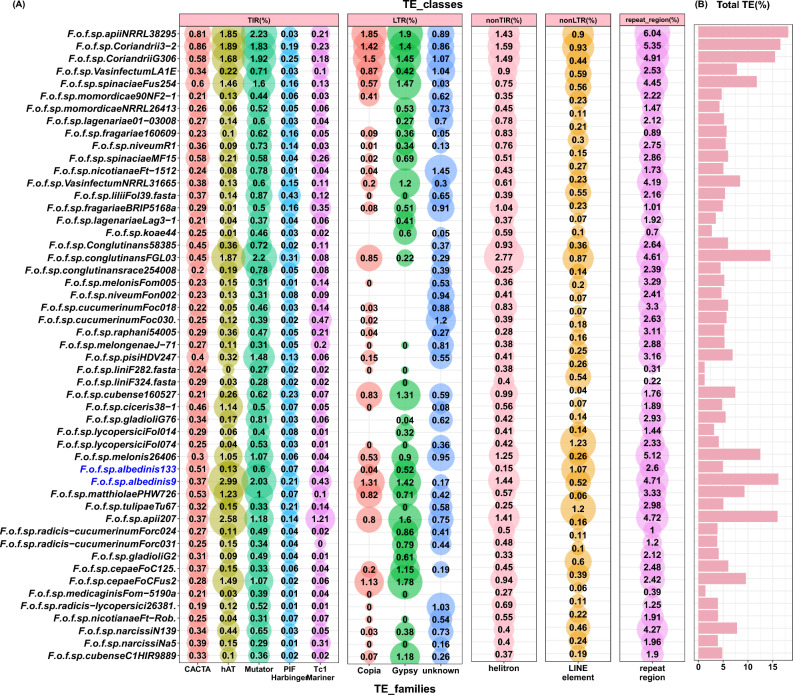
Distribution and coverage (%) of predicted TE in the analyzed *formae speciales*. (**A**): bubble plot showing the predicted TE distributed on five classes: Long Terminal Repeated retrotransposons (LTR); Terminal Inverted Repeats (TIR); non-TIR; non-LTR and other repeated regions. The bubble size is proportional to the coverage of each TE family (% indicated inside bubbles). (**B**): bar plot showing the total TE coverage per genome. Abbreviations: *F* (*Fusarium*); *o* (*oxysporum*); *f* (*forma*e); sp *(speciales*). The names following the abbreviations represent the names of the *formae speciales*; the abbreviations coming after the names of the *formae speciales* correspond to the strains belonging to these *formae speciales* (F.o.f.sp. *albedinis* 133: *Fusarium osysporum forma specialis albedinis* Foa 133).

The search using EDTA software identified 5 putative TE classes: long terminal repeated retrotransposons (LTR); terminal inverted repeats (TIR); non-TIR; non-LTR and other repeated regions (Fig. 3). Within the Foa *formae speciales*, the distribution of TE categories varied between strains. The total TE coverage in Foa 9 (16.5%) was higher compared to Foa 133 (4.92%). In Foa 133, the LTRs covered 0.56% of the genome; they belonged mainly to the Gypsy family (0.51%). As for the TIRs, they accounted for 1.35% of the genome and the majority were Mutator (0.6). On the other hand, LTRs and TIRs were most abundant in Foa 9 and occupied respectively 2.9% and 6.03% of the genome. Note that the maximum TIRs in this strain, were mainly hAT. In addition, we found that the genomes of the Foa *formae speciales* contained a significant fraction of other repeated regions (2.6–4.71%), helitron (0.15–1.44%) and LINE-element (0.26–1.07%). This examination revealed that the Foa genomes were enriched in numerous TEs families.

### KEGG pathways assigned to proteins of the analyzed strains:

**Figure 4:**
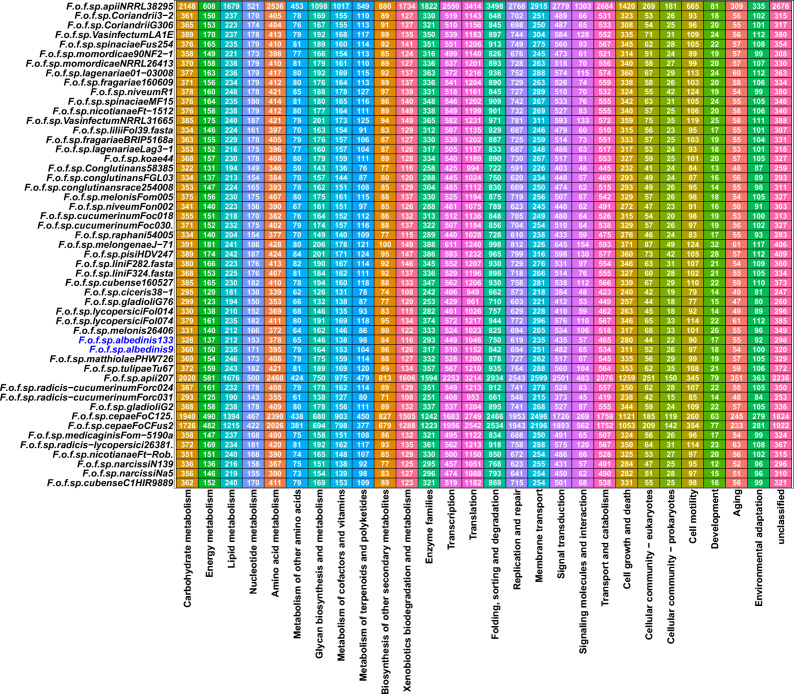
Predicted KEGG categories in Foa and other FOSC’s *formea speciales*. KEGG annotations are provided using the profile database that is integrated into the Kofamscan software. The numbers represent the genes number involved in each pathway for each strain. Abbreviations: *F* (*Fusarium*); *o* (*oxysporum*); *f* (*forma*e); sp *(speciales*). The names following the abbreviations represent the names of the *formae speciales*; the abbreviations coming after the names of the *formae speciales* correspond to the strains belonging to these *formae speciales* (F.o.f.sp. *albedinis* 133: *Fusarium osysporum forma specialis albedinis* Foa 133).

To further explore the genetic potential of the Foa *formae speciales*, KEGG database was used to highlight the pathways in which Foa genes are involved compared to other *formae speciales*. As shown in Fig. 4, 27 pathways were represented for all strains. Within these pathways, the genetic information processing category (represented by the translation, folding/sorting/degradation, and replication/repair pathways) was the most enriched in gene.

Within Foa, 7102 genes in Foa 133 and 7848 genes in Foa 9 were distributed over the 27 predicted pathways. The examination of this distribution revealed that the maximum number of genes were involved in the translation process (1046 genes in Foa 133 and 1152 genes in Foa 9); followed by the folding, sorting and degradation pathway, which contain 750 genes in Foa 133 and 842 genes in Foa 9. It should also be noted that a large number of genes (656 genes on average) in this *formae speciales* were attributed to the replication/repair pathway. These three pathways maintain the vital functions of the fungal cell and were therefore enriched mainly in core genes and accessory genes that can further enhance these functions. Moreover, Foa genomes included genes involved in cell motility, allowing Foa to actively circulate in the soil as mentioned in other fungi^36^. We were then interested in the pathways enriched in unique genes of this *formae speciales* (Table 1). We found that a reduced number of unique genes were involved in 17 of the 27 predicted pathways. In Foa 9, 67 genes were assigned to the KEGG annotations. Of those genes, 59.7% (40 genes) were included in the metabolism category. Within this category, the maximum number of genes (13 genes) was assigned to lipid metabolism, followed by carbohydrate metabolism (9 genes), amino acid metabolism (9 genes) and xenobiotic metabolism/degradation (4 genes). However, in Foa 133, a total of 14 unique genes were assigned to the KEGG pathways, where the membrane transport pathway was the most enriched in unique genes (3 genes), while the xenobiotic degradation pathway did not include any gene in this strain. Collectively, these pathway analysis results illustrated that most of the Foa genes are involved in the pathways essential to the fungi's life and their adaptation to environmental conditions.

### Candidate secreted effectors in Foa:

In pathogenic fungi, secreted proteins, particularly effectors, are essential for successful host infection^37^. These proteins can disable plant defenses and subvert cell processes to satisfy the needs of pathogens^38^.

In this context, the secreted effectome of all the analyzed strains was highlighted. Through the combined use of SignalP v5.0, SecretomeP v1.0 and EffectorP v3.0 to predict putative secreted effectors, mining the Foa genomic data resulted in a catalogue of 3003 effectors in Foa 133 and 2418 effectors in Foa 9 (Supplementary Data 3: Table 1). This constitutes the putative effector repertoire of Foa strains. Of those effectors, more than 800 proteins were encoded by the core genome in both Foa strains; whereas the accessory genes encoded more than 60% of this effector repertoire in both Foa strains (2096 effectors in Foa 133 and 1542 effectors in Foa 9). As for the unique genome, a low number of effectors were encoded by this genomic fraction, these effectors represented 0,03% of the secreted effectome (94 effectors in Foa 133 and 74 effectors in Foa 9).

In the three genomic fractions (core, accessory and unique) the predicted effectors were classified into two categories: cytoplasmic effectors that act inside the plant cells and apoplastic effectors that manipulate the host apoplasm. For all strains, more than 80% of the secreted effectors were cytoplasmic. In Foa, this category occupied 85% and 83% of predicted effectors in Foa 133 and 9 respectively. Within the apoplastic and cytoplasmic categories, effectors lacking Interproscan annotations ranged between 41 and 58%. The apoplastic and cytoplasmic effectors with known function were organized into 803 families in Foa 133 and 779 families in Foa 9 (Supplementary Data 3: Table 2). they mainly belonged to DDE superfamily endonuclease (41 effectors in Foa 133 and 106 effectors in Foa 9).

**Figure 5:**
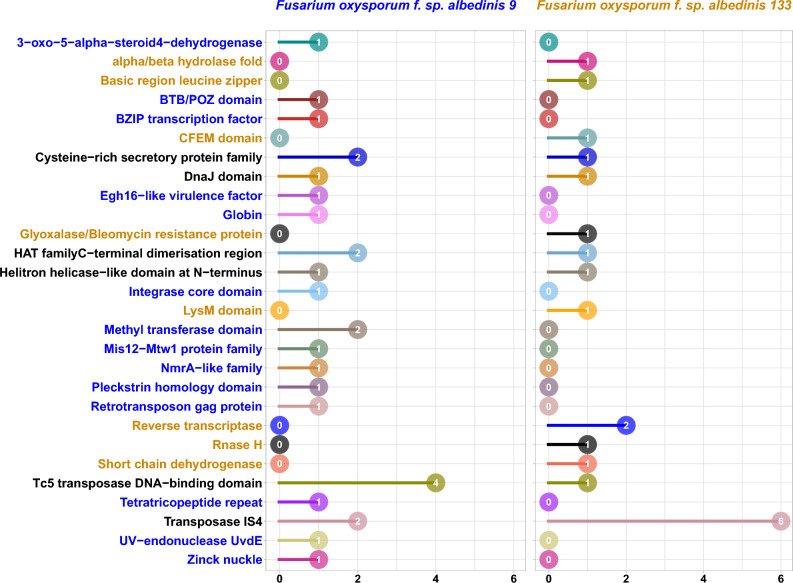
Predicted secreted effectors in the Foa *formae speciales* unique genome*.* The colors represent the predicted effector families and the numbers indicate the count of effectors in each family for each Foa strain.

In Fig. 5, 28 protein families were predicted as families comprised of effectors encoding by Foa specific genes. This constitute the Foa unique secreted effectome. In comparing Foa strains, 14 effector families (families written in blue) in Foa 9 were not presents in Foa 133 such as Egh16-like virulence factor. On the other hand, Foa 133 was distinguished from Foa 9 by secreting effectors belonging to 8 families (families written in orange) absent in Foa 9 such as LysM family.

Among the 28 putative effector families found in Foa strains, 3 families were found only in Foa 9 in comparison to the other analyzed *forma speciales* (Supplementary Data 3: Fig. 1). Foa 9 was characterized by the secretion of effectors belonging to 3-oxo-5-alpha-steroid4-dehydrogenase, Integrase core domain and Globin families.

Taken together, secreted protein repertoire of Foa strains harbored numerous putative proteins acting as effectors, 3 of these were exclusively specific to Foa 9.

### Carbohydrate Active enZymes (CAZymes) repertoire of the analyzed strains:

**Figure 6:**
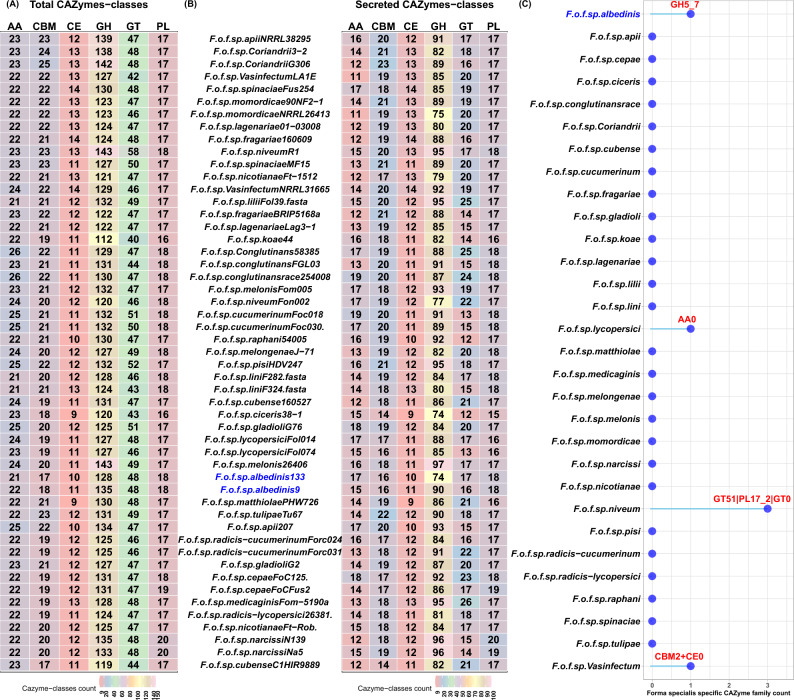
Predicted CAZymes in the analyzed strains. (**A**): heat map showing the total number of CAZymes distributed on six categories: auxiliary activities (AA), carbohydrate-binding molecules (CBM), carbohydrate esterases (CE), glycoside hydrolases (GH), glycosyltransferases (GT) and polysaccharide lyases (PL). (**B**): heat map showing the number of secreted CAZymes for each strain. (**C**): lollipop plot representing CAZyme families specific to each *formae speciales*. Abbreviations: *F (Fusarium); o (oxysporum); f (forma); sp (specialis).* The names following the abbreviations represent the names of the *formae speciales*; the abbreviations coming after the names of the *formae speciales* correspond to the strains belonging to these *formae speciales* (F.o.f.sp. *albedinis* 133: *Fusarium osysporum forma specialis albedinis* Foa 133).

All plant pathogenic fungi are known for producing carbohydrate-active enzymes (CAZymes). These enzymes are involved in the assembly, modification or deconstruction of carbohydrates^39^. They are considered to play a key role in the degradation of plant cell wall, which is the front line of plant defense^40^. Given the importance of such enzymes, run-dbcan software was used to predict the CAZyme content in the Foa genomes in comparison with the other *formae speciales*. As shown in Fig. 6A, the analyzed genomes encoded an arsenal of CAZymes organized in six classes: auxiliary activities (AA), carbohydrate-binding molecules (CBM), carbohydrate esterases (CE), glycoside hydrolases (GH), glycosyltransferases (GT) and polysaccharide lyases (PL). In Foa*,* 1.4% (242 genes in Foa 133) and 1.3% (252 genes in Foa 9) of the genome encoded these enzymes. The highest number of predicted CAZymes (53%) was related to GH followed by GT, AA, CE, PL and CBM. Interestingly, more than 50% of the majority of predicted CAZymes were secreted (Fig. 6B). In both Foa strains, 100% of CEs and PLs were secreted. In contrast, CAZymes belonging to GT class were mainly intracellular (35% of the total GT predicted were secreted).

To further analyze carbohydrate utilization ability of Foa, we examined the different families of secreted CAZymes (Supplementary Data 4: Table 1). It was found that the most abundant identified GH were related to the GH3 and GH28 families, including the largest numbers of the secreted GH. The second most frequent CAZyme families contained in Foa genomes was GT1, GT2 and GT4, which comprised the majority of GT CAZymes. In AA class, all CAZymes were organized in 4 families (AA1, AA3, AA5, AA6 and AA9), while the majority of CBMs were assigned to CBM1 and CBM32. As for PLs, our result revealed that they belonged mainly to PL1 family. Interestingly, our results showed that Foa was distinguished from all other analyzed *formae speciales* by the secretion of the glycoside hydrolase GH5-7, as shown in Fig. 6C.

The examination of CAZyme repertoire showed the enrichment of CAZyme families in Foa genomes with the exclusive secretion of glycozyle hydrolase (GH5_7). This enzyme is important to decompose hemicellulosic materials.

### Repertoire of xylem secreted proteins (SIX) detected in the analyzed strains:

Within the FOSC, some pathogens secrete small effectors in the xylem sap of the host plant^41,42^. Currently, 15 classes of these proteins have been identified and are called Secreted In Xylem (SIX). These proteins contribute to the virulence of these microorganisms.

**Figure 7:**
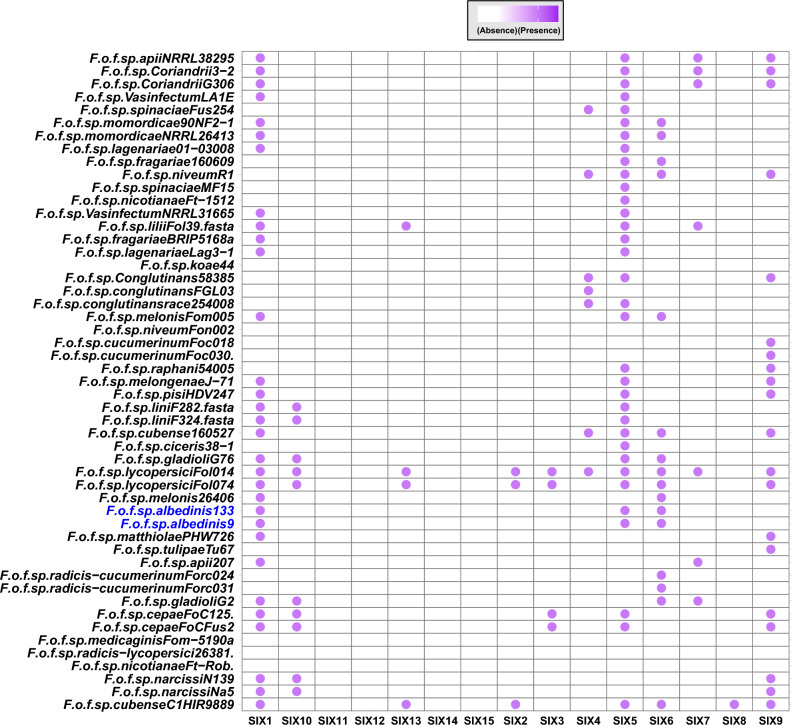
Presence/absence map of SIX protein distribution in the protein repertoire of Foa and other analyzed *formae speciales*. The bubbles indicate the presence of SIX proteins and the empty boxes represent their absence in each strain. Abbreviations: *F (Fusarium); o (oxysporum); f (forma); sp (specialis)*. The names following the abbreviations represent the names of the *formae speciales*; the abbreviations coming after the names of the *formae speciales* correspond to the strains belonging to these *formae speciales* (F.o.f.sp. *albedinis* 133: *Fusarium osysporum forma specialis albedinis* Foa 133).

The importance of these effectors led us to investigate whether Foa genomes encode these proteins. It was revealed that only 3 accessory genes encoded 3 classes of SIX proteins: SIX1, SIX5, and SIX6. However, the core and unique genomic fractions were not involved in this secretion pattern, as some *formae speciales* lacked these effectors and there was no *formea speciales* -specific class (Fig. 7).

### Secondary metabolism in analyzed strains:

In fungi, secondary metabolites play ecological, symbiotic and pathogenic roles^43^. These small molecules are encoded by biosynthetic genes (BGCs) that are grouped into clusters in the genome^44^. To examine the presence of putative secondary metabolites within Foa, the algorithms implemented in antiSmashe software were applied to the fungal genomes of the strains targeted by our analysis.

**Figure 8:**
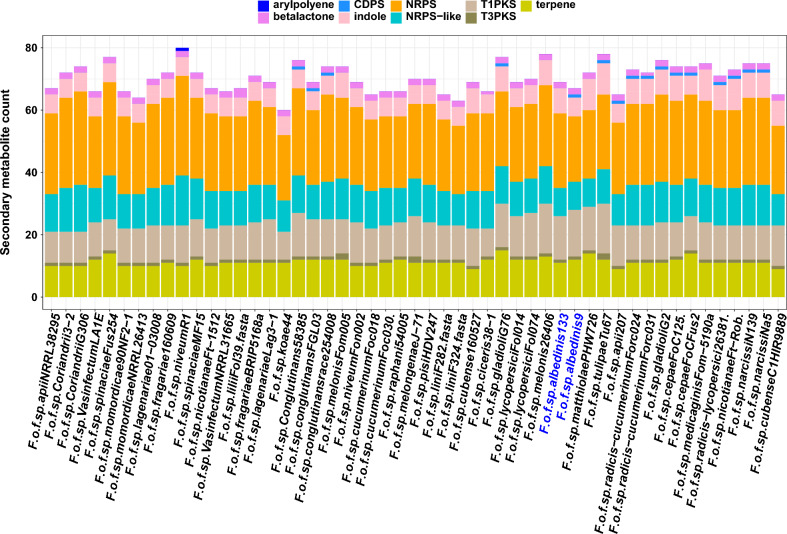
Predicted secondary metabolite classes in the analyzed *formae speciales*: sckqted plot showing the predicted secondary metabolite genes for each strain; these genes were involved in the synthesis of nine secondary metabolite classes: arypolyene, betalactone, indole, terpene, CDPS (CycloDiPeptides Synthase), NRPS (Non-Ribosomal Peptide Synthase), NRPS-like, T1PKS (Type I Polyketide Synthase) and T3PKS (Type III Polyketide Synthase). Abbreviations: *F (Fusarium); o (oxysporum); f (forma); sp (specialis)*. The names following the abbreviations represent the names of the *formae speciales*; the abbreviations coming after the names of the *formae speciales* correspond to the strains belonging to these *formae speciales* (F.o.f.sp. *albedinis* 133: *Fusarium osysporum forma specialis albedinis* Foa 133).

Therefore, a total of 136 BGCs were predicted in the Foa *formae speciales*, we found 69 and 67 BGCs in Foa 133 and 9 respectively. These genes were involved in the biosynthesis of 9 chemical classes of secondary metabolites (Fig. 8), the majority (71%) of these metabolites belonged mainly to 3 classes: Type I Polyketide synthase (T1PKS, an average of 14 BGCs), terpenes (an average of 12 BGCs) and Non-ribosomal peptide synthetase (NRPS, an average of 22 BGCs).

**Figure 9:**
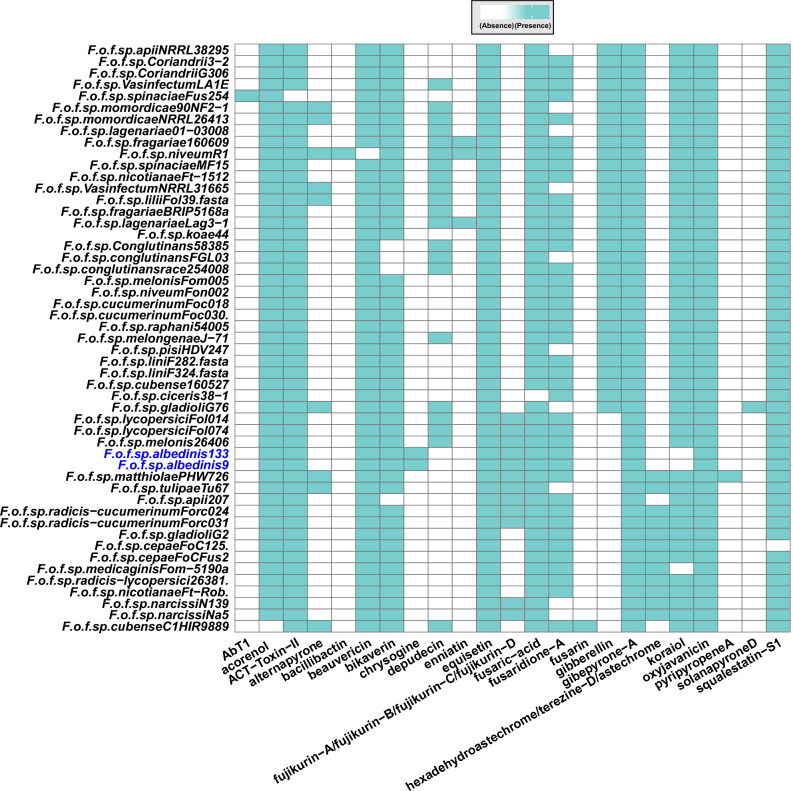
Predicted secondary metabolite families in the analyzed *formae speciales*. presence/absence map of secondary metabolite families detected in the genomes of the Foa and the other *formae speciales*. Blue boxes indicate the presence of secondary metabolite families and empty boxes indicate their absence in each strain. Abbreviations: *F (Fusarium); o (oxysporum); f (forma); sp (specialis)*. The names following the abbreviations represent the names of the *formae speciales*; the abbreviations coming after the names of the *formae speciales* correspond to the strains belonging to these *formae speciales* (F.o.f.sp. albedinis 133: *Fusarium* osysporum forma specialis albedinis Foa 133).

The predicted BCGs were then associated with characterized and verified clusters from the MIBiG database. Based on this, 12 families were identified within the Foa *formae speciales* (Fig. 9). Of these, 3 families (oxyjaneancine, gibepyrone A and equisetin) were produced by all *formae speciales*. On the other hand, the secondary metabolites belonging to the chrysogin family were specific to the Foa strains and were mainly encoded by unique genes. We also noted in Foa, the biosynthesis of mycotoxins which include ACT-Toxin-II and beauvercin as well as the biosynthesis of metabolites having anti-fungal activity (fujikurin A–D).

According to our in silico analysis, some metabolites were produced by all of the *formae speciales*; however metabolites belonging to chrysogin family were specifically synthetized by Foa strains.

## Discussion

*Fusarium oxysporum* f. sp *albedinis* (Foa) is the causal agent of the most destructive disease of date palm in the oasis’s regions of Morocco and other North African countries^45^. Until now, no effective treatment has been established against this disease called Bayoud^45^. For the first time in its history, we approach the study of this disease based on genomic analysis. Indeed, genomes sequencing of Foa strains provides important data to deeply study this disease and to design new control strategies.

To provide a comprehensive overview and uncover the genetic traits of this pathogenic fungus, a comparative genomic analysis of the two Foa genomes and the genomes of 29 other FOSC's *formea speciales* was conducted in this work. In comparing 263 core genes, we showed that Foa was phylogenetically located in the same clade including *matthiolae, tulipae* and *apii.* These *formae speciales* are known to attack temperate climate plants (Brassicaceae, Liliaceae and Apiaceae). Recently, a phylogeny based on the Foa mitochondrial genome was carried out and has highlighted the Foa clustering in the same clade as *lycopercisi formae speciales*^46^. However, this is relative as it was based only on the comparison of the mitochondrial DNA and not on the analysis of the genomic DNA which provides exhaustive information on the pathogen. In-depth structural and functional annotations were performed to extract accurate information about Foa. It was found that the genomes of this *formae speciales* harbor numerous encoding genes (17460–19514 genes) with around 30% that remain uncharacterized (unknown function). The gene families that control vital cellular functions were mainly derived from the core genome where a large number of genes encoded MFS (Major Facilitator Superfamily). These are known as omnipresent proteins in all vital functions and constitute the largest family of secondary transporters^47,48^. They are involved in essential cellular functions, such as nutrient uptake and metabolite extrusion^48,49^. In addition, a recent study^50^ showed that in *F.virguliforme,* MFS conferred reduced sensitivity to fluopyram (fungicide). Other such abundant core families in Foa include proteins having the fungal-specific transcription factor domain. Predictions suggest that factors with this domain are involved in carbohydrate metabolism, amino acid metabolism, gluconeogenesis, respiration and fatty acid catabolism (TRANSFAC, PFAM). However, the role of these proteins remains unknown, as these predictions have not all been experimentally confirmed. In some fungi, these factors play an important role in the coordination of multiple physiological processes, such as regulation of sensitivity to fludioxonil (fungicide), and of pathogenesis^51^.

As to the remaining genome of the Foa *formae speciales*, it was mainly composed of accessory genes (70% of the total predicted genes) this fraction is enriched in proteins having the fungus-specific transcription factor domain and in proteins belonging to the Cytochrome P450 family. The latter are used by fungi as rapid adaptation strategies in several ecological niches^52–54^, they are involved in the degradation processes of the plant cell wall^55^. Furthermore, the richness of accessory genes can provide information on the horizontal transfer dynamics as they are often associated with potentially transferable elements such as transposons. Indeed, TEs are important features of fungal genomes and play a key role in genome structure and plasticity^56^. In addition to the fot1 family mentioned in previous studies^57^, the analyses carried out in the present work revealed that Foa genomes harbor numerous transposable elements categories that cover a significant genomic portion. The high content of TEs can explain the variation in total genome size of the two Foa strains. This size decreases considerably when TEs are excluded. It varies between 53.43 Mb (in Foa 133) and 54.76 Mb (in Foa 9) instead of 56.23 Mb and 65.56 Mb respectively.

Our results are consistent with those of Li-Jun Ma and their team^58^ who showed that the genomes of other *Fusarium* such as *F. graminearum*, *F. verticillioides* and *F. oxysporum* f. sp. *lycopersici* are also enriched in transposable elements and pathogenicity-related genes, indicating horizontal acquisition.

Another finding is that the core and accessory genomic fractions harbor a gene set that enables fungal cell motility, indicating that Foa could actively move towards the plant roots which is the starting point of the infection. The proteins encoded by these genes could therefore be valuable targets for new control methods.

Similar to all the analyzed *formae speciales*, Foa can be distinguished from the others by a genomic fraction that is specific to it (the unique genes). These genes regulate numerous processes. In Foa 9, 4 unique genes are involved in the degradation of xenobiotics (chemicals such as fungicides, pollutants, etc.). In comparison with Foa 133, the presence of these unique genes, in addition to the core and accessory genes involved in this pathway, could confer and increase the resistance of this strain to fungicides and environmental stress due to contaminants accumulation. This is consistent with other fungi, where the xenobiotics degradation enabled them to resist dichlorvos^59^.

Furthermore, our results revealed that a subset of the Foa unique gene repertoire encodes secreted effectors. The role of effectors in the virulence of pathogenic fungi has been investigated extensively. Here we have shown that the protein repertoire of the analyzed Foa strains harbors key effectors of infection. These include proteins having a LysM domain, given that several LysM effectors have already been identified as virulence factors in plant pathogenic fungi^60^. These proteins inhibit the chitin-induced immune response, which protects the fungal cell walls from host chitinases^61^. Another type of effectors secreted by Foa are belonging to the cystin-rich proteins family. These are apoplastic effectors in which the cysteine residues form disulfide bonds, thus increasing the stability of these effectors in the protease-rich apoplastic space^62^. In this context, a recent study^63^ revealed that these effectors play a key role in the interaction between *Verticillium dahliae* and its host plant by suppressing immunity after infection. Foa also secretes the virulence factor Egh16-like (predicted in Foa 9) which enables it to penetrate the plant cell. This type of factors is known to be involved in appressorium creation in plant pathogenic fungi^64^. In *Magnaporthe oryzae* (rice borer fungus), deletion of Egh16 orthologous genes altered fungal ability to penetrate host cuticles ^65^. The effector repertoire mining in Foa revealed effectors exclusively specific to Foa 9. this strain secretes protein predicted as 3 − oxo − 5 − alpha − steroid4 − dehydrogenase. In rice blast fungus, gene encoding this effector were specifically expressed during infection stages^66^. the effector referred to as Globin was putatively identified in Foa 9. this protein was reported to be involved in low oxygen adaptation of other fungi^67^. thus, Foa 9 could grow in microaerobic environments with low O2 levels.

A further class of effector proteins encoded by the Foa genomes are SIX1, SIX5 and SIX6. They are regulated by accessory genes, whose presence could be the result of horizontal gene transfer. These proteins further enhance Foa pathogenesis. Indeed, the presence of SIX1 has been reported to be a prerequisite for achieving complete virulence of the c*onglutinans* (Focon) and *lycopersici* (Fol) pathogens on cabbage and tomato, respectively^68^. Further studies on *Fol* showed that infection using mutants lacking SIX5 gene showed a considerable reduction in disease symptoms; and reintroduction of the gene restored pathogenesis in 75% of mutants^69^. As for SIX6, this protein was shown to play a role in pathogenic fungi virulence by inhibiting a hypersensitivity response (HR)^70^. In *Nicotiana benthamiana* leaf cells, the SIX6 protein induced cell death by Avr2-I-2 interaction^71^. This arsenal of predicted effectors in Foa could serve as target proteins to setting up an effective treatment against Bayoud.

The genetic potential of Foa is not only restricted to the secretion of the above-mentioned effectors. This pathogen produces other proteins that are involved in the infection process. Our results showed that Foa strains carry a high number of genes encoding CAZymes associated with the degradation of the plant cell wall (the first physical barrier in front of Foa). Our results have further shown that this CAZyme arsenal is in part secreted. The secretome prediction revealed that the predicted CAZymes are partly secreted in Foa, thus suggesting the cellular location of the remaining CAZymes. This is reported in other fungi such as *Flammulina elastica* where the third of predicted GH genes were intracellular^72^.

As in all the examined FOSC’s *formae speciales*, the Foa secreted CAZyme fraction comprises enzymes having known activities, which are organized in numerous families clearly associated with the degradation of key plant cell wall components. Another important finding is that the Foa is distinguished from all other analyzed *formae speciales* by the secretion of an endo-b-1,4-mannanase (GH5-7). This enzyme can efficiently cleave higher molecular weight mannans (plant wall hemicellulose) consisting of more than six mannose monomers^73^. In addition, the combination of mannanases and mannosidases can further increase the mannan catalysis by 83%^74^. A previous studies revealed that date palm is almost entirely composed (92%) of linear mannan (high molecular weight molecule)^75^, giving it a hardness, especially in seeds, to protect it from mechanical damage^76,77^. Therefore, the exclusive occurrence of the gene encoding mannanase (GH5-7) in Foa genomes could explain its specificity towards date palm.

In phytopathogenic fungi, other compounds can further improve infection processes. These fungi are known to produce a wide variety of secondary metabolites that often confer ecological advantages and enhance their infectiveness. In this regard, our results showed the presence of numerous gene clusters involved in the biosynthesis of these compounds in Foa. The predicted metabolites are often mycotoxins such as ACT-Toxin-II and Beauvericin. ACTs are host-specific toxins originally found in *Alternaria* pathotypes infecting mandarins^78^. The ACT toxin induces plasma membrane dysfunction resulting in electrolyte leakage and rapid cell death^79^. As for beauvericin (BEA), it is a mycotoxin produced by *Fusarium* species, has cytotoxic activity and is able to increase oxidative stress to induce cell apoptosis^80^. The Foa secondary metabolite pool also comprises antibiotics and compounds that protect it from toxic products (gibepyrone A)^81^, as well as other metabolites that exhibit anti-fungal activity (fujikurin A–D).

Our results revealed that Foa, in contrast to all other analyzed genomes, can synthesize chrysogin. This pigment is known to protect microorganisms against abiotic aggressors such as UV light^82^. Therefore, Foa seems to be more able to withstand severe environmental conditions compared to the other analyzed *formae speciales*.

## Conclusion

The two Foa genomes analyzed in this work are the first genomes sequenced and reported in the literature. The availability of these sequences has provided us with an extensive overview of the Foa genomics and its pathogenesis and adaptiveness. The in-depth analysis of these two genomes revealed the genetic characteristics of Foa. We showed that Foa secretes a wide range of effector proteins. These proteins comprise the virulence factor Egh16-like and other effectors (LysM, and SIX proteins) that enhance Foa's ability to infect and destroy its host. Our analyses also revealed that the Foa genome encodes an arsenal of secreted enzymes involved in plant cell wall degradation. This arsenal includes an enzyme (GH5-7) which is exclusively specific to Foa. This enzyme could define the specificity of Foa to its host. In addition to these enzymes, predicted effectors and putative secondary metabolites reflect the pathogenic nature of Foa. The discovery of all these proteins has shed light on mechanisms potentially involved in orchestrating the host–pathogen interaction. It is expected that the findings from these analyses will set the stage for future research on Foa and provide a baseline for new control methods design to effectively manage Bayoud disease.

## Material and methods

### Genomic data acquisition

Currently, the FOSC includes more than 30 pathogenic and putatively non-pathogenic *formae speciales*. In our analysis, a total of 80 genomes corresponding to 30 pathogenic *formae speciales* including Foa (*formae speciales* of interest) were downloaded from NCBI and Joint Genome Institute’s MycoCosm databases. Detailed information (host, ID, strain and source information…..) of the strains employed in this study are denoted in Supplementary Data 1.

### Data quality assessment

The quality of the genome assemblies of the 80 fungal strains was evaluated using BUSCO (Benchmarking Universal Single-CopyOrthologs) v4.0.5^20^. In short, BUSCO estimates the completeness and redundancy of processed genomic data based on universal single-copy orthologs^20^. It uses Hidden Markov Models (HMM), HMMER v3.3.2 and tBLASTn to predict whether each (3) fragmented or (4) missing. For BUSCO analysis, we used here the Hypocreales specific single-copy orthologous genes from Fungi Odb version 10.

A total of 493 genes defined as orthologs were obtained from the BUSCO evaluation of 80 fungal genomes. To verify the presence of each BUSCO gene in the analyzed strains, the 80 genomes were annotated by AUGUSTUS version 3.4.0^21^ using *F. graminearum* as reference species, then each of the 493 BUSCO genes was aligned to the set of genes predicted by AUGUSTUS for each strain using BLASTn, the considered genes were those having identity >  = 97%. A gene was defined as single-copy ortholog if it is complete and present only once on the genome of each strain. Complete genes occurring twice or more were considered duplicate genes. Fragmented genes were BUSCO genes that cover less than 97% of gene predicted by AUGUSTUS. Genes absent in all strains were considered missing genes. Duplicated, fragmented, or missing genes in at least one strain were eliminated from the gene set. The strains in which the 493 genes are duplicated or absent or fragmented were also eliminated. The final data set retained corresponded to 51 fungal strains belonging to 30 *formae speciales* with 263 orthologous genes in a single copy for each strain.

### Phylogenomic analysis

For each strain, the 263 orthologous genes were concatenated in the same order using the CLC Genomic workbench. v20^22^; then aligned by MAFFT. Well-aligned regions were extracted using gBlocks. The final alignment was used as input for the MEGAX software^23^ to generate a phylogenetic tree using the Maximum Likelihood (ML) method with a Bootstrap of 1000. Bootstraps indicate how many times, out of 1000, the same branch was observed when repeating the phylogenetic construct on a re-sampled data set.

### Homologous protein identification

In addition to the predicted gene's sequences, the ab initio annotation by AUGUSTUS also provided the protein sequences corresponding to these genes. Indeed, ab initio annotation is based on the use of the Support Vector Machine (SVMs) algorithm and hidden Markov models (HMM) to predict genes and their structure in eukaryotic DNA sequences.

All of the predicted proteins from each genome of the 51 analyzed strains were aligned to the protein sequences of the other strains. The protein sequences of each strain were aligned to all the protein sequences of the remaining strains belonging to set N with N = 51. This alignment was performed using the BLASTp with an E-value < 10^−11^ and coverage >  = 97%. The tables resulting from the BLASTp analysis for each strain were filtered using the R package dplyr^24^ in order to keep only the lines where the identity between the aligned sequences is >  = 97%. Protein sequences found in a strain and occurring in other strains were defined as core sequences, thus representing the core genome. A sequence was considered unique if it was found only in one strain and not in the others. The set of unique sequences represent the unique genes. For the accessory genome, it was defined by the sequences common to only a part of the strains. On the basis of this BLASTp, three gene groups were defined for each strain: the core genes, the accessory genes and the unique genes. These three groups together constitute the pan genome.

### Functional annotation

The three protein sequence groups (core, accessory and unique) for each strain were classified into families using the Interproscan 5 software^25^, which gives an overview of the families that a protein belongs to as well as the domains and sites it contains. The files obtained from the Interproscan 5 analysis were filtered to eliminate false positives. In the case of proteins aligned to two or more families, the alignment result having the minimum E-value was chosen.

### KEGG pathways prediction

KEGG (Kyoto Encyclopedia of Genes and Genomes) is a collection of databases for understanding high-level functions and utilities of an organism linking genomic information with higher order functional information, by assigning the pathways where each protein of this organism is involved^26^. In our case, this prediction was performed by the Kofamscan software using the three protein sequence groups of each strain as input. The resulting list of KO codes (KEGG Orthology) was used to scan the KO database integrated in the KOALA-formatter software^27^. This allowed to extract KEGG pathways corresponding to the proteins of the used strains. KEGG pathways showing alignments of an E-value more than 10^−10^ were eliminated.

### Secretome and effectors prediction

To examine the secretome of the 51 strains used in our work, the SignalP v5.0^29^ and SecretomeP v1.0^28^ software were used to analyze their protein sequences in order to deduce the classical and non-classical secreted proteins for each strain. Then, the secretome sequences were submitted to the EffectorP v3.0^30^ software, which predicts the effector proteins in this sequence set. The effectors resulting from this prediction were organized into two categories: cytoplasmic effectors (which penetrate inside plant cells) and apoplastic effectors (which remain and act outside the plant cell).

### Detection of CAZymes (Carbohydrate-Active enZymes)

The protein sequences of the 51 selected strains were also the subject of CAZymes annotation using the run_dbcan v3.0 software^31^ coupled with the CAZyDB09242021 database. This software combines three state-of-the-art tools (DIAMOND, HMMER and eCAMI) to automatically identify CAZymes. Considered CAZymes were those identified by the three tools and having an e-value lower than 10^−10^.

### SIX proteins (secreted in xylem) prediction

The secretome of each strain was aligned to all the SIX sequences available on NCBI using the BLASTp with an E-value < 10^−11^ and a coverage > = 97%. Alignments with an identity ≥ 97% were selected to infer the different classes of SIX proteins present in each strain.

### Secondary metabolite gene clusters prediction

Gene clusters involved in secondary metabolite biosynthesis (BGC) were predicted using antiSMASH version 6.1^32^, which combines the MIGBIG, ClusterBlast and Subcluster Blast gene collection with the ClusterFinder and CASSIS (Cluster-border prediction based on transcription factor binding sites) algorithms. This combination provides an accurate identification of secondary metabolite gene clusters of known major chemical classes. It also offers a detailed sequence analysis. In our analysis, the genomic sequences of the 51 strains and their annotations were submitted to antiSMASH to inventory BGCs existing in the analyzed strains.

### Transposable elements prediction

Transposable elements (TE) of the 51 analyzed strains were predicted and annotated by the EDTA v1.3 software^33^, using the genomic sequences of these strains as input. It is a pipeline that allows the identification and classification of transposable elements based on a combination of programs: LTRharvest (v1.5.10), LTR_FINDER_parallel (v1.0), LTR_retriever (v2.6), Generic Repeat Finder (v1.0), TIR-Learner (v1.23), MITE-Hunter (v1.0) and HelitronScanner (v1.0). This combination results in the creation of a high-quality non-redundant TE library.

### Statistics and plot generation

The genome statistics (genome size and GC rate) were computed using Quast^83^ software; the other statistics (number of proteins and BUSCO genes) were obtained using the BUSCO^20^ and AUGUSTUS^21^ softwares described above. All the results obtained from the analyses carried out in this work were processed and filtered using the R package dplyr^24^ and custom codes. these codes are not yet available online. All figures representing the results of this work have been generated using the R graphic package ggplot2^84^.

## Supplementary Information

Supplementary Information 1.

Supplementary Information 2.

Supplementary Information 3.

Supplementary Information 4.

## Data Availability

The Foa genomes used in this study are available from Joint Genome Institute fungal genome portal MycoCosm (http://jgi.doe.gov/fungi) and National Center for Biotechnology Information (https://www.ncbi.nlm.nih.gov/). The accession numbers are JAAVJG000000000.1 (Foa 133) and JAKELM000000000.1 (Foa 9).
