# Publisher Correction: In silico comparative genomic analysis unravels a new candidate protein arsenal specifically associated with *Fusarium oxysporum* f. sp. *albedinis* pathogenesis

**DOI:** 10.1038/s41598-025-86905-z

**Published:** 2025-01-28

**Authors:** Hafida Ayada, Boutayna Dhioui, Hamid Mazouz, Abdelhay El harrak, Fatima Jaiti, Bouchra Ouhmidou, Mohammed Diouri, Mohieddine Moumni

**Affiliations:** 1 Biotechnology and Bioresources Valorization Laboratory, Biology Department, Faculty of Sciences, Moulay Ismail University of Meknès, Meknès, Morocco; 2 Biodiversity, Environment and Plant Protection Team, Faculty of Sciences and Technologies, Moulay Ismail University of Meknès, Meknès, Morocco; 3 Microbial Biotechnology and Bioactive Molecules Laboratory, Faculty of Sciences and Technologies, Sidi Mohammed Ben Abdellah University of Fez, Fez, Morocco

Correction to: *Scientific Reports* 10.1038/s41598-022-21858-1, published online 09 November 2022

The original version of this Article contained errors. In the Introduction,

“*Fusarium oxysporum* species complex (FOSC) is a ubiquitous group of pathogenic and putatively non-pathogenic soil-borne fungi. It is the most widespread in nature, it colonizes all soil types (cultivated and uncultivated soils) in all continents except Antarctica^1,1,1^.”

now reads:

“*Fusarium oxysporum* species complex (FOSC) is a ubiquitous group of pathogenic and putatively non-pathogenic soil-borne fungi. It is the most widespread in nature, it colonizes all soil types (cultivated and uncultivated soils) in all continents except Antarctica^1,2,3^.”

“In Morocco, Fusarium wilt due to Foa has caused the progressive disappearance of high quality and world renown date cultivars, mainly Mejhool^8,8^.”

now reads:

“In Morocco, Fusarium wilt due to Foa has caused the progressive disappearance of high quality and world renown date cultivars, mainly Mejhool^8,9^.”

“Regarding Foa, despite the fact that it has been the subject of several investigations for a long time^15,15,15^, information on its genomics has remained rather quite limited until now. Currently, progress on Foa could be achieved now that its genome is available in databases^18,18^.”

now reads:

“Regarding Foa, despite the fact that it has been the subject of several investigations for a long time^15,16,17^, information on its genomics has remained rather quite limited until now. Currently, progress on Foa could be achieved now that its genome is available in databases^18,19^.”

In the Results section,

“The two Foa genomes correspond to two strains originally isolated from infected date palms^18,18^.”

now reads:

“The two Foa genomes correspond to two strains originally isolated from infected date palms^18,19^.”

“Within the FOSC, some pathogens secrete small effectors in the xylem sap of the host plant^41,41^.”

now reads:

“Within the FOSC, some pathogens secrete small effectors in the xylem sap of the host plant^41,42^.”

In the Discussion section,

“These are known as omnipresent proteins in all vital functions and constitute the largest family of secondary transporters^47,47^. They are involved in essential cellular functions, such as nutrient uptake and metabolite extrusion^48,48^.”

now reads:

“These are known as omnipresent proteins in all vital functions and constitute the largest family of secondary transporters^47,48^. They are involved in essential cellular functions, such as nutrient uptake and metabolite extrusion^48,49^.”

“The latter are used by fungi as rapid adaptation strategies in several ecological niches^52,52,52^, they are involved in the degradation processes of the plant cell wall^55^.”

now reads:

“The latter are used by fungi as rapid adaptation strategies in several ecological niches^52,53,54^, they are involved in the degradation processes of the plant cell wall^55^.”

“A previous studies revealed that date palm is almost entirely composed (92%) of linear mannan (high molecular weight molecule)^75^, giving it a hardness, especially in seeds, to protect it from mechanical damage^76,76^.”

now reads:

“A previous studies revealed that date palm is almost entirely composed (92%) of linear mannan (high molecular weight molecule)^75^, giving it a hardness, especially in seeds, to protect it from mechanical damage^76,77^.”

The original Article has been corrected.

