# Supplementary material for: In silico comparative genomic analysis unravels a new candidate protein arsenal specifically associated with Fusarium oxysporum f. sp. albedinis pathogenesis
Source: Sci Rep. 2022 Nov 9;12:19098. doi: 10.1038/s41598-022-21858-1 (PMC9646873; doi:10.1038/s41598-022-21858-1)
Supplement: Supplementary file 1 — Supplementary Information 1. [file 41598_2022_21858_MOESM1_ESM.pdf]

Supplementary data 1-Table 1 : strains of the formea speciales belonging to Fusarium oxysporum species complex used in this study				
**Strains**	**Accession Number**	**Host**	**Isolation_Source**	**Country**
Fusarium oxysporum. f.sp.albedinis 133	JAAVJG000000000.1	date palm	wilt plant tissue	Morocco
Fusarium oxysporum. f.sp.albedinis 9	JAKELM000000000.1	date palm	diseased palm tree	Morocco
Fusarium oxysporum. f.sp.apii 207	JAAOOO000000000.1	Apium graveolens	agricultural field	USA
Fusarium oxysporum. f.sp.apii NRRL38295	JAAOOP000000000.1	Apium graveolens	agricultural field	USA
Fusarium oxysporum. f.sp.cepae FoC125	MRCV00000000.1	Allium cepa	Organic material	United Kingdom
Fusarium oxysporum. f.sp.cepae FoCFus2	MRCU00000000.1	Allium cepa	Organic material	United Kingdom
Fusarium oxysporum. f.sp.ciceris 38-1	MEHF00000000.1	Chickpea		India
Fusarium oxysporum. f.sp.Conglutinans 58385	NRHZ00000000.1	Brassica oleracea	root	USA
Fusarium oxysporum. f.sp.conglutinans FGL03-6	NRIA00000000.2	Brassica oleracea	root	China
Fusarium oxysporum. f.sp.conglutinans race 254008	AGNF00000000.1			
Fusarium oxysporum. f.sp.Coriandrii 3-2	JAAOON000000000.1	Coriandrum sativum	agricultural field	USA
Fusarium oxysporum. f.sp.Coriandrii G306	JAAOOM000000000.1	Coriandrum sativum	agricultural field	USA
Fusarium oxysporum. f.sp.cubense 160527	SRMI00000000.1	banana	infected banana	Japan
Fusarium oxysporum. f.sp.cubense C1HIR9889	MBFV00000000.1	Musa acuminata	roots	Malaysia
Fusarium oxysporum. f.sp.cucumerinum Foc018	MABM00000000.1			Greece
Fusarium oxysporum. f.sp.cucumerinum Foc030	MABN00000000.1			Israel
Fusarium oxysporum. f.sp.fragariae 160609	WINB00000000.1	Fragaria x ananassa	plant matter	South Korea
Fusarium oxysporum. f.sp.fragariae BRIP5168a	WIMZ00000000.1	Fragaria x ananassa	plant matter	Australia
Fusarium oxysporum. f.sp.gladioli G2	NJCL00000000.1	Gladiolus	Gladiolus sp	France
Fusarium oxysporum. f.sp.gladioli G76	NJCK00000000.1	Gladiolus	Gladiolus sp	Italy
Fusarium oxysporum. f.sp.koaei 44	CP052908.1-CP052907.1	Acacia koa	Plant	USA
Fusarium oxysporum. f.sp.lagenariae 01-03008	NJCJ00000000.1	Lagenaria	Lagenaria sp	Japan
Fusarium oxysporum. f.sp.lagenariae Lag3-1	NJCH00000000.1	Lagenaria	Lagenaria sp	Japan

Fusarium oxysporum. f.sp.lilii Fol39	NJCF00000000.1	Lilium	Lilium sp	Netherlands
Fusarium oxysporum. f.sp.lini F282	JABJUB000000000.1	Linum usitatissimum	flax root	Russia
Fusarium oxysporum. f.sp.lini F324	JABJUD000000000.1	Linum usitatissimum	flax root	Russia
Fusarium oxysporum. f.sp.lycopersici Fol014	MALJ00000000.1			USA
Fusarium oxysporum. f.sp.lycopersici Fol074	MALS00000000.1			USA
Fusarium oxysporum. f.sp.matthiolae PHW726	WJXY00000000.1	Matthiola incana		USA
Fusarium oxysporum. f.sp.medicaginis Fom-5190a	LSNI00000000.1	Medicago sativa	wilting leaves in a field of Medicago sativa	Australia
Fusarium oxysporum. f.sp.melongenae J-71	NJCC00000000.1		Solanum melongena	
Fusarium oxysporum. f.sp.melonis 26406	AGNE00000000.1			
Fusarium oxysporum. f.sp.melonis Fom005	MALY00000000.2			Spain
Fusarium oxysporum. f.sp.momordicae 90NF2-1	NJCA00000000.1	Momordica	Momordica sp	Japan
Fusarium oxysporum. f.sp.momordicae NRRL26413	NJCB00000000.1	Momordica	Momordica sp	Taiwan
Fusarium oxysporum. f.sp.narcissi N139	MQTW00000000.1	Narcissus	plant bulb	United Kingdom
Fusarium oxysporum. f.sp.narcissi Na5	NJCV00000000.1	Narcissus	Narcissus sp	Netherlands
Fusarium oxysporum. f.sp.nicotianae Ft-1512	NJCU00000000.1	Nicotiana	Nicotiana sp	USA
Fusarium oxysporum. f.sp.nicotianae Ft-Rob	NJBX00000000.1	Nicotiana	Nicotiana sp	USA
Fusarium oxysporum. f.sp.niveum Fon002	MALA00000000.1			Israel
Fusarium oxysporum. f.sp.niveum R1	JACRUY000000000.1	Citrullus lanatus	Watermelon plant	USA
Fusarium oxysporum. f.sp.pisi HDV247	AGBI00000000.1			
Fusarium oxysporum. f.sp.radicis-cucumerinum Forc024	MABR00000000.1			Greece
Fusarium oxysporum. f.sp.radicis-cucumerinum Forc031	MABS00000000.1			Greece
Fusarium oxysporum. f.sp.radicis-lycopersici 26381	AGNB00000000.1			
Fusarium oxysporum. f.sp.raphani 54005	AGNG00000000.1			
Fusarium oxysporum. f.sp.spinaciae Fus254	JAALGI000000000.1	Spinacia oleracea	spinach	USA

Fusarium oxysporum. f.sp.spinaciae MF15	JABCQZ000000000.1	Spinacia oleracea		USA
Fusarium oxysporum. f.sp.tulipaeTu67	NJBS00000000.1	tulip bulb	infected tulip bulb	Netherlands
Fusarium oxysporum. f.sp.Vasinfectum LA1E	VINN00000000.1		Mycellium	USA
Fusarium oxysporum. f.sp.Vasinfectum NRRL31665	JAANYM000000000.1	cotton	cotton roots	
