# Supplementary material for: In silico comparative genomic analysis unravels a new candidate protein arsenal specifically associated with Fusarium oxysporum f. sp. albedinis pathogenesis
Source: Sci Rep. 2022 Nov 9;12:19098. doi: 10.1038/s41598-022-21858-1 (PMC9646873; doi:10.1038/s41598-022-21858-1)
Supplement: Supplementary file 2 — Supplementary Information 2. [file 41598_2022_21858_MOESM2_ESM.pdf]

**Supplementary data 2-Table 1 : predicted gene content of the analyzed formea speciales belonging to Fusarium oxysporum species complex.**								
**Stains**	**Pan-genome**		**Core-genome**		**Accessory genome**		**Unique-genome**	
	**Genes withe Unknown function**	**Genes withe Known function**	**Genes withe Unknown function**	**Genes withe Known function**	**Genes withe Unknown function**	**Genes withe Known function**	**Genes withe Unknown function**	**Genes withe Known function**
F.o.f.sp.apiiNRRL38295	5977	13308	939	4269	4754	8585	284	454
F.o.f.sp.coriandrii3-2	6057	13366	940	4262	5036	9030	81	74
F.o.f.sp.coriandriiGL306	5974	13413	941	4273	4943	9014	90	126
F.o.f.sp.vasinfectumLA1E	4818	11655	957	4272	3405	6457	706	676
F.o.f.sp.spinaciaeFus254	5107	12348	956	4272	3672	7183	479	893
F.o.f.sp.momordicae90NF2-1	4721	11132	945	4258	3670	6783	104	91
F.o.f.sp.momordicaeNRRL26413	4807	11181	945	4260	3765	6839	97	82
F.o.f.sp.lagenariae01-03008	5105	11509	939	4266	3764	6851	397	392
F.o.f.sp.fragariae160609	5122	11526	943	4259	3840	6972	339	295
F.o.f.sp.niveumR1	6126	13884	945	4257	4151	6885	1017	2742
F.o.f.sp.spinaciaeMF15	5379	11706	935	4249	3818	6852	623	605
F.o.f.sp.nicotianaeFt-1512	4665	11226	950	4249	3386	6644	327	333
F.o.f.sp.vasinfectumNRRL31665	5112	11760	979	4370	3574	6714	559	676
F.o.f.sp.liliiFol39	5054	11530	936	4254	3540	6720	576	556
F.o.f.sp.fragariaeBRIP5168a	4255	10721	945	4246	3058	6233	252	242
F.o.f.sp.LagenariaeLag3-1	4387	10819	927	4235	2953	6101	503	483
F.o.f.sp.koae44	3786	10045	939	4233	2360	5322	487	490
F.o.f.sp.conglutinans58385	5845	11789	951	4278	4601	7356	292	155
F.o.f.sp.conglutinansFGL03-6	5629	13077	944	4266	4329	8002	355	809
F.o.f.sp.conglutinansrace254008	5327	11488	951	4276	4240	7126	135	86
F.o.f.sp.melonisFom005	5201	11713	942	4261	3663	6799	595	653
F.o.f.sp.niveumFon002	5017	11251	953	4268	3444	6450	616	533

F.o.f.sp.cucumerinumFoc018	5671	11964	948	4285	4341	7362	369	317
F.o.f.sp.cucumerinumFoc030	5575	11918	950	4288	4317	7362	304	268
F.o.f.sp.Raphani54005	5344	11401	957	4279	3771	6456	614	666
F.o.f.sp.MelongenaeJ-71	4948	11390	954	4256	3346	6395	645	739
F.o.f.sp.pisiHDV247	5206	12075	951	4263	3763	7168	490	644
F.o.f.sp.liniF282	4533	11063	994	4537	3271	6142	254	384
F.o.f.sp.liniF324	4567	10812	980	4360	3256	6020	305	432
F.o.f.sp.cubense160527	4584	11277	934	4208	2663	5950	986	1119
F.o.f.sp.ciceris38-1	5113	11256	960	4248	2510	5251	1642	1757
F.o.f.sp.gladioliG76	5457	11703	949	4231	3143	5971	1362	1501
F.o.f.sp.lycopersiciFol014	4839	11103	923	4222	3719	6722	195	159
F.o.f.sp.lycopersiciFol074	5065	11298	925	4219	3862	6889	276	190
F.o.f.sp.Melonis26406	4047	12712	925	4222	3808	7238	821	1252
F.o.f.sp.albedinis133	5522	11938	931	4247	4380	7504	209	187
F.o.f.sp.albedinis9	5835	13679	926	4232	4489	8384	420	1063
F.o.f.sp.matthiolaePHW726	5299	12175	917	4233	3928	7219	454	723
F.o.f.sp.tulipaeTu67	5096	11618	916	4228	3679	6804	499	586
F.o.f.sp.apii207	5919	13443	901	4247	3863	7523	1139	1689
F.o.f.sp.radicis-cucumerinumForc024	4468	11037	914	4223	3493	6713	59	101
F.o.f.sp.radicis-cucumerinumForc031	4484	11021	912	4225	3492	6707	78	89
F.o.f.sp.gladioliG2	4991	11571	911	4226	3642	6848	435	497
F.o.f.sp.cepaeFoC125	4872	11458	919	4240	3850	7091	103	127
F.o.f.sp.cepaeFoCFus2	4579	11994	911	4233	3552	7354	116	407
F.o.f.sp.medicaginisFom-5190a	4699	11184	930	4237	3290	6458	478	489
F.o.f.sp.Radicis-lycopersici26381	4538	11040	914	4208	3102	6211	522	621
F.o.f.sp.nicotianaeFt-Rob	4507	11132	914	4226	3247	6450	346	456
F.o.f.sp.narcissiN139	5992	12354	916	4230	4562	7654	511	470
F.o.f.sp.narcissiNa5	5481	11896	913	4233	4158	7300	400	363
F.o.f.sp.cubenseC1HIR9889	4524	10630	997	4330	2063	4711	1462	1589

**Supplementary data 2-Table 2 : predicted core-gene families of *Fusarium oxysporum*. f. sp *albedinis* strains.**		
**Gene families**	***Fusarium oxysporum*. f. sp. *albedinis 133***	***Fusarium oxysporum*. f. sp. *albedinis 9***
	**Gene count**	**Gene count**
Transketolase,thiaminediphosphatebindingdomain	3	3
PeptidasefamilyM28	15	15
Vacuolarmembrane-associatedproteinIml1	1	1
Ubiquitincarboxyl-terminalhydrolase	21	21
NAT,N-acetyltransferase,ofN-acetylglutamatesynthase	1	1
Fasciclindomain	5	3
ABCtransporter	44	44
Ferricreductaseliketransmembranecomponent	7	7
Nse4C-terminal	1	1
Acyl-CoAdehydrogenase,C-terminaldomain	11	11
Calcium-dependentchannel,7TMregion,putativephosphate	5	5
DEAD/DEAHboxhelicase	36	37
D-isomerspecific2-hydroxyaciddehydrogenase,NADbindingdomain	17	19
MajorFacilitatorSuperfamily	207	200
AMP-bindingenzyme	39	39
non-haemdioxygenaseinmorphinesynthesisN-terminal	3	3
Fattyaciddesaturase	7	7
AlcoholdehydrogenaseGroES-likedomain	29	29
Mre11DNA-bindingpresumeddomain	1	1
Copperamineoxidase,enzymedomain	7	7
DNApolymerasealpha/epsilonsubunitB	3	3
RNAligase	5	5
DnaJCterminaldomain	9	9
AdaptinNterminalregion	11	11
Ubiquitin-bindingdomain	1	1

NMDAreceptor-regulatedprotein1	1	1
Ankyrinrepeats(3copies)	11	11
BCS1Nterminal	5	5
Vacuolarsortingprotein9(VPS9)domain	3	3
Proteinofunknownfunction(DUF933)	1	1
Transferrinreceptor-likedimerisationdomain	5	5
Domainofunknownfunction(DUF3402)	1	1
Fungalspecifictranscriptionfactordomain	127	127
Glycosylhydrolasesfamily43	17	16
RegulatorofGproteinsignalingdomain	3	3
ATP-NADkinaseC-terminaldomain	3	3
Domainofunknownfunction(DUF1929)	3	3
TranscriptionfactorTFIID(orTATA-bindingprotein,TBP)	1	1
Mediatorcomplexsubunit13C-terminaldomain	1	1
NUC153domain	3	3
ElongationfactorTuGTPbindingdomain	15	15
26SproteasomesubunitRPN7	3	1
EFhandassociated	1	1
Formamidopyrimidine-DNAglycosylaseH2THdomain	1	1
FADbindingdomain	67	65
E1-E2ATPase	11	11
C2HE/C2H2/C2HCzinc-bindingfinger	1	1
Helicaseassociateddomain(HA2)	7	7
Thiolase,N-terminaldomain	9	11
Importinbetabindingdomain	1	1
ssDNA-bindingdomainoftelomereprotectionprotein	1	1
Surfeitlocusprotein6	1	1
Hint-domain	1	1
Acetyl-CoAhydrolase/transferaseN-terminaldomain	1	1
Mitochondrialcarrierprotein	60	60

Dynaminfamily	7	7
MethyltransferaseinvolvedinWilliams-Beurensyndrome	1	1
Thioredoxin-like	5	5
Cupin	27	27
PQlooprepeat	7	7
SNF2-relateddomain	19	19
Enolase,C-terminalTIMbarreldomain	1	1
CoenzymeAtransferase	1	1
Zincfinger,C2H2type	21	21
TATAelementmodulatoryfactor1TATAbinding	1	1
LIMdomain	1	1
PCIdomain	13	13
Dynamincentralregion	3	3
Glutaredoxin	5	5
Proteinkinasedomain	102	103
HAT(Half-A-TPR)repeat	1	1
snoRNAbindingdomain,fibrillarin	3	3
C2domain	11	11
NrapproteinPAP/OAS1-likedomain5	1	1
Histidinekinase-,DNAgyraseB-,andHSP90-likeATPase	15	15
PIGA(GPIanchorbiosynthesis)	1	1
Sodium/calciumexchangerprotein	5	5
ZincfingerC-x8-C-x5-C-x3-Htype(andsimilar)	5	5
Syntaxin	7	7
Domainofunknownfunction(DUF3395)	1	1
Spa2homologydomain(SHD)ofGIT	1	1
OxidoreductaseNAD-bindingdomain	5	5
ZIPZinctransporter	15	15
RNApolymeraseRpb1,domain1	3	3
WDdomain,G-betarepeat	75	73

Thg1Cterminaldomain	1	1
Prenyltransferaseandsqualeneoxidaserepeat	1	1
Cid1familypolyApolymerase	3	3
ERmembraneproteincomplexsubunit1,C-terminal	1	1
BiotinandThiaminSynthesisassociateddomain	1	1
Bromodomain	7	7
Tubulin/FtsZfamily,GTPasedomain	3	3
PhoDrelatedphosphatase	1	1
Centrosomemicrotubule-bindingdomainofCep57	1	1
ATPasefamilyassociatedwithvariouscellularactivities(AAA)	33	33
FFdomain	1	1
Domainofunknownfunction(DUF4110)	1	1
Sulfatepermeasefamily	5	5
UTP15Cterminal	1	1
Maintenanceofmitochondrialstructureandfunction	5	5
Non-repetitive/WGA-negativenucleoporinC-terminal	1	1
AFG1-likeATPase	1	1
Oxidoreductasefamily,NAD-bindingRossmannfold	13	11
FirstLongindomainofFUZ,MON1andHPS1	1	1
Tryptophansynthasealphachain	1	1
Adenylatekinase	5	5
Zinc-fingerdomainofmonoamine-oxidaseArepressorR1	1	1
Proteinprenyltransferasealphasubunitrepeat	5	5
Pex2/Pex12aminoterminalregion	3	3
Xrn1helicaldomain	1	1
Actincytoskeleton-regulatorycomplexproteinEND3	1	1
ReplicationproteinACterminal	1	1
mRNAcleavageandpolyadenylationfactorCLP1P-loop	1	1
Lonprotease(S16)C-terminalproteolyticdomain	3	3
Carbamoyl-phosphatesynthaseLchain,ATPbindingdomain	7	7

GHMPkinasesNterminaldomain	5	5
PPRrepeatfamily	3	3
GTP1/OBG	1	1
RNApolymeraseRpb5,C-terminaldomain	1	1
WSCdomain	9	9
Kelchmotif	9	9
tRNAsynthetaseclassIIcoredomain(G,H,P,SandT)	7	7
Pre-mRNA-splicingfactorSF3acomplexsubunit2(Prp11)	1	1
GNL3L/Grn1putativeGTPase	1	1
RNBdomain	5	5
Transientreceptorpotential(TRP)ionchannel	9	9
Dip2/Utp12Family	5	5
Cationeffluxfamily	11	11
Phosphatidylinositol3-and4-kinase	5	5
RibosomalproteinL6	5	5
V-ATPasesubunitH	1	1
XPGI-region	1	1
Phosphoglucomutase/phosphomannomutase,alpha/beta/alphadomainIII	1	0
AutophagyproteinATG17-likedomain	3	3
Man1-Src1p-C-terminaldomain	1	1
Hingedomainofcleavagestimulationfactorsubunit2	1	1
Endoplasmicreticulumvesicletransporter	3	3
Sel1repeat	5	5
Proteasomesubunit	19	19
RNApolymeraseRpb3/Rpb11dimerisationdomain	3	3
Zinc-fingerofC2H2type	1	1
ThiFfamily	7	7
Hydroxymethylglutaryl-coenzymeAreductase	1	1
Cullinfamily	7	7
SaccharopinedehydrogenaseC-terminaldomain	1	1

8-oxoguanineDNAglycosylase,N-terminaldomain	1	1
Actin	13	13
RibosomalS13/S15N-terminaldomain	1	1
Synaptobrevin	5	5
Asparaginase	7	7
Adenosinedeaminase	5	5
C3HCzincfinger-like	1	1
C2H2typezinc-finger(2copies)	1	1
Ariadnedomain	1	1
XPGN-terminaldomain	5	5
tRNAsynthetasesclassI(EandQ),catalyticdomain	5	5
Ribonucleotidereductase,barreldomain	1	1
ABC-2typetransporter	9	9
Wyosinebaseformation	1	1
Exocystcomponent84C-terminal	1	1
pre-mRNAsplicingfactorcomponent	1	1
CIDdomain	5	5
DNAgyrase/topoisomeraseIV,subunitA	1	1
40SribosomebiogenesisproteinTsr1andBMS1C-terminal	3	3
Proteinofunknownfunction(DUF2034)	1	1
CellmorphogenesisN-terminal	1	1
Exportin1-likeprotein	1	1
chorismatebindingenzyme	3	3
PPIC-typePPIASEdomain	1	1
Vta1like	1	1
DHHCpalmitoyltransferase	7	7
DNApolymerasealphasubunitBN-terminal	1	1
UDP-glucose/GDP-mannosedehydrogenasefamily,centraldomain	3	3
XFPN-terminaldomain	3	3
Catalase	3	3

Thiaminepyrophosphateenzyme,N-terminalTPPbindingdomain	5	5
MgsAAAA+ATPaseCterminal	1	1
Cofilin/tropomyosin-typeactin-bindingprotein	7	7
tRNAsynthetasesclassI(R)	1	1
Pirin	1	1
Subtilasefamily	5	5
SecY	1	1
IPPtransferase	1	1
Dak1domain	1	1
TAF6C-terminalHEATrepeatdomain	1	1
FADdependentoxidoreductase	23	23
Armadillo/beta-catenin-likerepeat	1	1
NAD(P)transhydrogenasebetasubunit	1	1
Adaptorcomplexesmediumsubunitfamily	5	5
TemperaturedependentproteinaffectingM2dsRNAreplication	1	1
UncharacterizedproteinfamilyUPF0029	3	3
ATPsynthasealpha/betafamily,nucleotide-bindingdomain	1	1
GMCoxidoreductase	13	13
Vps5Cterminallike	3	3
EF-handdomainpair	5	5
Aconitasefamily(aconitatehydratase)	3	3
PWIdomain	3	3
HomeoboxKNdomain	5	5
Proteinofunknownfunction(DUF917)	1	1
Nse1non-SMCcomponentofSMC5-6complex	1	1
Transcription-silencingproteinClr2	1	1
26SproteasomeregulatorysubunitRPN2C-terminaldomain	1	1
Oxidoreductasemolybdopterinbindingdomain	1	1
CationtransportingATPase,C-terminus	5	5
EarlytranscriptionelongationfactorofRNApolII,NGNsection	1	1

Sec7domain	3	3
Pre-mRNAsplicingfactor	3	3
Beige/BEACHdomain	1	1
Malicenzyme,NADbindingdomain	3	3
Metallo-beta-lactamasesuperfamilydomain	1	1
NB-ARCdomain	1	1
RequiredfornucleartransportofRNApolIIC-terminus1	1	1
Domainofunknownfunction(DUF4187)	1	1
PRMT5TIMbarreldomain	1	1
Multicopperoxidase	7	7
Flavin-bindingmonooxygenase-like	22	22
Aminoacidkinasefamily	1	1
ExocystcomplexcomponentSec10	3	3
Eukaryoticglutathionesynthase,ATPbindingdomain	1	1
RhoGAPdomain	11	11
COMPASS(ComplexproteinsassociatedwithSet1p)componentN	1	1
P5-typeATPasecationtransporter	1	1
RibosomalproteinL4/L1family	1	1
SEPdomain	1	1
SF3a60/Prp9C-terminal	1	1
CTPsynthaseN-terminus	1	1
ERCC3/RAD25/XPBC-terminalhelicase	1	1
F-box-like	21	21
Histidinebiosynthesisprotein	3	3
Oxysterol-bindingprotein	7	7
CTLH/CRAC-terminaltoLisHmotifdomain	7	7
CNHdomain	1	1
ATPsynthasesubunitC	7	7
G-patchdomain	5	5
Proteinofunknownfunction(DUF3433)	9	9

Phospholipid-translocatingP-typeATPaseC-terminal	7	7
Thioredoxin	15	15
Phytochromeregion	1	1
Glycosylhydrolasesfamily32Cterminal	1	1
Hexokinase	5	5
RegulatorofTy1transpositionprotein107BRCTdomain	1	1
Pyridinenucleotide-disulphideoxidoreductase	21	19
tRNAsynthetasesclassI(M)	1	1
TFA2Wingedhelixdomain2	1	1
Cytochromeb5-likeHeme/Steroidbindingdomain	7	7
Domainofunknownfunction(DUF1899)	1	1
F-actincappingproteinalphasubunit	1	1
Celldivisionproteinanillin	1	1
Inositol-pentakisphosphate2-kinase	1	1
ABC1atypicalkinase-likedomain	5	5
Apoptosis-antagonizingtranscriptionfactor,C-terminal	1	1
Chalconeandstilbenesynthases,C-terminaldomain	1	1
Rad4transglutaminase-likedomain	5	5
linkerbetweenRRM2andRRM3domainsinRBM39protein	1	1
Clusteredmitochondria	1	1
Forkheaddomain	5	5
Tubulindomain	1	1
FGGYfamilyofcarbohydratekinases,C-terminaldomain	7	7
BING4CT(NUC141)domain	1	1
RasGEFdomain	7	7
Cyclophilintypepeptidyl-prolylcis-transisomerase/CLD	9	9
Dcp2,boxAdomain	1	1
PHdomain	5	5
Cdc37Nterminalkinasebinding	1	1
Polysaccharidelyasefamily4,domainIII	1	1

Domainofunknownfunction(DUF3535)	1	1
RecF/RecN/SMCNterminaldomain	7	7
X-Prodipeptidyl-peptidase(S15family)	1	1
Pleckstrinhomologydomain	1	1
H2C2zincfinger	1	1
CBSdomain	1	1
ATPdependentDNAligasedomain	5	5
Tetratricopeptiderepeat	11	11
Glycosidehydrolasefamily5C-terminaldomain	3	3
MutSdomainV	3	3
non-SMCmitoticcondensationcomplexsubunit1,N-term	1	1
NLIinteractingfactor-likephosphatase	7	7
FADlinkedoxidases,C-terminaldomain	3	3
Velvetfactor	1	1
Cytochromedomainofcellobiosedehydrogenase	3	3
Glycosylhydrolasefamily3C-terminaldomain	11	11
Anaphasepromotingcomplexsubunit8/Cdc23	1	1
Utp13specificWD40associateddomain	1	1
AIRcarboxylase	1	1
Asparaginase,N-terminal	3	3
Histone-bindingproteinRBBP4orsubunitCofCAF1complex	3	3
Signalpeptidasesubunit	1	1
Originrecognitioncomplex(ORC)subunit5C-terminus	1	1
AhpC/TSAfamily	5	5
Vacuolarsortingprotein39domain1	1	1
6-phosphofructo-2-kinase	5	5
Mad3/BUB1homologyregion1	1	1
ATPphosphoribosyltransferase	1	1
Iontransportprotein	3	3
D-aminopeptidase,domainB	1	1

3-hydroxyacyl-CoAdehydrogenase,NADbindingdomain	3	3
Sulfatase	13	13
PeptidasefamilyM41	3	3
Pyrroline-5-carboxylatereductasedimerisation	3	3
NLE(NUC135)domain	1	1
CAP-Glydomain	3	3
Lyase	5	5
RibosomalproteinL10	3	3
twinBRCTdomain	1	1
Cullinbinding	1	1
Sortingnexin8/Mvp1BARdomain	1	1
Familyofunknownfunction(DUF5923)	3	3
Glycosylhydrolasesfamily38N-terminaldomain	1	1
eRF1domain1	1	1
TIP49P-loopdomain	3	3
PHD-zinc-fingerlikedomain	3	3
RAMsignallingpathwayprotein	1	1
RNApolymeraseIIIsubunitRPC82	1	1
Mitochondrialbranched-chainalpha-ketoaciddehydrogenasekinase	5	5
RibosomalproteinS5,C-terminaldomain	1	1
InitiationfactoreIF2gamma,Cterminal	1	1
CSLzincfinger	1	1
Phophatidylserinedecarboxylase	3	3
AAAdomain(Cdc48subfamily)	5	5
Mis6	1	1
Alphaamylase,C-terminalall-betadomain	1	1
TFIIHp62subunit,N-terminaldomain	1	1
Transketolase,pyrimidinebindingdomain	1	1
tRNAsynthetasesclassI(I,L,MandV)	5	5
Cupin-likedomain	5	5

Domainofunknownfunction(DUF3384)	1	1
DEAD_2	1	1
DDHDdomain	1	1
pre-mRNAprocessingfactor3(PRP3)	1	1
RNApolymeraseRpb1,domain5	1	1
Domainofunknownfunction(DUF1932)	1	1
Glyceraldehyde3-phosphatedehydrogenase,C-terminaldomain	1	1
SART-1family	1	1
Aspartate/ornithinecarbamoyltransferase,carbamoyl-Pbindingdomain	1	1
Smallsubunitofacetolactatesynthase	1	1
CAS/CSEprotein,C-terminus	1	1
Cse1	1	1
ELMO/CED-12family	1	1
Aminoacidpermease	23	23
AAAdomain	11	11
EukaryoticDNAtopoisomeraseI,catalyticcore	1	1
KHdomain	7	7
UPF0113Pre-PUAdomain	1	1
Anticodonbindingdomain	3	3
RNArecognitionmotif.(a.k.a.RRM,RBD,orRNPdomain)	41	41
PULdomain	1	1
LD-carboxypeptidaseC-terminaldomain	1	1
Dioxygenase	9	9
Glutamate/Leucine/Phenylalanine/Valinedehydrogenase	3	3
DNApolymerasefamilyB	5	5
Zinc-bindingdehydrogenase	23	23
Oxidoreductasefamily,C-terminalalpha/betadomain	1	1
Glycosylhydrolasesfamily2,TIMbarreldomain	1	1
Fructose-1-6-bisphosphatase,C-terminaldomain	1	1
Righthandedbetahelixregion	1	1

BacteroidetesVLRF1releasefactor	1	1
MmgE/PrpDN-terminaldomain	1	1
FADbindingdomainofDNAphotolyase	3	3
FAD-bindingdomain	3	3
CytochromeP450	39	37
OxidoreductaseFAD-bindingdomain	1	1
SWIB/MDM2domain	3	3
PrpFprotein	1	1
Ringfingerdomain	7	7
Semialdehydedehydrogenase,dimerisationdomain	1	1
Domainofunknownfunction(DUF1996)	7	7
alpha/betahydrolasefold	47	45
Iron/manganesesuperoxidedismutases,C-terminaldomain	3	3
Calcineurin-likephosphoesterase	17	15
RING/Uboxlikezinc-bindingdomain	1	1
Ionchannel	1	1
ABCtransportertransmembraneregion	17	17
MannitoldehydrogenaseC-terminaldomain	1	1
HydrolyticATPbindingsiteofdyneinmotorregion	1	1
Proteinofunknownfunction(DUF3955)	1	1
Ribosome-associatedcomplexheaddomain	1	1
Shikimatedehydrogenasesubstratebindingdomain	3	3
Synaptotagmin-likemitochondrial-lipid-bindingdomain	1	1
DNA-dependentRNApolymerase	1	1
CobW/HypB/UreG,nucleotide-bindingdomain	7	7
SacIhomologydomain	7	7
Chitinsynthase	16	16
Phosphatidylserinedecarboxylase	3	3
Cellulase(glycosylhydrolasefamily5)	9	9
Nup133Nterminallike	1	1

SterolmethyltransferaseC-terminal	1	1
Galactose-1-phosphateuridyltransferase,N-terminaldomain	1	1
5'-AMP-activatedproteinkinasebetasubunit,interactiondomain	1	1
EXSfamily	1	1
Pumilio-familyRNAbindingrepeat	5	5
Histidine-specificmethyltransferase,SAM-dependent	3	3
GammatubulincomplexcomponentN-terminal	5	5
Sodium:neurotransmittersymporterfamily	3	3
Cyclin	13	13
Commoncentraldomainoftyrosinase	5	5
RNApolymeraseRpb2,domain6	3	3
RhoGEFdomain	3	3
NADH-ubiquinoneoxidoreductasecomplexI,21kDasubunit	1	1
DipeptidylpeptidaseIV(DPPIV)N-terminalregion	1	1
ProteinsimilartoCwfJC-terminus1	3	3
RegioninClathrinandVPS	3	3
C-terminalregionofeIF3h	1	1
2Fe-2Siron-sulfurclusterbindingdomain	3	3
ALG6,ALG8glycosyltransferasefamily	1	1
RNApolymeraseIIIsubunitRpc25	1	1
Dos2-interactingtranscriptionregulatorofRNA-Pol-II	1	1
N-terminaldomainofoxidoreductase	1	1
Glycosylhydrolasesfamily18	11	9
ATP-dependentproteaseLa(LON)substrate-bindingdomain	1	1
CDK-activatingkinaseassemblyfactorMAT1	1	1
impB/mucB/samBfamily	1	1
NUDIXdomain	7	7
CAF1familyribonuclease	1	1
tRNAsynthetasesclassII(A)	1	1
TranscriptionfactorS-II(TFIIS),centraldomain	1	1

SRI(Set2Rpb1interacting)domain	1	1
Sas10C-terminaldomain	1	1
FungalZn(2)-Cys(6)binuclearclusterdomain	45	45
Domainofunknownfunction(DUF3543)	1	1
Pyridoxal-phosphatedependentenzyme	13	13
Ribosomal_S17N-terminal	1	1
Spc7kinetochoreprotein	1	1
Prefoldinsubunit	5	5
Mitochondrialinnermembraneprotein	1	1
Outermitochondrialmembranetransportcomplexprotein	1	1
Kinesinmotordomain	15	15
Acetohydroxyacidisomeroreductase,NADPH-bindingdomain	1	1
GNATacetyltransferase2	1	1
Amidohydrolasefamily	19	19
Proteinofunknownfunction(DUF3419)	1	1
tRNAsynthetasesclassI(WandY)	3	3
Nitrogenpermeaseregulator2	1	1
Ubiquitinfamily	3	3
Replicationfactor-ACterminaldomain	1	1
STI1domain	1	1
GlutathioneS-transferase,N-terminaldomain	17	17
Polysaccharidelyasefamily4,domainII	3	3
JAB1/Mov34/MPN/PAD-1ubiquitinprotease	3	3
UnstructuredregiononcNMP-bindingprotein	1	1
Ladomain	3	3
Ribosome60SbiogenesisN-terminal	1	1
BARdomain	7	7
NADbindingdomainof6-phosphogluconatedehydrogenase	1	1
PhosphatidylinositolglycanclassN(PIG-N)	1	1
MutLCterminaldimerisationdomain	1	1

Coiled-coildomaincontainingprotein(DUF2052)	1	1
Electrontransferflavoproteindomain	3	3
SWIRMdomain	3	3
MCMP-loopdomain	5	5
2'-5'RNAligasesuperfamily	1	1
Myb-likeDNA-bindingdomain	7	7
Anaphase-promotingcomplexsubunit4WD40domain	3	3
HEAT-likerepeat	5	5
Metallo-peptidasefamilyM12	1	1
ReplicationfactorRFC1Cterminaldomain	1	1
26SproteasomeregulatorysubunitRPN6N-terminaldomain	1	1
Lariatdebranchingenzyme,C-terminaldomain	1	1
Anaphase-promotingcomplex,cyclosome,subunit3	5	5
KinetochoreCENP-Cfungalhomologue,Mif2,N-terminal	1	1
FATdomain	3	3
RaffinosesynthaseorseedimbibitionproteinSip1	1	1
Insulinase(PeptidasefamilyM16)	1	1
Histidinoldehydrogenase	1	1
LNS2(Lipin/Ned1/Smp2)	1	1
tRNAsynthetasesclassII(D,KandN)	7	7
HelicaseconservedC-terminaldomain	1	1
Zinc-binding	25	25
Bap31/Bap29transmembraneregion	1	1
EZH2N-terminaldomain	1	1
Fumarylacetoacetate(FAA)hydrolasefamily	5	5
S4domain	5	5
Ubiquitin-likemodifier-activatingenzymeATG7N-terminus	1	1
Galactoseoxidase,centraldomain	3	3
PutativeIgdomain	1	1
PRP1splicingfactor,N-terminal	1	1

GatB/GatEcatalyticdomain	1	1
SNAREdomain	3	3
Tetrahydrofolatedehydrogenase/cyclohydrolase,NAD(P)-bindingdomain	1	1
HECT-domain(ubiquitin-transferase)	5	5
HydantoinaseB/oxoprolinase	1	1
eIF3subunit6Nterminaldomain	1	1
PeptidasefamilyM1domain	5	5
PXAdomain	5	5
Multiproteinbridgingfactor1	1	1
PHD/FYVE-zinc-fingerlikedomain	1	1
Formyltransferase	5	5
Cytokine-inducedanti-apoptosisinhibitor1,Fe-Sbiogenesis	1	1
Aft1HRRdomain	1	1
RibosomalproteinL23,N-terminaldomain	1	1
Neutral/alkalinenon-lysosomalceramidase,N-terminal	1	1
TupN-terminal	1	1
lactate/malatedehydrogenase,alpha/betaC-terminaldomain	3	3
Phosphoribosylglycinamidesynthetase,ATP-grasp(A)domain	1	1
PINITdomain	1	1
GPIethanolaminephosphatetransferasemembraneregion	1	1
POB3-likeN-terminalPHdomain	1	1
ATP-utilisingchromatinassemblyandremodellingN-terminal	1	1
MetallopeptidasefamilyM24	11	11
Dolichyl-phosphate-mannose-proteinmannosyltransferase	5	5
Prp18domain	1	1
PROCN(NUC071)domain	1	1
KetopantoatereductasePanE/ApbACterminal	5	5
RibosomalL30N-terminaldomain	1	1
Glutaminesynthetase,catalyticdomain	1	1

Brf1-likeTBP-bindingdomain	1	1
EamA-liketransporterfamily	1	1
Animalhaemperoxidase	3	3
TubulinbindingcofactorC	1	1
BPG-independentPGAMN-terminus(iPGM_N)	1	1
SEN1Nterminal	1	1
Pre-mRNAsplicingfactorPRP21likeprotein	1	1
FibronectintypeIIIdomain	3	3
3'-5'exonuclease	1	1
D-arabinono-1,4-lactoneoxidase	1	1
bZIPtranscriptionfactor	9	9
Septin	7	7
RibosomalfamilyS4e	1	1
Myosinhead(motordomain)	3	3
Tudordomain	3	3
LeucineRichrepeat	7	7
ProteasomeregulatorysubunitC-terminal	1	1
Beta-ketoacylsynthase,N-terminaldomain	1	1
Amidase	11	11
C-terminalregionofMMR_HSR1domain	1	1
CRM1Cterminal	1	1
ATP-graspdomain	1	1
Arginine-tRNA-proteintransferase,Cterminus	1	1
Histidinephosphatasesuperfamily(branch1)	7	7
Mitochondrialproteinup-regulatedduringmeiosis	1	1
Minichromosomelossprotein,Mcl1,middleregion	1	1
ATPcitratelyasecitrate-binding	1	1
Syntaxin-likeprotein	1	1
ALG11mannosyltransferaseN-terminus	1	1
Scd6-likeSmdomain	1	1

RibosomalproteinsL26eukaryotic,L24Parchaeal	1	1
Phosphoribosylsynthetase-associateddomain	1	1
DRGFamilyRegulatoryProteins,Tma46	1	1
Cobalamin-independentsynthase,Catalyticdomain	5	5
Acyl-CoAdehydrogenase,N-terminaldomain	1	1
Sec23/Sec24trunkdomain	3	3
Domainofunknownfunction(DUF1771)	1	1
SETdomain	9	11
YjeF-relatedproteinN-terminus	3	3
DNAmitochondrialpolymeraseexonucleasedomain	1	1
Glycosyltransferasefamily28N-terminaldomain	1	1
3'exoribonucleasefamily,domain1	11	11
KRI1-likefamily	1	1
Caspasedomain	3	3
Eukaryoticaspartylprotease	13	13
Nucleolarcomplex-associatedprotein	1	1
Torusdomain	1	1
RibosomalproteinS3,C-terminaldomain	1	1
DomainfoundinIF2B/IF5	1	1
THOcomplexsubunit1transcriptionelongationfactor	1	1
DKCLD(NUC011)domain	1	1
DOCKN-terminus	1	1
VHSdomain	3	3
ReplicationfactorCC-terminaldomain	3	3
ExosomecomponentEXOSC1/CSL4	1	1
MIF4Glike	1	1
NucleotideexchangefactorFes1	1	1
DomainofunknownfunctioninPX-proteins(DUF3818)	1	1
Responseregulatorreceiverdomain	3	3
BTB/POZdomain	13	13

ICP0-bindingdomainofUbiquitin-specificprotease7	1	1
PPP5TPRrepeatregion	1	1
MediatorcomplexsubunitMed5	1	1
Nuclearprotein96	1	1
shortchaindehydrogenase	63	64
Pyridoxine5'-phosphateoxidaseC-terminaldimerisationregion	1	1
Ssl1-like	1	1
Glyoxalase/Bleomycinresistanceprotein/Dioxygenasesuperfamily	3	3
Cyclicnucleotide-bindingdomain	3	3
TranscriptioninitiationfactorIIA,gammasubunit	1	1
MOZ/SASfamily	3	3
Voltagegatedchloridechannel	3	3
Fis1C-terminaltetratricopeptiderepeat	1	1
Ku70/Ku80beta-barreldomain	1	1
FattyacidsynthasetypeIhelicaldomain	1	1
FMN-dependentdehydrogenase	5	5
BRO1-likedomain	1	1
TUP1-likeenhancerofsplit	1	1
PP-loopfamily	1	1
Nucleotidyltransferase	5	5
SPOCdomain	1	1
WWdomain	7	7
NGP1NT(NUC091)domain	1	1
PDZ-likedomain	5	5
RPELrepeat	1	1
Nup93/Nic96	1	1
VTCdomain	3	3
Glutamineamidotransferasesclass-II	1	1
Sof1-likedomain	1	1
RNApolymeraseRpb3/RpoAinsertdomain	1	1

Ferredoxin-foldanticodonbindingdomain	1	1
Smg-4/UPF3family	1	1
ZPR1zinc-fingerdomain	1	1
Glycerophosphoryldiesterphosphodiesterasefamily	3	3
2OG-Fe(II)oxygenasesuperfamily	9	9
Enoyl-CoAhydratase/isomerase	19	19
Aluminiumactivatedmalatetransporter	1	1
Utp21specificWD40associatedputativedomain	1	1
Histidinephosphatasesuperfamily(branch2)	9	9
MOSCdomain	3	3
Blt1N-terminaldomain	1	1
Svf1-likeC-terminallipocalin-likedomain	1	1
Phospholipidmethyltransferase	3	3
Eukaryotictranslationinitiationfactor3subunitG	1	1
Peptidasedimerisationdomain	1	1
Putativethreonine/serineexporter	3	3
NAD-dependentglycerol-3-phosphatedehydrogenaseC-terminus	1	1
Thiaminpyrophosphokinase,catalyticdomain	1	1
Putativeserinedehydratasedomain	1	1
Pro-kumamolisin,activationdomain	3	3
Sir2family	9	9
Nitriteandsulphitereductase4Fe-4Sdomain	1	1
tRNAmethyltransferasecomplexGCD14subunit	1	1
MitochondrialproteinfromFMP27	1	1
AbscisicacidG-proteincoupledreceptor	1	1
PeptidaseM16inactivedomain	1	1
FACTcomplexsubunit(SPT16/CDC68)	1	1
ANTHdomain	1	1
S1RNAbindingdomain	1	1
GlucoseinhibiteddivisionproteinA	1	0

Tyrosyl-DNAphosphodiesterase	1	1
Glycosylhydrolasefamily63C-terminaldomain	3	3
Creatinase/ProlidaseN-terminaldomain	1	1
Proteinofunknownfunction(Ytp1)	3	3
AnticodonbindingdomainoftRNAs	1	1
WD40associatedregioninTFIIDsubunit,NTD2domain	1	1
Hsp90protein	1	1
Glucanosyltransferase	5	5
Ubiquitin-conjugatingenzyme	25	25
CoAbindingdomain	3	3
Radical_SAMC-terminaldomain	1	1
TaurinecatabolismdioxygenaseTauD,TfdAfamily	14	14
AlphagalactosidaseA	3	3
SGSdomain	1	1
5'-nucleotidase,C-terminaldomain	3	3
Acyltransferase	9	9
Glutamineamidotransferaseclass-I	5	5
Quinolinatephosphoribosyltransferase,C-terminaldomain	1	1
Nucleoporincomplexsubunit54	1	1
Haloaciddehalogenase-likehydrolase	19	19
Choline/ethanolaminekinase	3	3
ActivatorofHsp90ATPase,N-terminal	1	1
Ydr279pproteinfamily(RNaseH2complexcomponent)wHTHdomain	1	1
Tubulin-tyrosineligasefamily	3	3
Fusaricacidresistanceprotein-like	5	5
Las17-bindingproteinactinregulator	5	5
Phosphofructokinase	1	1
Pectatelyasesuperfamilyprotein	1	1
EB1-likeC-terminalmotif	1	1
Beta-lactamasesuperfamilydomain	3	3

DENNdomain-containingprotein11	1	1
DnaJdomain	19	19
Exportin-T	1	1
DockingdomainofAfi1forArf3invesicletrafficking	1	1
Sugar(andother)transporter	111	108
Cyclin,N-terminaldomain	3	3
RibosomalProteinsL2,C-terminaldomain	3	3
RNA-binding,Nab2-typezincfinger	1	1
CRAL/TRIOdomain	3	3
Thioredoxin-likedomain	1	1
Alphaamylase,catalyticdomain	3	3
Proteinphosphatase2C	9	9
CHORD	1	1
SHS2domainfoundinNterminusofRpb7p/Rpc25p/MJ0397	1	1
NPL4family	1	1
RNApolymeraseIIItranscriptionfactor(TF)IIICsubunitHTHdomain	1	1
Originrecognitioncomplex(ORC)subunit3N-terminus	1	1
Sec63Brldomain	1	1
SISdomain	1	1
Proteolysis_6C-terminal	1	1
Glutathione-dependentformaldehyde-activatingenzyme	13	13
Phosphoglucomutase/phosphomannomutase,C-terminaldomain	1	1
Lumazinebindingdomain	1	1
TFIIF,betasubunitN-terminus	1	1
6-phosphogluconatedehydrogenase,C-terminaldomain	1	1
Glycosylhydrolasesfamily15	1	1
tRNAsynthetasesclassIIcoredomain(F)	1	1
RNA3'-terminalphosphatecyclase	1	1
CAAXprenylproteaseN-terminal,fivemembranehelices	1	1
PITHdomain	1	1

ribosomalL5PfamilyC-terminus	1	1
PeptidaseM16Cassociated	1	1
Pyridoxal-dependentdecarboxylase,pyridoxalbindingdomain	1	1
Nucleoplasmin-likedomain	1	1
Proliferatingcellnuclearantigen,N-terminaldomain	1	1
Acetyl-CoAcarboxylase,centralregion	1	1
Mevalonate5-diphosphatedecarboxylaseC-terminaldomain	1	1
Phosphoadenosinephosphosulfatereductasefamily	3	3
Sortilin,neurotensinreceptor3,	1	1
Oligonucleotide/oligosaccharide-binding(OB)-fold	1	1
KilA-Ndomain	1	1
Fes/CIP4,andEFC/F-BARhomologydomain	1	1
GTPase-activatorproteinforRas-likeGTPase	3	3
IEC3subunitoftheIno80complex,chromatinre-modelling	1	1
Prolyl-tRNAsynthetase,C-terminal	1	1
Mono-functionalDNA-alkylatingmethylmethanesulfonateN-term	3	3
FissionyeastcentromereproteinN-terminaldomain	1	1
AN1-likeZincfinger	3	3
Regulated-SNARE-likedomain	1	1
Asparaginesynthase	3	3
Nuf2family	1	1
Ubiquitin-activatingenzymeactivesite	1	1
Glycosyltransferasefamily,a/bdomain	1	1
Familyofunknownfunction(DUF6314)	1	1
TranscriptionfactorTFIIBrepeat	1	1
Glycogendebranchingenzyme,glucanotransferasedomain	1	1
Pterinbindingenzyme	1	1
F-boxonlyproteinC-terminalregion	1	1
C-terminaldomainofSin3aprotein	1	1
16SrRNAmethyltransferaseRsmB/F	1	1

Dfp1/Him1,centralregion	1	1
Dimerisationandcyclophilin-bindingdomainofMon2	1	1
N-terminaldomainofribosephosphatepyrophosphokinase	1	1
SPFHdomain/Band7family	9	9
Homoserinedehydrogenase	1	1
Fructose-1-6-bisphosphatase,N-terminaldomain	1	1
Proteinofunknownfunction(DUF1604)	1	1
Hydroxymethylglutaryl-coenzymeAsynthaseCterminal	1	1
Peroxin13,N-terminalregion	1	1
Niemann-PickC1Nterminus	1	1
Respiratory-chainNADHdehydrogenase51Kdsubunit	1	1
Myo-inositol-1-phosphatesynthase	1	1
Glycosylhydrolasefamily47	9	9
GTPaseofunknownfunctionC-terminal	1	1
PhospholipaseDActivesitemotif	1	1
GammatubulincomplexcomponentC-terminal	1	1
Trehalase	3	3
PeptidasefamilyM20/M25/M40	1	1
EnolaseC-terminaldomain-like	3	3
OccludedRNA-recognitionmotif	1	1
Generalcontrolnonderepressible1(Gcn1)N-terminal	1	1
TranscriptionfactorTFIIDcomplexsubunit8C-term	1	1
Calponinhomology(CH)domain	1	1
CrcB-likeprotein,CamphorResistance(CrcB)	1	1
UncharacterizedproteinfamilyUPF0016	1	1
Uricase	1	1
Heavy-metal-associateddomain	3	3
PB1domain	1	1
SAMdomain(Sterilealphamotif)	7	7
Galactokinasegalactose-bindingsignature	1	1

X-domainofDnaJ-containing	1	1
Myristoyl-CoA:proteinN-myristoyltransferase,C-terminaldomain	1	1
TLD	3	3
Zincfinger,ZZtype	1	1
TLCdomain	3	3
Ribosomallargesubunitproteins60SL5,and50SL18	1	1
NIPSNAP	1	1
CobB/CobQ-likeglutamineamidotransferasedomain	1	1
Tetrapyrrole(Corrin/Porphyrin)Methylases	3	3
TAPC-terminaldomain	1	1
Acyl-CoAoxidase	1	1
Dualspecificityproteinphosphatase,N-terminalhalf	1	1
ATP-sulfurylase	1	1
CentrosominN-terminalmotif1	5	5
SDA1	1	1
Innermembranecomponentdomain	1	1
MolybdatetransporterofMFSsuperfamily	1	1
Domainofunknownfunction(DUF1977)	1	1
CorehistoneH2A/H2B/H3/H4	5	5
R3Hdomain	3	3
rRNA-processingarchdomain	1	1
DUF2407C-terminaldomain	1	1
Fourhelicalbundledomain	1	1
Domainofunknownfunction(DUF3336)	3	3
Ankyrinrepeats(manycopies)	5	5
PutativeNAD(P)-binding	1	1
Glycosyltransferasesgroup1	3	3
DNAtopoisomerase	3	3
NterminusofRad21/Rec8likeprotein	1	1
Biotin-requiringenzyme	1	1

CENP-BN-terminalDNA-bindingdomain	1	1
CTDkinasesubunitgammaCTK3	1	1
MidasinAAAliddomain	1	1
BP28CT(NUC211)domain	1	1
Hydroxyethylthiazolekinasefamily	1	1
RibosomalL28eproteinfamily	3	3
CLASPNterminal	1	1
Domainofunknownfunction(DUF4210)	1	1
PPRrepeat	5	5
RibosomalproteinL11,RNAbindingdomain	1	1
Squaleneepoxidase	1	1
Domainofunknownfunction(DUF3337)	1	1
EF-handdomain	7	7
IndigoidinesynthaseAlikeprotein	1	1
KinasebindingproteinCGI-121	1	1
Rit1N-terminaldomain	1	1
Beta-lactamaseassociatedwingedhelixdomain	1	1
Rpn9C-terminalhelix	1	1
SprT-likefamily	3	3
1,3-beta-glucansynthasecomponent	1	1
PASdomain	3	3
Glucosyltransferase24	1	1
CoatomerWDassociatedregion	1	1
DNAreplicationcomplexGINSproteinSLD5C-terminus	1	1
Gelsolinrepeat	1	1
Glycosyltransferasefamily20	1	1
C-terminusofhistoneH2A	1	1
SPXdomain	1	1
Vacuolarprotein14C-terminalFig4pbinding	1	1
MIF4Gdomain	1	1

Exportin-5family	1	1
Putativepeptidasefamily	1	1
HSF-typeDNA-binding	1	1
PAP2superfamily	9	9
RPAP1-like,C-terminal	1	1
pre-mRNAprocessingfactor4(PRP4)like	1	1
Glycosylhydrolasefamily115	1	1
SHR-bindingdomainofvacuolar-sortingassociatedprotein13	1	1
GC-richsequenceDNA-bindingfactor-likeprotein	1	1
Ubiquitinelongatingfactorcore	1	1
ShugoshinN-terminalcoiled-coilregion	1	1
Zinc-ribbon	3	3
Rasfamily	29	29
ABCtransportertransmembraneregion2	1	1
LeucineRichrepeats(2copies)	1	1
UbiquitinfusiondegradationproteinUFD1	1	1
Guaninenucleotideexchangefactorsynembryn	1	1
NucleoporinNup120/160	1	1
Histidyl-tRNAsynthetase	1	1
FYVEzincfinger	3	3
SBDSproteinC-terminaldomain	1	1
Serinecarboxypeptidase	7	7
MolybdenumCofactorSynthesisC	1	1
Skp1family,dimerisationdomain	1	1
Est1DNA/RNAbindingdomain	1	1
X-Prodipeptidyl-peptidaseC-terminalnon-catalyticdomain	1	1
MuniscinC-terminalmuhomologydomain	1	1
MitochondrialdegradasomeRNAhelicasesubunitCterminal	1	1
Gryzun,putativetraffickingthroughGolgi	1	1
Glycosylhydrolasefamily81C-terminaldomain	1	1

Chromoshadowdomain	1	1
HEATrepeats	3	3
Signalpeptidepeptidase	1	1
Signalrecognitionparticle,alphasubunit,N-terminal	1	1
DNApolymerasebetapalm	1	1
RhamnogalacturonanlyaseB,N-terminal	1	1
Tetrahydrofolatedehydrogenase/cyclohydrolase,catalyticdomain	1	1
EF-1guaninenucleotideexchangedomain	1	1
SisterchromatidcohesionC-terminus	1	1
2-oxoaciddehydrogenasesacyltransferase(catalyticdomain)	1	1
HSCBC-terminaloligomerisationdomain	1	1
Tuberculosisnecrotizingtoxin	1	1
AuxiliaryActivityfamily9(formerlyGH61)	7	7
PutativeSAM-dependentmethyltransferase	1	1
RibosomalproteinL7Ae/L30e/S12e/Gadd45family	9	9
CsbD-like	1	1
Aminotransferaseclass-V	7	7
LEM3(ligand-effectmodulator3)family/CDC50family	1	1
Carboxymuconolactonedecarboxylasefamily	1	1
Glycosylhydrolasesfamily17	5	5
Aldo/ketoreductasefamily	31	30
Maf1regulator	1	1
Mpp10protein	1	1
TypeI3-dehydroquinase	1	1
Peroxisomalmembraneprotein(Pex16)	1	1
Heterokaryonincompatibilityprotein(HET)	13	13
EpoxidehydrolaseNterminus	3	3
Uncharacterisedconservedprotein	1	1
Pal1cellmorphologyprotein	1	1
LMBR1-likemembraneprotein	3	3

Prolinedehydrogenase	3	3
Di-sulfidebridgenucleocytoplasmictransportdomain	1	1
TFIIShelicalbundle-likedomain	1	1
Thioesterase-likesuperfamily	7	7
Predictedintegralmembranezinc-ribbonmetal-bindingprotein	1	1
GRIPdomain	1	1
TCP-1/cpn60chaperoninfamily	16	15
EukaryotictranslationinitiationfactoreIF2A	1	1
Proteinofunknownfunction(DUF1691)	1	1
N-acetyltransferaseBcomplex(NatB)noncatalyticsubunit	1	1
Zinc-fingerdouble-strandedRNA-binding	5	5
Carboxylesterasefamily	7	7
Alpha/betahydrolasefamily	27	27
Phytanoyl-CoAdioxygenase(PhyH)	9	9
Methyltransferasedomain	47	47
Acetamidase/Formamidasefamily	1	1
GDA1/CD39(nucleosidephosphatase)family	1	1
Transmembraneaminoacidtransporterprotein	21	23
Adenosylmethioninedecarboxylase	1	1
NmrA-likefamily	35	37
ReplicationForkProtectionComponentSwi3	1	1
PRELI-likefamily	3	3
Bacterialtransferasehexapeptide(sixrepeats)	1	1
Lowmolecularweightphosphotyrosineproteinphosphatase	1	1
UcrQfamily	1	1
Proteinofunknownfunction(DUF1761)	1	1
Initiationfactor2subunitfamily	7	7
Acetyltransferase(GNAT)family	27	27
DNArepairproteinendonucleaseSAE2/CtIPC-terminus	1	1
Glycosylhydrolasecatalyticcore	3	3

Lyticpolysaccharidemono-oxygenase,cellulose-degrading	1	1
MeioticcellcortexC-terminalpleckstrinhomology	1	1
Opy2protein	1	1
Aldehydedehydrogenasefamily	34	34
HDdomain	11	11
Linalooldehydratase/isomerase	1	0
Tyrosinephosphatasefamily	5	5
Eukaryoticmitochondrialregulatorprotein	1	1
Carbon-nitrogenhydrolase	7	7
Lipase(class3)	11	11
Enoyl-(Acylcarrierprotein)reductase	52	53
Sulfotransferasedomain	1	1
Glycosylhydrolasesfamily11	3	3
N-Acetylglucosaminyltransferase-IV(GnT-IV)conservedregion	1	1
AcyclicterpeneutilisationfamilyproteinAtuA	3	3
ProteinofunknownfunctionDUF84	1	1
NAD(P)H-binding	15	15
PeptidaseS24-like	1	1
Spo12family	1	1
Mpv17/PMP22family	9	9
DSS1/SEM1family	1	1
Endonuclease/Exonuclease/phosphatasefamily	7	7
GPR1/FUN34/yaaHfamily	11	11
PXdomain	5	5
DJ-1/PfpIfamily	9	9
LSMdomain	17	17
Pan3Pseudokinasedomain	1	1
GATdomain	1	1
Fattyacidhydroxylasesuperfamily	13	13
ARP2/3complex20kDasubunit(ARPC4)	1	1

SerinecarboxypeptidaseS28	1	1
Peroxidase,family2	5	5
ClassIIAldolaseandAdducinN-terminaldomain	5	5
Autophagy-relatedprotein13	1	1
Cupindomain	11	11
Nucleartransportfactor2(NTF2)domain	1	1
Putativeadipose-regulatoryprotein(Seipin)	1	1
Phenazinebiosynthesis-likeprotein	1	1
IMP-specific5'-nucleotidase	1	1
MafB19-likedeaminase	3	3
Permeasefamily	3	3
AcylCoAbindingprotein	1	1
UreF	1	1
Shikimatekinase	3	3
Nitroreductasefamily	3	3
HPPfamily	1	1
Beta-lactamase	7	5
Glycosyltransferasesugar-bindingregioncontainingDXDmotif	5	5
YqcI/YcgGfamily	1	1
Cut12conserveddomain	1	1
Dihydrodipicolinatesynthetasefamily	11	11
Pre-mRNAsplicingPrp18-interactingfactor	1	1
RibosomalproteinL21e	1	1
Anp1	5	5
Zincfinger,C3HC4type(RINGfinger)	5	5
tRNAintronendonuclease,catalyticC-terminaldomain	3	3
PAXNEBprotein	1	1
RNApolymeraseRpb8	1	1
Proteinofunknownfunction(DUF775)	1	1
zinc-RINGfingerdomain	1	1

Pre-mRNA-splicingfactorofREScomplex	1	1
Anaphase-promotingcomplexsubunit5	1	1
ComplexIintermediate-associatedprotein30(CIA30)	1	1
Tim44-likedomain	1	1
Phosphoenolpyruvatephosphomutase	5	5
RNA-bindingsignalrecognitionparticle68	1	1
Hsp70protein	9	9
Uncharacterisedproteindomain(DUF2415)	1	1
Coiled-coildomain-containingprotein124/Oxs1	1	1
Transaldolase/Fructose-6-phosphatealdolase	3	3
NFACTproteinRNAbindingdomain	1	1
RING-variantdomain	3	3
tRNApseudouridinesynthase	3	3
RibosomalproteinS7e	1	1
MitochondrialATPsynthasegsubunit	1	1
SecE/Sec61-gammasubunitsofproteintranslocationcomplex	1	1
Patatin-likephospholipase	2	2
T5orf172domain	1	1
Arrestin(orS-antigen),N-terminaldomain	3	3
POTfamily	5	5
Double-strandedDNA-bindingdomain	1	1
Sas10/Utp3/C1Dfamily	3	3
Pregnancy-associatedplasmaprotein-A	1	1
MycotoxinbiosynthesisproteinUstYa	3	3
UBXdomain	3	3
MYNDfinger	3	3
SURF1family	1	1
haloaciddehalogenase-likehydrolase	7	7
Domainofunknownfunction(DUF4452)	1	1
Ham1family	1	1

PeptidasefamilyC78	1	1
Wingsapart-likeproteinregulationofheterochromatin	1	1
Uracilphosphoribosyltransferase	3	3
TranscriptionelongationfactorElf1like	1	1
RibosomalS3Aefamily	1	1
Actininteractingprotein3	1	1
Lysophospholipasecatalyticdomain	5	5
IKI3family	1	1
Acyl-coenzymeA:6-aminopenicillanicacidacyl-transferase	1	1
Proteinofunknownfunction(DUF3984)	1	1
Hptdomain	1	1
Grap2andcyclin-D-interacting	1	1
AKAP72'5'RNAligase-likedomain	1	1
Rtf2RING-finger	1	1
Probablemolybdopterinbindingdomain	1	1
NUBPLiron-transferP-loopNTPase	3	3
TranscriptionfactorS-II(TFIIS)	3	3
Folliculin-interactingproteinN-terminus	1	1
Sin3bindingregionofhistonedeacetylasecomplexsubunitSAP30	1	1
CaseinkinasesubstratephosphoproteinPP28	1	1
DNAprimasesmallsubunit	1	1
tRNA(Guanine-1)-methyltransferase	1	1
MitochondrialbiogenesisAIM24	1	1
Uncharacterisedproteinfamily(UPF0160)	1	1
cAMP-regulatedphosphoprotein/endosulfineconservedregion	1	1
Glutamate-cysteineligase	1	1
Familyofunknownfunction(DUF5308)	1	1
ArgonautesiRNAchaperone(ARC)complexsubunitArb1	1	1
Inositolpolyphosphatekinase	3	3
Proteolipidmembranepotentialmodulator	3	3

RibonucleaseIIIdomain	1	1
RTA1likeprotein	17	17
LURP-one-related	1	1
Dyp-typeperoxidasefamily	1	1
GRABdomain	1	1
CHD5-likeprotein	1	1
Transferasefamily	1	1
ProteintraffickingPGA2	1	1
Heatshockfactorbindingprotein1	1	1
RING-H2zincfingerdomain	1	1
HpcH/HpaIaldolase/citratelyasefamily	3	3
Bacterialextracellularsolute-bindingprotein	1	0
Peptidyl-tRNAhydrolasePTH2	1	1
Sodium/hydrogenexchangerfamily	7	7
Pectinesterase	1	1
Phosphatidylinositol-glycanbiosynthesisclassSprotein	1	1
Pyridoxal-dependentdecarboxylaseconserveddomain	11	11
F-boxdomain	1	1
RibosomebiogenesisproteinNop16	1	1
Gprotein-coupledglucosereceptorregulatingGpa2	3	3
Tannaseandferuloylesterase	3	3
CoA-transferasefamilyIII	5	5
2-phospho-L-lactatetransferaseCofD	1	1
ErgosterolbiosynthesisERG4/ERG24family	1	1
Mannose-6-phosphatereceptor	1	1
Acyltransferasefamily	3	3
Pectatelyase	21	21
EndoribonucleaseL-PSP	13	13
Glutathioneperoxidase	1	1
Peroxidase	9	9

PeptidasefamilyS58	1	1
Pyoverdine/dityrosinebiosynthesisprotein	3	3
Peroxin-3	1	1
Autophagy-relatedprotein101	1	1
Putativecyclase	1	1
EutQ-likecupindomain	1	1
L-rhamnosemutarotase	1	1
Etoposide-inducedprotein2.4(EI24)	3	3
S1/P1Nuclease	1	1
Arginasefamily	3	3
Aminotransferaseclass-III	11	11
Calpainfamilycysteineprotease	3	3
GlucosidaseIIbetasubunit-likeprotein	1	1
NADH:flavinoxidoreductase/NADHoxidasefamily	13	13
RibonucleaseT2family	1	1
Autophagy-relatedprotein27	1	1
NADH-ubiquinoneoxidoreductaseB18subunit(NDUFB7)	1	1
Carbohydratephosphorylase	1	1
G-proteinalphasubunit	1	1
FHAdomain	7	7
Respiratory-chainNADHdehydrogenase,30Kdsubunit	1	1
Phosphotyrosylphosphateactivator(PTPA)protein	3	3
GTPcyclohydrolaseII	1	1
Proteinofunknownfunction(DUF3591)	1	1
SRF-typetranscriptionfactor(DNA-bindinganddimerisationdomain)	1	1
Isocitrate/isopropylmalatedehydrogenase	9	9
Phosphoglucoseisomerase	1	1
MitochondrialribosomalsubunitS27	1	1
RibosomalproteinS24e	1	1
Lysinemethyltransferase	9	9

Fungalproteinofunknownfunction(DUF1774)	1	1
Putativeamidoligaseenzyme	3	3
PhosphomannoseisomerasetypeI	3	3
Trehaloseutilisation	1	1
SGF29tudor-likedomain	1	1
ADP-ribosylationfactorfamily	7	7
Amidohydrolase	25	25
Glycosylhydrolasesfamily28	5	5
GrpBprotein	1	1
Ppx/GppAphosphatasefamily	3	3
HomocysteineS-methyltransferase	1	1
Histone-liketranscriptionfactor(CBF/NF-Y)andarchaealhistone	7	7
Utp8family	1	1
Chitinsynthaseexportchaperone	1	1
Glycosylhydrolasesfamily16	15	15
Glycine-richdomain-containingprotein-like	5	5
Ribosomalsilencingfactorduringstarvation	1	1
PutativetRNAbindingdomain	1	1
VITfamily	1	1
NADdependentepimerase/dehydratasefamily	9	9
Ringhydroxylatingalphasubunit(catalyticdomain)	1	1
Isochorismatasefamily	1	1
GDSL-likeLipase/Acylhydrolasefamily	5	5
Cytidylyltransferase-like	7	7
EthanolamineutilisationproteinEutQ	1	1
Proteinofunknownfunction(DUF3445)	2	2
Phosphoesterasefamily	1	1
Chromatinassemblyfactor1subunitA	1	1
ubiE/COQ5methyltransferasefamily	3	3
Dienelactonehydrolasefamily	3	3

IGRproteinmotif	1	1
tRNAN6-adenosinethreonylcarbamoyltransferase	3	3
Enhancerofpolycomb-like	1	1
Polysaccharidedeacetylase	3	3
Phosphatidylinositol-specificphospholipaseC,Xdomain	3	3
Trypsin	1	1
Arrestin_Nterminallike	3	3
Luciferase-likemonooxygenase	1	1
Acetyltransferase(GNAT)domain	25	25
RadicalSAMsuperfamily	1	1
Proteinofunknownfunction(DUF1681)	1	1
Senescence-associatedprotein	1	1
GlycinecleavageH-protein	3	3
Serinehydroxymethyltransferase	3	3
SH3domain	5	5
Rtr1/RPAP2family	1	1
RibosomalS17	1	1
Fungalhydrophobin	1	1
Mitochondrialribosomalproteinsubunit	3	3
QueuinetRNA-ribosyltransferase	3	3
AminopeptidaseIzincmetalloprotease(M18)	3	3
Histonedeacetylasedomain	5	5
Membranedipeptidase(PeptidasefamilyM19)	5	5
3-betahydroxysteroiddehydrogenase/isomerasefamily	3	3
Beta-eliminatinglyase	5	5
Putativeheavy-metal-binding	1	1
ERproteinPkr1	1	1
Proteinofunknownfunction(DUF3808)	1	1
Xyloseisomerase-likeTIMbarrel	1	0
Fungaltrichotheceneeffluxpump(TRI12)	1	1

Proteinofunknownfunction(DUF1640)	3	3
GlutathioneS-transferase,C-terminaldomain	5	5
Polyketidecyclase/dehydraseandlipidtransport	3	3
60sAcidicribosomalprotein	1	1
SPT2chromatinprotein	1	1
EsterasePHBdepolymerase	3	3
IntegralmembraneproteinEMC3/TMCO1-like	1	1
Microsomalsignalpeptidase25kDasubunit(SPC25)	1	1
CFEMdomain	11	9
ATP11protein	1	1
Partialalpha/beta-hydrolaselipaseregion	1	1
Mycolicacidcyclopropanesynthetase	3	3
MaoClikedomain	3	3
TypeIphosphodiesterase/nucleotidepyrophosphatase	3	3
Prion-inhibitionandpropagation	1	1
Glycosyltransferasefamily8	3	3
Insulin-inducedprotein(INSIG)	1	1
Dephospho-CoAkinase	1	1
D-mannosebindinglectin	1	1
ERlumenproteinretainingreceptor	3	3
Tim17/Tim22/Tim23/Pmp24family	7	7
CSdomain	3	3
RibosomalproteinL3	11	11
LUC7N_terminus	1	1
Gpi16subunit,GPItransamidasecomponent	1	1
Proteinofunknownfunction(DUF2841)	1	1
Helix-loop-helixDNA-bindingdomain	17	17
NADH-ubiquinoneoxidoreductaseB12subunitfamily	1	1
Chitinsynthesisregulation,resistancetoCongored	3	3
PutativeS-adenosyl-L-methionine-dependentmethyltransferase	1	1

Proteinofunknownfunction(DUF726)	3	3
Vps51/Vps67	3	3
Antibioticbiosynthesismonooxygenase	1	1
A49-likeRNApolymeraseIassociatedfactor	1	1
Familyofunknownfunction(DUF5572)	1	1
Alg9-likemannosyltransferasefamily	5	5
GDSL-likeLipase/Acylhydrolase	9	7
Tim10/DDPfamilyzincfinger	5	5
Glycosylhydrolasefamily76	5	5
GPItransamidasesubunitPIG-U	1	1
RNApolymeraseIspecifictranscriptioninitiationfactor	3	3
GWT1	1	1
Glycosyltransferaselikefamily2	1	1
Clr5domain	7	7
Flavodoxin-likefold	1	1
Originrecognitioncomplexsubunit2	1	1
Phosphoribosyltransferasedomain	5	5
Mitochondrialdomainofunknownfunction(DUF1713)	1	1
Histoneacetyltransferasessubunit3	1	1
CybS,succinatedehydrogenasecytochromeBsmallsubunit	1	1
Importin-betaN-terminaldomain	3	3
Maf-likeprotein	1	1
Domainofunknownfunction(DUF3844)	1	1
Inositolphospholipidsynthesisandfat-storage-inducingTM	1	1
LITAF-likezincribbondomain	1	1
Rhodanese-likedomain	7	7
HemerythrinHHEcationbindingdomain	3	3
SKIP/SNWdomain	1	1
RNApolymeraseRpc34subunit	1	1
emp24/gp25L/p24family/GOLD	7	7

Proteinofunknownfunction(DUF1275)	1	1
IronpermeaseFTR1family	5	5
XLF-Cernunnos,XRcc4-likefactor,NHEJcomponent	1	1
RibosomalproteinS21e	1	1
Fic/DOCfamily	1	1
PhoD-likephosphatase	1	1
RibosomalproteinL14p/L23e	3	3
Familyofunknownfunction(DUF5427)	1	1
Sodium:solutesymporterfamily	3	1
Rrp15p	1	1
HMGL-like	1	1
Peptidemethioninesulfoxidereductase	1	1
Inhibitorofapoptosis-promotingBax1	3	3
Glycolipidtransferprotein(GLTP)	1	1
Inositolmonophosphatasefamily	5	5
Yip1domain	3	3
Glycosylhydrolasefamily45	1	0
Vps52/Sac2family	1	1
Rab-GTPase-TBCdomain	9	9
COMPASS(ComplexproteinsassociatedwithSet1p)componentshg1	1	1
RanBP1domain	1	1
RNApolymeraseIspecificinitiationfactor	1	1
ETCcomplexIsubunitconservedregion	1	1
Triose-phosphateTransporterfamily	9	9
Bin/amphiphysin/Rvsdomainforvesiculartrafficking	1	1
Arv1-likefamily	1	1
ChromosomesegregationproteinCsm1/Pcs1	1	1
PGAP1-likeprotein	1	1
Choline/Carnitineo-acyltransferase	3	3
pfkBfamilycarbohydratekinase	3	3

Exo70exocystcomplexsubunit	1	1
SecretorypathwayproteinSec39	1	1
Proteinofunknownfunction(DUF2838)	1	1
CytochromecoxidasesubunitVII	1	1
Clathrinlightchain	1	1
Serineaminopeptidase,S33	5	5
Proteinofunknownfunction(DUF3455)	1	1
MED6mediatorsubcomplexcomponent	1	1
RibosomalproteinL22p/L17e	1	1
Multiplemyelomatumor-associated	1	1
N2227-likeprotein	1	1
Proteasomeassemblychaperone4	1	1
Eukaryoticintegralmembraneprotein(DUF1751)	3	3
SPRYdomain	1	1
Proteinofunknownfunction(DUF3684)	1	1
DNA-directedRNApolymeraseIsubunitRPA34.5	1	1
DNApolymeraseIII,deltasubunit	1	1
RibosomalproteinL16p/L10e	3	3
Deoxyhypusinesynthase	1	1
ArgJfamily	1	1
BacteriallowtemperaturerequirementAprotein(LtrA)	5	5
GrpE	1	1
Imidazoleglycerol-phosphatedehydratase	1	1
Mob1/phoceinfamily	5	5
ATPsynthasedelta(OSCP)subunit	1	1
Putativeundecaprenyldiphosphatesynthase	1	1
InhibitorofgrowthproteinsN-terminalhistone-binding	3	3
TranscriptioninitiationfactorTFIIDcomponentTAF4family	1	1
Alanineracemase,N-terminaldomain	1	1
ARSbindingprotein2	1	1

Roothairdefective3GTP-bindingprotein(RHD3)	1	1
RibonucleaseHII	1	1
2EXRfamily	9	9
Rad51	3	3
PSP1C-terminalconservedregion	1	1
SUR7/PalIfamily	5	5
Ribosomerecyclingfactor	1	1
Up-regulatedDuringSeptation	1	1
Gamma-glutamylcyclotransferase,AIG2-like	5	5
RibosomalproteinL36	1	1
PLAC8family	3	3
TranscriptioninitiationfactorIID,31kDsubunit	1	1
UracilDNAglycosylasesuperfamily	3	3
Proteinofunknownfunction(DUF3752)	1	1
TEA/ATTSdomain	1	1
RNApolymerasesN/8kDasubunit	1	1
Proteinofunknownfunction(DUF1479)	1	1
EisosomecomponentPIL1	1	1
Cerato-platanin	5	5
RibosomalproteinS18	1	1
GPIbiosynthesisproteinfamilyPig-F	1	1
Cytidineanddeoxycytidylatedeaminasezinc-bindingregion	5	5
Thioredoxin-like[2Fe-2S]ferredoxin	0	0
Vacuolarsegregationsubunit7	1	1
T-complexprotein11	3	3
WKFdomain	1	1
Interferon-relateddevelopmentalregulator(IFRD)	1	1
Afadin-andalpha-actinin-Binding	1	1
IntegralmembraneproteinDUF92	1	1
Domainofunknownfunction(DUF4588)	1	1

Glycosyltransferasefamily90	1	1
NADHubiquinoneoxidoreductase,20Kdsubunit	1	1
Nup85Nucleoporin	1	1
Anaphase-promotingcomplexsubunit1	1	1
PeptidasefamilyM48	1	1
Cupinsuperfamily(DUF985)	1	1
TranscriptionalCoactivatorp15(PC4)	1	1
Ureidoglycolatelyase	1	1
Mitochondriallargesubunitribosomalprotein(Img2)	1	1
RibosomalproteinL44	1	1
U3smallnucleolarRNA-associatedprotein6	1	1
Cut8,nuclearproteasometetherprotein	1	1
DSBA-likethioredoxindomain	5	5
O-methyltransferase	9	9
MediatorofRNApolymeraseIItranscriptionsubunit1	1	1
PredictedSAM-dependentRNAmethyltransferase	1	1
TranscriptioninitiationfactorIID,18kDsubunit	1	1
Flavinatorofsuccinatedehydrogenase	1	1
U1zincfinger	1	1
CorA-likeMg2+transporterprotein	13	15
25SrRNA(adenine(2142)-N(1))-methyltransferase,Bmt2	1	1
Fungalproteinofunknownfunction(DUF2015)	1	1
Transportproteinparticle(TRAPP)component	5	5
Proteinofunknownfunction(DUF2423)	1	1
Nuclearcondensingcomplexsubunits,C-termdomain	1	1
MagnesiumtransporterNIPA	7	7
Translationallycontrolledtumourprotein	1	1
Mis12protein	1	1
Legume-likelectinfamily	1	1
Proteinofunknownfunction(DUF3767)	1	1

RibosomalproteinS11	3	3
Iron-sulphurclusterbiosynthesis	1	1
Ctrcoppertransporterfamily	3	3
RNApseudouridylatesynthase	1	1
NifU-likeNterminaldomain	1	1
HORMAdomain	3	3
Aldose1-epimerase	3	3
Subunit11ofthegeneraltranscriptionfactorTFIIH	1	1
PETassemblyofcytochromecoxidase,mitochondrial	1	1
Vacuolar(H+)-ATPaseGsubunit	1	1
Catenin-beta-like,Arm-motifcontainingnuclear	1	1
Uncharacterisedproteinfamily(UPF0203)	1	1
RNApolymeraseIItranscriptionmediatorcomplexsubunit9	1	1
Pyridoxamine5'-phosphateoxidase	7	7
TranscriptionmediatorcomplexsubunitMed12	1	1
RNApolymeraseIItranscriptionelongationfactor	1	1
N-terminalC2inEEIG1andEHBP1proteins	1	1
Translationinitiationfactor1A/IF-1	3	3
ERCC4domain	3	3
Ubiquitincarboxyl-terminalhydrolase,family1	5	5
Elongationcomplexprotein6	1	1
CBP4	1	1
Adenosine-deaminase(editase)domain	1	1
RibosomalproteinL24e	1	1
Redoxin	3	3
Fumble	1	1
Malatesynthase	1	1
CDP-alcoholphosphatidyltransferase	5	5
Copper/zincsuperoxidedismutase(SODC)	1	1
PeptidaseC1-likefamily	1	1

O-methyltransferasedomain	5	5
Mid2likecellwallstresssensor	1	1
UDP-glucoronosylandUDP-glucosyltransferase	1	1
ATPsynthase(C/AC39)subunit	1	1
Cnl2/NKP2familyprotein	1	1
RibosomalproteinS15	1	1
Uncharacterizedconservedprotein	7	7
Domainofunknownfunction(DUF1741)	1	1
RibosomalproteinL30p/L7e	1	1
6-O-methylguanineDNAmethyltransferase,DNAbindingdomain	1	1
Fructosaminekinase	1	1
Snf7	11	11
tRNA(Uracil-5-)-methyltransferase	1	1
Myosin-bindingstriatedmuscleassemblycentral	1	1
TranscriptionalrepressorTCF25	1	1
Flavincontainingamineoxidoreductase	7	7
CytochromecoxidaseassemblyproteinCtaG/Cox11	1	1
RNApolymeraseRpb4	3	3
GPI-GlcNActransferasecomplex,PIG-Hcomponent	1	1
DNApolymerasedelta,subunit4	1	1
PotentialQueuosine,Q,salvageproteinfamily	1	1
Majorintrinsicprotein	1	1
Uncharacterizedproteinconservedinbacteria(DUF2263)	1	1
cwf21domain	1	1
PalH/RIM21	1	1
Cwf15/Cwc15cellcyclecontrolprotein	1	1
Familyofunknownfunction(DUF5321)	1	1
PeptidasefamilyC50	1	1
Proteinofunknownfunction(DUF3605)	3	3
SRP-independenttargetingprotein2/TMEM208	1	1

RibosomalproteinS10p/S20e	1	1
MitochondrialribosomalproteinL31	1	1
Prephenatedehydratase	1	1
Sulfotransferasefamily	1	1
Transcriptionalregulationofmitochondrialrecombination	1	1
PIG-X/PBN1	1	1
GDP-mannose4,6dehydratase	1	1
Brixdomain	9	9
RNasePsubunitPop3	1	1
DehydrogenaseE1component	3	3
Neurochondrin	1	1
RibosomalproteinS7p/S5e	1	1
Gtr1/RagAGproteinconservedregion	3	3
Hus1-likeprotein	1	1
eIF-6family	1	1
Necrosisinducingprotein(NPP1)	1	1
Growth-Arrest-SpecificProtein2Domain	1	1
50Sribosome-bindingGTPase	5	5
Lowtemperatureviabilityprotein	1	1
SAC3/GANPfamily	1	1
ERG2andSigma1receptorlikeprotein	1	1
Nuclearporecomplexcomponent	1	1
GolgitoERtrafficprotein4	1	1
Uncharacterisedproteinfamily(UPF0220)	1	1
GDP/GTPexchangefactorSec2p	1	1
Eukaryoticporin	1	1
Smallsubunitofserinepalmitoyltransferase-like	1	1
RAI1likePD-(D/E)XKnuclease	1	1
CytochromecoxidasesubunitVa	1	1
GNS1/SUR4family	3	3

Cgr1family	1	1
Questinoxidase-like	1	1
Nucleosomeassemblyprotein(NAP)	3	3
LCCLdomain	1	1
Phospholipase/Carboxylesterase	3	3
Glycolipid2-alpha-mannosyltransferase	3	3
NuclearproteinEs2	1	1
Clathrinadaptorcomplexsmallchain	5	5
DomainofKin17curvedDNA-bindingprotein	1	1
Saf4/Yju2protein	3	3
rRNAbiogenesisproteinRRP36	1	1
RibosomalproteinS9/S16	3	3
VariantSH3domain	1	1
Pyridoxamine5'-phosphateoxidaselike	1	1
Extracellularmutantprotein11	1	1
Large-conductancemechanosensitivechannel,MscL	1	1
Leucine-richrepeat	1	1
TatDrelatedDNase	5	5
Non-classicalexportprotein1	1	1
Spc24subunitofNdc80	1	1
Ferrochelatase	1	1
Pathogeneffector putativenecrosis-inducingfactor	1	1
Nucleotide-sugartransporter	3	3
Lateendosomal/lysosomaladaptorandMAPKandMTORactivator	1	1
MED7protein	1	1
L-lysine6-monooxygenase(NADPH-requiring)	1	1
RibosomalproteinS8	7	7
Cornichonprotein	1	1
NADPH-dependentFMNreductase	3	3
FKBP-typepeptidyl-prolylcis-transisomerase	1	1

Eukaryoticcytochromeb561	1	1
Proteinofunknownfunction(DUF2012)	1	1
Cys/MetmetabolismPLP-dependentenzyme	5	7
Membrane-associatingdomain	7	7
Citratesynthase,C-terminaldomain	3	3
Domainofunknownfunction(DUF3425)	5	5
Apc15pprotein	1	1
Bacteriorhodopsin-likeprotein	1	1
Proteinofunknownfunction(DUF1168)	1	1
Calcipressin	1	1
Neugrin	1	1
Met-10+like-protein	3	3
Originrecognitioncomplexsubunit6(ORC6)	1	1
RNAsePRpr2/Rpp21/SNM1subunitdomain	1	1
tRNA-splicingendonucleasesubunitsen54N-term	1	1
RibosomalproteinL31e	1	1
RibDC-terminaldomain	1	1
Regulatorofvolumedecreaseaftercellularswelling	1	1
NOT2/NOT3/NOT5family	1	1
CUEdomain	5	5
RegulatorofVps4activityintheMVBpathway	1	1
COPIassociatedprotein	1	1
IBRdomain,ahalfRING-fingerdomain	3	3
Lactonase,7-bladedbeta-propeller	5	5
Cysteine-richTMmodulestresstolerance	1	1
Phytase	1	1
ChrRCupin-likedomain	1	1
Shortcoiled-coilprotein	1	1
Traffickingproteinparticlecomplexsubunit10,TRAPPC10	1	1
Mechanosensitiveionchannel	1	1

SNOglutamineamidotransferasefamily	1	1
Domainofunknownfunction(DUF2828)	1	1
Chromo(CHRromatinOrganisationMOdifier)domain	1	1
HypotheticalproteinFLILHELTA	1	1
Amino-transferaseclassIV	5	5
SnareregionanchoredinthevesiclemembraneC-terminus	3	3
RibosomalRNAadeninedimethylase	3	3
Putativezincfingermotif,C2HC5-type	1	1
Nop53(60Sribosomalbiogenesis)	1	1
Ribose5-phosphateisomeraseA(phosphoriboisomeraseA)	1	1
InositolphosphorylceramidesynthasesubunitKei1	1	1
CatalyticLigBsubunitofaromaticring-openingdioxygenase	3	3
Mitochondrialdistributionandmorphologyprotein10	1	1
tRNAsynthetasesclassI(C)catalyticdomain	1	1
RNApolymeraseI-associatedfactorPAF67	1	1
HistonemethylationproteinDOT1	1	1
Autophagyprotein16(ATG16)	1	1
Microtubuleassociatedprotein(MAP65/ASE1family)	1	1
TypeIICAAXprenylendopeptidaseRce1-like	1	1
Maintenanceofmitochondrialmorphologyprotein1	3	3
Domainofunknownfunction(DUF1772)	3	3
Microsomalsignalpeptidase12kDasubunit(SPC12)	1	1
MBOAT,membrane-boundO-acyltransferasefamily	3	3
Guanylatekinase	1	1
PeptidasefamilyS41	1	1
Promethin	1	1
Glycosyltransferasefamily21	1	1
Retinoicacidinduced16-likeprotein	1	1
Dehydratasefamily	3	3
Thioesterasedomain	1	1

Damage-controlphosphataseARMT1-likedomain	1	1
Glycosylhydrolasesfamily32N-terminaldomain	3	3
Peptidyl-tRNAhydrolase	3	3
Chalconeisomeraselike	1	1
RibosomalproteinL13	3	3
PLD-likedomain	3	3
MembraneboundO-acyltransferasefamily	1	1
SAPdomain	1	1
Hamartinprotein	1	1
CHCHdomain	1	1
PHD-finger	5	5
SCO1/SenC	1	1
Complex1_LYR-like	5	5
Spermine/spermidinesynthasedomain	1	1
Primasezincfinger	1	1
DASHcomplexsubunitDuo1	1	1
ESSSsubunitofNADH:ubiquinoneoxidoreductase(complexI)	1	1
Alteredinheritanceofmitochondriaprotein21	1	1
UncharacterizedconservedproteinCG6151-P	1	1
SelRdomain	1	1
ARID/BRIGHTDNAbindingdomain	1	1
DiaphanousGTPase-bindingDomain	1	1
Platelet-activatingfactoracetylhydrolase,isoformII	1	1
Uncharacterizedconservedprotein(DUF2183)	1	1
cAMPphosphodiesterasesclass-II	1	1
Flavinreductaselikedomain	1	1
Sideroflexins	1	1
Domainofunknownfunction(DUF4045)	1	1
SMP-30/Gluconolactonase/LRE-likeregion	3	3
Whi5like	3	3

rRNAprocessing/ribosomebiogenesis	1	1
ER-boundoxygenasempaB/B'/Rubberoxygenase,catalyticdomain	1	1
TranscriptionfactorsubunitMed10ofMediatorcomplex	1	1
RNApolymeraseRpb6	1	1
NRDE-2,necessaryforRNAinterference	1	1
Urb2/Npa2family	1	1
Domainofunknownfunction(DUF543)	1	1
Triosephosphateisomerase	1	1
MitochondrialribosomalproteinL51/S25/CI-B8domain	3	3
CaseinkinaseIIregulatorysubunit	3	3
RIC1	1	1
VMA21-likedomain	1	1
Opticatrophy3protein(OPA3)	1	1
Shwachman-Bodian-Diamondsyndrome(SBDS)protein	1	1
Dynamitin	1	1
RibosomalproteinS28e	1	1
Domainofunknownfunction(DUF4748)	1	1
TUGubiquitin-likedomain	1	1
Possiblelysinedecarboxylase	1	1
F-actincappingprotein,betasubunit	1	1
VacuoleeffluxerAtg22like	3	3
Proteinofunknownfunction(DUF3602)	7	7
PreproteintranslocasesubunitSec66	1	1
Regionofunknownfunction(DUF2417)	1	1
HMG(highmobilitygroup)box	3	3
Magobinding	1	1
Per1-likefamily	1	1
RibosomalprokaryoticL21protein	1	1
RibosomalproteinS8e	3	3
RWDdomain	1	1

Uncharacterizedalpha/betahydrolasedomain(DUF2235)	3	3
vonWillebrandfactortypeAdomain	1	1
Nbl1/BorealinNterminal	1	1
RNApolymeraseIItranscriptionfactorSIII(Elongin)subunitA	1	1
Fungaldomainofunknownfunction(DUF1750)	1	1
Cleftlipandpalatetransmembraneprotein1(CLPTM1)	1	1
Myosin-likecoiled-coilprotein	1	1
Domainofunknownfunction(DUF1993)	5	5
Haemolysin-IIIrelated	1	1
Fms-interactingprotein/Thoc5	1	1
Glycosyltransferasefamilygroup2	7	7
dUTPase	1	1
Mitochondrialcalciumuniporter	1	1
Cytochromec/c1hemelyase	1	1
CLN3protein	1	1
Nucleotide-diphospho-sugartransferase	1	1
Inorganicpyrophosphatase	1	1
Phosphatetransporterfamily	3	3
Familyofunknownfunction(DUF6454)	1	1
Murligasemiddledomain	1	1
ICE2	1	1
MSP(Majorspermprotein)domain	1	1
Proteinofunknownfunction(DUF2408)	1	1
YCII-relateddomain	1	1
3-demethylubiquinone-93-methyltransferase	1	1
MitochondrialribosomalproteinsubunitL20	1	1
Sec1family	3	3
CCCH-typezincfinger	1	1
Translinfamily	3	3
Gar1/Naf1RNAbindingregion	1	1

GINScomplexprotein	1	1
Rieske[2Fe-2S]domain	0	0
R3H-associatedN-terminaldomain	1	1
ATPsynthasesubunitK	1	1
Mitochondrial18KDaprotein(MTP18)	1	1
Permeaseforcytosine/purines,uracil,thiamine,allantoin	5	5
Glutamineamidotransferasedomain	1	1
ALG3protein	1	1
She9/Mdm33family	1	1
RibosomalproteinS12/S23	3	3
TopoisomeraseII-associatedproteinPAT1	1	1
RPC5protein	1	1
Endomembraneprotein70	1	1
Vitamin-D-receptorinteractingMediatorsubunit4	1	1
U-boxdomain	1	1
Dihydroorotatedehydrogenase	1	1
Thioesterasesuperfamily	5	5
Diacylglycerolkinasecatalyticdomain	3	3
TIM21	1	1
FRG1-likedomain	1	1
Iron-sulfurclusterassemblyprotein	1	1
ElongatorsubunitIki1	1	1
Ubiquitin-likeautophagyproteinApg12	1	1
Ketopantoatehydroxymethyltransferase	1	1
FtsJ-likemethyltransferase	1	1
Flavoprotein	5	5
Poly(ADP-ribose)glycohydrolase(PARG)	1	1
Domainofunknownfunction(DUF4078)	1	1
Peroxisomalbiogenesisfactor11(PEX11)	1	1
Celldifferentiationfamily,Rcd1-like	1	1

Pex14N-terminaldomain	1	1
HEC/Ndc80pfamily	1	1
BindingdomainofDNArepairproteinErcc1(rad10/Swi10)	1	1
Iron-containingalcoholdehydrogenase	3	3
Asxhomologydomain	1	1
Proteinofunknownfunction(DUF3405)	5	5
Pex19proteinfamily	1	1
Uncharacterizedproteinconservedinbacteria(DUF2264)	3	3
Plus-3domain	1	1
TranscriptionalregulatorofRNApolII,SAGA,subunit	1	1
Breastcarcinomaamplifiedsequence2(BCAS2)	1	1
Hyaluronan/mRNAbindingfamily	1	1
Isocitratelyasefamily	3	3
Yippeezinc-binding/DNA-binding/Mis18,centromereassembly	3	3
DNLzincfinger	1	1
Anion-transportingATPase	1	1
HIUase/Transthyretinfamily	1	1
RING-typezinc-finger	1	1
Glycoside-hydrolasefamilyGH114	5	5
MitochondrialexportproteinSom1	1	1
OST3/OST6family,transporterfamily	1	1
DNAmismatchrepairproteinHSM3,Nterminaldomain	1	1
DNA-directedRNApolymeraseIIIsubunitRpc31	1	1
Fip1motif	1	1
Alkalinephosphatase	1	1
Proteinofunknownfunction(DUF498/DUF598)	1	1
RibosomalproteinL34e	1	1
RibosomalproteinL32	1	1
AAR2protein	1	1
EAP30/Vps36family	1	1

MitochondrialimportreceptorsubunitTom22	1	1
YEATSfamily	3	3
Phosducin	3	3
AminotransferaseclassIandII	19	17
CytochromeC1family	1	1
GlcNAc-PIde-N-acetylase	1	1
Ceramidase	1	1
Squalene/phytoenesynthase	3	3
Mannosyltransferase(PIG-M)	1	1
Dyneinlightintermediatechain(DLIC)	1	1
Proteinofunknownfunction,DUF255	1	1
Proteintyrosineandserine/threoninekinase	1	1
Somethingaboutsilencing,SAS,complexsubunit4	1	1
CytochromeCoxidasecopperchaperone(COX17)	1	1
OligosaccharyltransferasesubunitRibophorinII	1	1
Hypoxiainducedproteinconservedregion	1	1
Proteinofunknownfunction(DUF3237)	1	1
Rpp20subunitofnuclearRNaseMRPandP	1	1
Serinehydrolase(FSH1)	3	3
Spc19	1	1
Dynactinsubunitp22	1	1
RibosomalproteinS16	1	1
PutativeRNAmethyltransferase	1	1
YeastcorticalproteinKAR9	1	1
SAICARsynthetase	1	1
TransportproteinTrs120orTRAPPC9,TRAPPIIcomplexsubunit	1	1
Dopa4,5-dioxygenasefamily	1	1
SURF4family	1	1
SWIMzincfinger	1	1
Nuclearporecomplexassembly	1	1

Polyprenylsynthetase	5	5
Alaninedehydrogenase/PNT,N-terminaldomain	1	1
Fringe-like	1	1
AmmoniumTransporterFamily	3	3
CRT10	1	1
ARP2/3complexARPC3(21kDa)subunit	1	1
7transmembranereceptor(Secretinfamily)	1	1
LicDfamily	1	1
3-oxo-5-alpha-steroid4-dehydrogenase	3	1
MINDYdeubiquitinase	1	0
RibosomalproteinS19	1	1
TranscriptioninitiationfactorTFIID23-30kDasubunit	1	1
Dihydroneopterinaldolase	1	1
OligosaccharidebiosynthesisproteinAlg14like	1	1
UTP--glucose-1-phosphateuridylyltransferase	1	1
XPAproteinC-terminus	1	1
Phosphatetransport(Pho88)	1	1
Ser-Thr-richglycosyl-phosphatidyl-inositol-anchoredmembranefamily	3	3
Polycystincationchannel	1	1
Macrodomain	1	1
RibonucleaseP/MRP,subunitp29	1	1
Pantoate-beta-alanineligase	1	1
Leo1-likeprotein	1	1
Alcoholacetyltransferase	3	3
PutativeFMN-bindingdomain	1	1
XAP5,circadianclockregulator	1	1
Alternariaalternataallergen1	1	1
KinetochorecomplexSim4subunitFta1	1	1
Conidiationprotein6	1	1
Fungalchitosanaseofglycosylhydrolasegroup75	1	1

Vacuolarproteinsorting55	1	1
Exonuclease	11	11
Metal-independentalpha-mannosidase(GH125)	1	1
DNApolymerasesubunitCdc27	1	1
Thocomplexsubunit7	1	1
Nnf1	1	1
PutativeSin3bindingprotein	1	1
Proteinofunknownfunction(DUF1295)	1	1
Domainofunknownfunction(DUF4149)	1	1
Dolicholphosphate-mannosebiosynthesisregulatoryprotein(DPM2)	1	1
Dihydrouridinesynthase(Dus)	1	1
Leucinerichrepeat	3	3
Rdxfamily	1	1
GGLdomain	1	1
Zinc-fingerofmitochondrialsplicingsuppressor51	1	1
UBA-likedomain	1	1
Coiled-coildomain-containingprotein55(DUF2040)	1	1
HistoneacetyltransferaseHAT1N-terminus	1	1
GDPdissociationinhibitor	3	3
Mitochondrial39-SribosomalproteinL47(MRP-L47)	1	1
TranslocationproteinSec62	1	1
Peptidaseofplantsandbacteria	1	1
RibophorinI	3	3
Yqey-likeprotein	1	1
Krr1KH1domain	1	1
Chaperonin10Kdsubunit	1	1
ComponentofIISlongevitypathwaySMK-1	1	1
Glycosyltransferasefamily2	9	11
RibosomalproteinL23	3	3
39SribosomalproteinL53/MRP-L53	1	1

RNApolIIaccessoryfactor,Cdc73family,C-terminal	1	1
Bystin	1	1
Sec20	1	1
PeptidasefamilyM3	3	3
Proteinofunknownfunction(DUF1304)	1	1
Ribosomalsubunit39S	1	1
DAHPsynthetaseIfamily	1	1
MAGEfamily	1	1
Splicingfactor3Bsubunit10(SF3b10)	1	1
ATP12chaperoneprotein	1	1
Inheritanceofperoxisomesprotein1	1	1
OTU-likecysteineprotease	1	1
Ribosome-assemblyprotein3	1	1
Gaa1-like,GPItransamidasecomponent	1	1
DASHcomplexsubunitDam1	1	1
Ran-interactingMog1protein	1	1
CSN8/PSMD8/EIF3Kfamily	3	3
Golgitransportcomplexsubunit5	1	1
Proteinofunknownfunction(DUF2434)	1	1
FAR-17a/AIG1-likeprotein	1	1
PeptidasefamilyC54	1	1
RibosomalproteinS17	1	1
Signalrecognitionparticle14kDprotein	1	1
Irononlyhydrogenaselargesubunit,C-terminaldomain	1	1
CHRDdomain	1	1
COQ9	1	1
THUMPdomain	1	1
Erv1/Alrfamily	1	1
SUZdomain	1	1
Uroporphyrinogen-IIIsynthaseHemD	1	1

Domainofunknownfunction(DUF1917)	1	1
UncharacterisedACR,YggUfamilyCOG1872	1	1
HADdomainfamily1inSwissArmyKnifeRNArepairproteins	1	1
Inosine-uridinepreferringnucleosidehydrolase	1	1
Proteinofunknownfunction(DUF2985)	3	3
Proteinofunknownfunction(DUF3429)	1	1
SIT4phosphatase-associatedprotein	1	1
Rhomboidfamily	1	1
Eukaryoticproteinofunknownfunction(DUF829)	3	1
Ubiquitin-2likeRad60SUMO-like	3	3
Proteinofunknownfunction(DUF3292)	1	1
Med18protein	1	1
Uncharacterizedconservedprotein(DUF2293)	1	1
NUC173domain	1	1
Calcineurin-likephosphoesterasesuperfamilydomain	1	1
DASHcomplexsubunitDad2	1	1
Cytochromeoxidasecomplexassemblyprotein1	1	1
ChromosomesegregationproteinSpc25	1	1
Mitotic-spindleorganizinggamma-tubulinringassociated	1	1
Plasma-membranecholinetransporter	3	3
Sec61betafamily	1	1
Ribosomalproteins50S-L18Ae/60S-L20/60S-L18A	1	1
BRCA1CTerminus(BRCT)domain	1	1
Rap1-interactingfactor1Nterminal	1	1
Class-IIDAHPsynthetasefamily	1	1
HYPKUBAdomain	1	1
RNasePsubunitp30	1	1
UncharacterisedACR,YagEfamilyCOG1723	3	3
ATPsynthase	25	25
galactosyltransferaseGMA12/MNN10family	1	1

MFS/sugartransportprotein	1	1
MoaCfamily	1	1
Pet100	1	1
Nucleotidehydrolase	1	1
UncharacterisedproteinfamilyUPF0047	1	1
Zinc-fingerdomain	3	3
3'5'-cyclicnucleotidephosphodiesterase	1	1
RibosomalproteinL1p/L10efamily	3	3
WSTF,HB1,Itc1p,MBD9motif1	1	1
G10protein	1	1
Eukaryoticproteinofunknownfunction(DUF846)	1	1
Fcf1	1	1
Transmembraneprotein33/NucleoporinPOM33	1	1
Palmitoylproteinthioesterase	1	1
CVNHdomain	1	1
Ferritin-likedomain	3	3
Mitoticcheckpointprotein	1	1
OligosaccharyltransferaseSTT3subunit	1	1
ECL1/2/3zincbindingproteins	1	1
Dualspecificityphosphatase,catalyticdomain	3	3
Homeodomain	3	3
Alphaandgammaadaptinbindingproteinp34	1	1
Omp85superfamilydomain	1	1
DNA/pantothenatemetabolismflavoprotein	1	1
WWdomainbindingprotein11	1	1
Glycosylhydrolasesfamily31	1	1
Pre-rRNA-processingproteinTSR2	1	1
BSDdomain	1	1
Glucose-repressibleproteinGrg1	1	1
Invertebrate-AID/APOBEC-deaminase	1	1

Cytochromeoxidaseassemblyprotein	1	1
Sucrase/ferredoxin-like	1	1
4'-phosphopantetheinyltransferasesuperfamily	1	1
YIF1	1	1
ConservedhypotheticalATPbindingprotein	3	3
Nuclearporeassemblyandbiogenesis	1	1
Proteinofunknownfunction(DUF3074)	1	1
TAFII55proteinconservedregion	1	1
Serine-threonineproteinkinase19	1	1
ProteasomecomplexsubunitRpn13ubiquitinreceptor	1	1
RibosomalproteinL6e	1	1
ATP10protein	1	1
RibosomalL29protein	1	1
HCNGP-likeprotein	1	1
Proteinofunknownfunction(DUF3712)	3	3
TspO/MBRfamily	1	1
Thymidylatesynthase	1	1
ERmembraneproteinSH3	1	1
RED-likeproteinN-terminalregion	1	1
Subunit17ofMediatorcomplex	1	1
MethyltransferaseTYW3	1	1
ProteinofUnknownfunction(DUF1690)	1	1
Carboxyltransferasedomain	1	1
PeptidaseinhibitorI78family	1	1
TranslationmachineryassociatedTMA7	1	1
DNAdirectedRNApolymerase,7kDasubunit	1	1
HITdomain	3	3
ChAPs(Chs5p-Arf1p-bindingproteins)	1	1
Symplekin/PTA1N-terminal	1	1
Coiled-coildomain-containingprotein56	1	1

Sds3-like	1	1
Prolineracemase	3	3
DNAdouble-strandbreakrepairandV(D)JrecombinationproteinXRCC4	1	1
WLMdomain	1	1
Glycosylhydrolasefamily1	7	7
NMT1/THI5like	1	1
Vacuolarimportanddegradationprotein	1	1
Magonashiprotein	1	1
CENP-Sprotein	1	1
PPPDEputativepeptidasedomain	1	1
NADARdomain	1	1
Vacuolarproteinsorting-associatedprotein35	1	1
Myo-inositoloxygenase	1	1
Fungalproteinofunknownfunction(DUF2011)	1	1
Cysteine-richsecretoryproteinfamily	1	1
Arrestin(orS-antigen),C-terminaldomain	1	1
NDT80/PhoGlikeDNA-bindingfamily	1	1
MitochondrialATPaseexpression	1	1
Alginatelyase	1	1
ElectrontransferDM13	1	1
EXPERA(EXPandedEBPsuperfamily)	1	1
SnoaL-likepolyketidecyclase	1	1
Universalstressproteinfamily	1	1
Surpmodule	1	1
Nitronatemonooxygenase	7	7
3,4-dihydroxy-2-butanone4-phosphatesynthase	1	1
Eukaryoticinitiationfactor4E	3	3
Zinc-fingeroftheMIZtypeinNsesubunit	1	1
Spinocerebellarataxiatype10proteindomain	1	1
Phosphorylasesuperfamily	1	1

TransportandGolgiorganisation2	1	1
Deuterolysinmetalloprotease(M35)family	1	1
Dolichol-phosphatemannosyltransferasesubunit3(DPM3)	1	1
Oxidoreductase-likeprotein,N-terminal	1	1
STAGdomain	1	1
UreDureaseaccessoryprotein	1	1
HNHendonuclease	1	1
TransmembraneadaptorErv26	1	1
Methylenetetrahydrofolatereductase	3	3
Proteasomestabiliser	1	1
56kDaseleniumbindingprotein(SBP56)	1	1
NADH-ubiquinoneoxidoreductaseASHIsubunit(CI-ASHIorNDUFB8)	1	1
Yeastmitochondrialdistributionandmorphology(MDM)proteins	1	1
TranslationinitiationfactoreIF3subunit	1	1
Alternativeoxidase	1	1
CBF/Mak21family	3	3
GRFzincfinger	1	1
Adenylylsulphatekinase	1	1
V-ATPasesubunitC	1	1
Modifierofrudimentary(Mod(r))protein	1	1
Nuclearporeprotein84/107	1	1
NitrogenPermeaseregulatorofaminoacidtransportactivity3	1	1
Rgp1	1	1
Fungalfamilyofunknownfunction(DUF1776)	1	1
Eukaryoticphosphomannomutase	1	1
Ykl077w/Psg1(Pma1StabilizationinGolgi)	1	1
JmjCdomain,hydroxylase	1	1
Complex1protein(LYRfamily)	1	1
Anaphase-promotingcomplexAPCsubunitCDC26	1	1
BolA-likeprotein	5	5

VPS28protein	1	1
Noc2pfamily	1	1
NAD(P)-bindingRossmann-likedomain	1	1
3-octaprenyl-4-hydroxybenzoatecarboxy-lyase	1	1
RNAcapguanine-N2methyltransferase	1	1
Metallo-beta-lactamasesuperfamily	5	5
Sulphurtransport	1	1
GRASP55/65PDZ-likedomain	1	1
AutophagyproteinApg5	1	1
LETM1-likeprotein	1	1
SCP-2steroltransferfamily	1	1
cwf18pre-mRNAsplicingfactor	1	1
CytochromecoxidasebiogenesisproteinCmc1like	1	1
RibosomalproteinL17	1	1
Delta-aminolevulinicaciddehydratase	1	1
Proteinofunknownfunction(DUF1279)	1	1
Mitochondrialimport2	1	1
ExosomecomplexexonucleaseRRP4N-terminalregion	1	1
Mitochondrialribosomalsubunitprotein	1	1
Domainofunknownfunction(DUF202)	1	1
RNApolymeraseIspecifictranscriptioninitiationfactorRRN3	1	1
Tctex-1family	1	1
Zinccarboxypeptidase	5	5
Dopey,N-terminal	1	1
ribonuclease	29	29
RibosomalproteinS36,mitochondrial	1	1
RibosomalL28family	1	1
AutophagyproteinAtg8ubiquitinlike	1	1
Disorderedregionofunknownfunction(DUF5315)	1	1
UbiAprenyltransferasefamily	1	1

Mo25-like	1	1
RibosomalproteinL11methyltransferase(PrmA)	1	1
Uso1/p115likevesicletetheringprotein,headregion	1	1
Mitochondrialpyruvatecarriers	1	1
ProteasomematurationfactorUMP1	1	1
Proteinofunknownfunction(DUF1077)	1	1
SNARE-complexproteinSyntaxin-18N-terminus	1	1
Plastocyanin-likedomain	1	1
TubulinfoldingcofactorDCterminal	1	1
STIMATEfamily	1	1
Domainofunknownfunction(DUF1961)	1	1
Basicregionleucinezipper	1	1
FAM192A/Fyv6,N-terminaldomain	1	1
InnermembraneproteinimportcomplexsubunitTim54	1	1
KinetochoreproteinCHL4like	1	1
NucleotideexchangefactorSIL1	1	1
6,7-dimethyl-8-ribityllumazinesynthase	1	1
Hsp20/alphacrystallinfamily	1	1
Arp2/3complex,34kDsubunitp34-Arc	1	1
Vacuolarproteinsorting-associatedprotein26	1	1
CentromereproteinH(CENP-H)	1	1
Staphylococcalnucleasehomologue	1	1
Respiratory-chainNADHdehydrogenase,49Kdsubunit	1	1
Corticalproteinmarkerforcellpolarity	1	1
MitochondrialATPsynthaseBchainprecursor(ATP-synt_B)	1	1
Telomerelengthregulationprotein	1	1
DNArepairmetallo-beta-lactamase	1	1
Splicingfactor3Bsubunit1	3	3
CCAAT-bindingtranscriptionfactor(CBF-B/NF-YA)subunitB	1	1
ASF1likehistonechaperone	1	1

Suppressorofforkedprotein(Suf)	1	1
L-asparaginaseII	1	1
DASHcomplexsubunitDad4	1	1
YL1nuclearproteinC-terminaldomain	1	1
Got1/Sft2-likefamily	1	1
Profilin	1	1
CytochromecoxidaseassemblyproteinCOX16	1	1
PRP38family	1	1
VRR-NUCdomain	1	1
Survivalmotorneuron(SMN)interactingprotein1(SIP1)	1	1
Ribonucleotidereductaseinhibitor	1	1
RibosomalproteinS27	1	1
SRP40,C-terminaldomain	1	1
Glutaminase	1	1
Translationmachinery-associatedprotein16	1	1
BNRrepeat-likedomain	1	1
Mitochondrialglycoprotein	1	1
Gamma-glutamyltranspeptidase	1	1
Semialdehydedehydrogenase,NADbindingdomain	1	1
Carbonicanhydrase	1	1
Domainofunknownfunction(DUF1989)	1	1
Glucosamine-6-phosphateisomerases/6-phosphogluconolactonase	3	3
CysteinedioxygenasetypeI	1	1
MAS20proteinimportreceptor	1	1
Proteinofunknownfunction(DUF3112)	1	1
Gammainterferoninduciblelysosomalthiolreductase(GILT)	1	1
Cns1/TTC4Wheeldomain	1	1
TAP42-likefamily	1	1
Reticulon	1	1
AMMECR1	1	1

DASHcomplexsubunitAsk1	1	1
OPToligopeptidetransporterprotein	3	3
5-formyltetrahydrofolatecyclo-ligasefamily	1	1
Microfibril-associated/Pre-mRNAprocessing	1	1
Signalrecognitionparticlereceptorbetasubunit	1	1
Succinatedehydrogenase/Fumaratereductasetransmembranesubunit	3	3
TranslationinitiationfactorIF-3,C-terminaldomain	1	1
Phosphatidylethanolamine-bindingprotein	3	3
RibosomalL27protein	1	1
Proteinphosphataseinhibitor2(IPP-2)	1	1
UV-endonucleaseUvdE	1	1
Thymidylatekinase	1	1
GLE1-likeprotein	1	1
ACTdomain	1	1
Proteinofunknownfunction(DUF1115)	1	1
HydrophobicsurfacebindingproteinA	1	1
10TMAcylTransferasedomainfoundinCas1p	3	3
MitochondrialproteinPet127	1	1
Ornithinecyclodeaminase/mu-crystallinfamily	1	1
BCCIP	1	1
Proteinofunknownfunction(DUF2418)	1	1
ATPsynthase(F/14-kDa)subunit	1	1
MLdomain	1	1
Sec8exocystcomplexcomponentspecificdomain	1	1
Glycosyltransferasefamily4	1	1
K+potassiumtransporter	1	1
OrganicsolutetransporterOstalpha	1	1
SMI1/KNR4family(SUKH-1)	1	1
Putativediphthamidesynthesisprotein	1	1
MitosisproteinDIM1	1	1

Proteinofunknownfunction(DUF1769)	1	1
Polysaccharidebiosynthesis	1	1
Purinenucleosidepermease(NUP)	1	1
Fungalproteinofunknownfunction(DUF1748)	1	1
GlycosylHydrolaseFamily88	1	1
Oligosaccharyltransferase48kDasubunitbeta	1	1
IntegralmembraneproteinSlinkingtothetransGolginetwork	1	1
CytochromeoxidasecsubunitVIb	3	3
Phosphatidylinositol-4-phosphate5-Kinase	1	1
NACHTdomain	1	1
DNAreplicationandcheckpointprotein	1	1
CENP-Q,aCENPA-CADcentromerecomplexsubunit	1	1
Nop14-likefamily	1	1
ProteinadenylyltransferaseSelO	1	1
Smallacidicproteinfamily	1	1
RibosomeassociatedmembraneproteinRAMP4	1	1
TB2/DP1,HVA22family	1	1
RFXDNA-bindingdomain	1	1
Frag1/DRAM/Sfk1family	1	1
RibosomalproteinS13/S18	1	1
Trm112p-likeprotein	1	1
Nucleolarprotein12(25kDa)	1	1
Tropomyosinlike	1	1
Domainofunknownfunction(DUF4202)	1	1
CyanatelyaseC-terminaldomain	1	1
EukaryoticrRNAprocessingproteinEBP2	1	1
PhosphatidylinositolN-acetylglucosaminyltransferasesubunitY	1	1
Domainofunknownfunction(DUF4203)	1	1
RibosomalL38eproteinfamily	1	1
COG(conservedoligomericGolgi)complexcomponent,COG2	1	1

HAUSaugmin-likecomplexsubunit6N-terminus	1	1
OutermembraneproteinTOM13	1	1
60Kdinnermembraneprotein	1	1
Proteinofunknownfunction(DUF1688)	1	1
GIY-YIGcatalyticdomain	1	1
CoA-ligase	1	1
Glycosylhydrolasefamily12	1	1
N-acetyltransferase	7	7
LysMdomain	1	1
Vacuolarproteinsorting-associatedprotein62	1	1
RibosomalproteinL19	1	1
TranscriptionfactorTFIIHcomplexsubunitTfb5	1	1
RibosomalL22eproteinfamily	1	1
HhH-GPDsuperfamilybaseexcisionDNArepairprotein	1	1
Integralperoxisomalmembraneperoxin	1	1
Celldivisioncontrolprotein14,SINcomponent	1	1
Rer1family	1	1
Paf1	1	1
MoaEprotein	1	1
ATPsynthase(E/31kDa)subunit	1	1
HeterokaryonincompatibilityproteinHet-C	1	1
Rix1complexcomponentinvolvedin60Sribosomematuration	1	1
RanGTP-bindingprotein	1	1
RXT2-like,N-terminal	1	1
Riboflavinkinase	1	1
CopperfistDNAbindingdomain	1	1
DNArepairproteinCrb2Tudordomain	1	1
SYF2splicingfactor	1	1
homogentisate1,2-dioxygenase	3	3
Sen15protein	1	1

AhpC/TSAantioxidantenzyme	1	1
SCA7,zinc-bindingdomain	1	1
Beta-1,3-glucanase	1	1
Domainofunknownfunction(DUF4112)	1	1
Ribonucleotidereductase,smallchain	1	1
Signalrecognitionparticle9kDaprotein(SRP9)	1	1
Proteinofunknownfunction(DUF1682)	1	0
Cutinase	1	1
RibosomalL37aeproteinfamily	1	1
SaccharopinedehydrogenaseNADPbindingdomain	1	1
VanZlikefamily	1	1
Protein-tyrosinephosphatase	1	1
Regulatorofchromosomecondensation(RCC1)repeat	1	1
Sec34-likefamily	1	1
RINT-1/TIP-1family	1	1
Ca2+regulatorandmembranefusionproteinFig1	1	1
Programmedcelldeathprotein2,C-terminalputativedomain	1	1
NIF3(NGG1pinteractingfactor3)	1	1
RecQmediatedgenomeinstabilityprotein	1	1
Proteinofunknownfunction(DUF2370)	1	1
Protein-L-isoaspartate(D-aspartate)O-methyltransferase(PCMT)	1	1
Phosphopantetheineattachmentsite	1	1
GRIM-19protein	1	1
SOCE-associatedregulatoryfactorofcalciumhomoeostasis	1	1
Utp11protein	1	1
Domainofunknownfunction(DUF4440)	1	1
EndoplasmicReticulumOxidoreductin1(ERO1)	1	1
Vps54-likeprotein	1	1
RIO1family	1	1
RNAdependentRNApolymerase	1	1

StressresponsiveA/BBarrelDomain	1	1
Malate/L-lactatedehydrogenase	1	1
VEFS-Boxofpolycombprotein	1	1
Putativeserineesterase(DUF676)	1	1
Diacylglycerolacyltransferase	1	1
Rox3mediatorcomplexsubunit	1	1
SRP19protein	1	1
Uroporphyrinogendecarboxylase(URO-D)	1	1
Dor1-likefamily	1	1
Timelessprotein	1	1
Glycogensynthase	1	1
RibosomalproteinS6	3	3
Ribosomalproteins50S-L15,50S-L18e,60S-L27A	1	1
OHCUdecarboxylase	1	1
Polysaccharidepyruvyltransferase	1	1
Proteinofunknownfunction(DUF2470)	1	1
SNAREassociatedGolgiprotein	1	1
RibosomalproteinS6e	1	1
LsmADdomain	1	1
HITzincfinger	1	1
OsmC-likeprotein	1	1
Domainofunknownfunction(DUF5102)	1	1
MitochondrialATPaseinhibitor,IATP	1	1
Formate/nitritetransporter	1	1
MitochondrialribosomalproteinmL59	1	1
CytochromecoxidasesubunitIV	1	1
GYFdomain	1	1
Silencingdefective2N-terminalubiquitindomain	1	1
Plectin/S10domain	1	1
Proteinphosphataseinhibitor	3	3

Glu-tRNAGlnamidotransferaseCsubunit	1	1
SolubleNSFattachmentprotein,SNAP	1	1
BNRrepeat-containingfamilymember	1	1
Domainofunknownfunction(DUF4185)	1	1
ApolipoproteinO	1	1
RNA2'-phosphotransferase,Tpt1/KptAfamily	1	1
Double-strandedRNAbindingmotif	1	1
rRNA-processingproteinEfg1	1	1
Zinc-ribbonlikefamily	1	1
TIP41-likefamily	1	1
PeptidaseM76family	1	1
EukaryoticandarchaealDNAprimase,largesubunit	1	1
Proteinofunknownfunction(DUF3533)	1	1
Malesterilityprotein	0	1
KetopantoatereductasePanE/ApbA	5	7
Bacterialproteinofunknownfunction(DUF885)	0	1
Glycosyltransferasefamily28C-terminaldomain	0	1

**Supplementary data 2-Table 3 : predicted accessory-gene families of *Fusarium oxysporum*. f. sp *albedinis* strains.**		
**Gene families**	***Fusarium oxysporum*. f. sp *albedinis* 133**	***Fusarium oxysporum*. f. sp *albedinis* 9**
	**Gene count**	**Gene count**
Sugar(andother)transporter	278	291
AuxiliaryActivityfamily9(formerlyGH61)	21	19
Acyl-CoAdehydrogenase,C-terminaldomain	15	15
Phosphorylasesuperfamily	29	27
WDdomain,G-betarepeat	51	35
Fungalspecifictranscriptionfactordomain	692	693
N-terminaldomainofNWDNACHT-NTPase	13	9
Tc5transposaseDNA-bindingdomain	41	41
Ankyrinrepeats(3copies)	222	202
FungalZn(2)-Cys(6)binuclearclusterdomain	249	239
DDEsuperfamilyendonuclease	172	533
Zincfinger,C2H2type	43	51
Poly(ADP-ribose)polymerasecatalyticdomain	21	22
Helitronhelicase-likedomainatN-terminus	29	146
Reversetranscriptase(RNA-dependentDNApolymerase)	106	304
Cyclin,N-terminaldomain	31	34
RegulatorofGproteinsignalingdomain	9	9
Cutinase	19	19
Sodium:neurotransmittersymporterfamily	5	3
CoAbindingdomain	9	7
helix-turn-helix,Psqdomain	13	7
TransposaseIS4	62	92
E1-E2ATPase	23	31
DEAD/DEAHboxhelicase	35	31
Prolyloligopeptidasefamily	13	13

Tetratricopeptiderepeat	35	33
Conservedmidregionofcactin	1	1
Responseregulatorreceiverdomain	17	15
MULEtransposasedomain	35	165
CytochromeP450	305	312
Slx4endonuclease	1	1
Heterokaryonincompatibilityprotein(HET)	262	263
tRNAsynthetasesclassII(D,KandN)	7	7
MatE	9	9
FMN-dependentdehydrogenase	27	27
KRdomain	7	5
PIF1-likehelicase	31	25
CBSdomain	7	7
Zincfinger,ZZtype	3	3
Glycosylhydrolasesfamily2	33	31
Zinc-bindingdehydrogenase	113	115
Stress-inducedbacterialacidophilicrepeatmotif	5	5
GAG-pre-integrasedomain	5	5
RhoGAPdomain	5	5
Pectatelyase	23	23
AminotransferaseclassIandII	51	50
HMGL-like	5	5
FADbindingdomain	228	231
Carboxylesterasefamily	71	72
Proteinofunknownfunction(DUF3500)	11	11
Proteinofunknownfunction(DUF3435)	13	11
RNaseH-likedomainfoundinreversetranscriptase	13	94
HelicaseconservedC-terminaldomain	19	15
AlcoholdehydrogenaseGroES-likedomain	71	74
Middleorthirddomainofpeptidase_M16	7	7

Endonuclease-reversetranscriptase	22	19
CoA-ligase	1	1
Subtilasefamily	66	69
NterminusofRad21/Rec8likeprotein	5	9
Proteinofunknownfunction(DUF3295)	25	25
PutativeERtransporter,6TM,N-terminal	7	7
Ubiquitinfamily	7	7
Lateexocytosis,associatedwithGolgitransport	1	0
Mono-functionalDNA-alkylatingmethylmethanesulfonateN-term	1	1
Extracellulartail,of10TMputativephosphatetransporter	5	0
Voltage-dependentanionchannel	15	15
Up-frameshiftsuppressor2	1	1
Mo-cooxidoreductasedimerisationdomain	1	1
Regulatorofchromosomecondensation(RCC1)repeat	9	11
Histidinekinase-,DNAgyraseB-,andHSP90-likeATPase	15	13
Adenylatecyclaseassociated(CAP)Cterminal	1	1
Sec23/Sec24trunkdomain	1	1
Glycosyltransferasefamily41	1	1
RecF/RecN/SMCNterminaldomain	3	3
PutativeTPR-likerepeat	1	1
Sec7domain	3	3
DNApolymerasebetathumb	1	1
Glycosylhydrolasefamily47	9	9
EF-handdomainpair	3	3
Ringhydroxylatingalphasubunit(catalyticdomain)	9	9
Ubiquitincarboxyl-terminalhydrolase,family1	3	3
SAPdomain	1	9
Proteinkinasedomain	187	197
Ferricreductaseliketransmembranecomponent	21	19
CAP-Glydomain	1	1

Pre-mRNA3'-end-processingendonucleasepolyadenylationfactorC-term	1	1
RNA12protein	1	1
AMP-bindingenzyme	91	87
Calcineurin-likephosphoesterase	31	33
IMPdehydrogenase/GMPreductasedomain	1	1
PutativeTOS1-likeglycosylhydrolase(DUF2401)	1	1
Proteinofunknownfunction(DUF3433)	9	7
GolgiCORVETcomplexcorevacuolarprotein8	1	1
Ubiquitincarboxyl-terminalhydrolase	13	11
RNArecognitionmotif.(a.k.a.RRM,RBD,orRNPdomain)	45	45
non-haemdioxygenaseinmorphinesynthesisN-terminal	15	13
Glycosylhydrolasefamily3C-terminaldomain	45	40
Sel1repeat	5	5
Zincknuckle	15	25
Autophagy-relatedprotein2CADmotif	1	1
Spermine/spermidinesynthasedomain	1	1
Conidiationprotein6	1	1
RibosomalRNA-processingprotein7(RRP7)C-terminaldomain	1	1
Glutaminesynthetase,catalyticdomain	7	7
HhH-GPDsuperfamilybaseexcisionDNArepairprotein	3	3
Bacterialalpha-L-rhamnosidase6hairpinglycosidasedomain	33	33
Cytochromedomainofcellobiosedehydrogenase	9	9
SpoUrRNAMethylasefamily	1	1
YL1nuclearprotein	1	1
Domainofunknownfunction(DUF1744)	1	1
Eukaryotictranslationinitiationfactor3subunit8N-terminus	1	1
RibosomalproteinS10p/S20e	1	1
Oxidoreductasefamily,NAD-bindingRossmannfold	25	33
UEVdomain	1	1
Glycosylhydrolasefamily45	1	1

RNApolymeraseRpb2,domain6	1	1
ElongationfactorTuGTPbindingdomain	9	9
NADH:flavinoxidoreductase/NADHoxidasefamily	39	43
ABC-2typetransporter	30	31
RibonucleasesP/MRPproteinsubunitPOP1	1	1
GlutathioneS-transferase,C-terminaldomain	5	5
AAAdomain	27	24
C-5cytosine-specificDNAmethylase	3	3
Prenylcysteinelyase	1	1
VID27C-terminalWD40-likedomain	1	1
Phosphatidylinositol3-and4-kinase	3	3
Glutamineamidotransferaseclass-I	5	5
NFACTproteinC-terminaldomain	1	1
Phosphatidylinositol-specificphospholipaseC,Xdomain	13	15
Cellulase(glycosylhydrolasefamily5)	19	19
ABCtransportertransmembraneregion	35	31
RibosomalproteinS2	7	7
MRG	1	1
Asparaginase	7	7
Thiolase,N-terminaldomain	9	7
FerricreductaseNADbindingdomain	11	11
Sec23-bindingdomainofSec16	1	1
SnoaL-likedomain	24	22
TLCdomain	3	3
ATPasefamilyassociatedwithvariouscellularactivities(AAA)	45	43
Myb-likeDNA-bindingdomain	23	25
Aminotransferaseclass-III	17	17
Pep3/Vps18/deeporangefamily	1	1
Domainofunknownfunction(DUF3517)	1	1
SAPK-interactingprotein1(Sin1),middleCRIMdomain	1	1

Arginasefamily	11	11
Anaphase-promotingcomplex,cyclosome,subunit4	1	1
PhenylalanyltRNAsynthetasebetachainCLMdomain	1	1
Intron-bindingproteinaquariusN-terminus	1	1
MIF4Gdomain	1	1
Fcf1	1	1
ribosomalL5PfamilyC-terminus	1	1
MajorFacilitatorSuperfamily	541	560
TOM7family	1	1
Cohesinloadingfactor	1	1
ABCtransportertransmembraneregion2	1	1
Carbon-nitrogenhydrolase	27	27
Elongationfactor1gamma,conserveddomain	1	1
PeptidaseC13family	1	1
MoeAN-terminalregion(domainIandII)	3	3
Fasciclindomain	9	9
Domainofunknownfunction(DUF1729)	5	5
Beta-lactamasesuperfamilydomain	13	11
Glycolipid2-alpha-mannosyltransferase	3	3
Initiationfactor2subunitfamily	1	1
DILdomain	1	1
Allantoicaserepeat	1	1
impB/mucB/samBfamily	5	5
Myosinhead(motordomain)	1	1
UBA-likedomain	1	1
ForminHomology2Domain	1	1
Velvetfactor	3	3
Ankyrinrepeats(manycopies)	15	13
SerinecarboxypeptidaseS28	1	1
RNaseH	33	120

Aminoacidpermease	173	178
GMCoxidoreductase	47	54
Beta-lactamase	55	51
RibosomalproteinS5,N-terminaldomain	1	1
Ferroportin1(FPN1)	1	1
Putativeserineesterase(DUF676)	7	7
AconitaseX	1	1
Glycosylhydrolasesfamily31	13	13
PeptidasefamilyM20/M25/M40	15	15
ThiFfamily	3	3
Calcium-dependentchannel,7TMregion,putativephosphate	9	11
Pyrroline-5-carboxylatereductasedimerisation	1	1
Catalase	9	9
Glycosyltransferasefamily20	3	3
MutSdomainV	7	7
SaccharopinedehydrogenaseC-terminaldomain	1	0
RTA1likeprotein	66	64
Glycosylhydrolasesfamily2,sugarbindingdomain	3	3
Phosphoribulokinase/Uridinekinasefamily	3	3
PX-associated	1	1
Vps16,N-terminalregion	1	1
FHAdomain	1	1
Haem-degrading	1	1
SNF2-relateddomain	35	35
ABCtransporter	61	57
Endonuclease/Exonuclease/phosphatasefamily	7	7
Iron/manganesesuperoxidedismutases,C-terminaldomain	1	1
ATP-NADkinaseC-terminaldomain	1	1
Squalene-hopenecyclaseC-terminaldomain	1	1
Splicingfactor1helix-hairpindomain	1	1

Carboxyltransferasedomain,subdomainAandB	1	1
Lyase	7	7
Tubulin/FtsZfamily,GTPasedomain	7	7
Alpha-L-arabinofuranosidaseB,catalytic	1	1
Patatin-likephospholipase	21	29
DNAreplicationfactorDna2	1	1
MnmEhelicaldomain	1	1
Glyoxalase/Bleomycinresistanceprotein/Dioxygenasesuperfamily	21	23
N-Acetylglucosaminyltransferase-IV(GnT-IV)conservedregion	3	3
ADP-ribosylationfactorfamily	11	11
Domainofunknownfunction(DUF5597)	5	5
Actin	11	11
Isocitrate/isopropylmalatedehydrogenase	9	9
DnaJdomain	13	15
U1smallnuclearribonucleoproteinof70kDaMWNterminal	1	1
Nucleartransportfactor2(NTF2)domain	1	1
Glycosylhydrolasefamily20,catalyticdomain	5	5
Glucose-6-phosphatedehydrogenase,C-terminaldomain	1	1
Choline/Carnitineo-acyltransferase	1	1
RibosomalproteinL35Ae	1	1
BCS1Nterminal	5	5
Ubiquitin-conjugatingenzyme	15	15
CationtransportingATPase,C-terminus	15	15
PhoDrelatedphosphatase	3	3
PeptidaseM16inactivedomain	1	1
SERRATE/Ars2,N-terminaldomain	1	1
Nas2N_terminaldomain	1	1
ATPsynthasealpha/betafamily,nucleotide-bindingdomain	5	5
Sulfatepermeasefamily	5	11
KHdomain	3	3

Glycosylhydrolasefamily10	11	13
Not1N-terminaldomain,CCR4-Notcomplexcomponent	1	1
Glycosylhydrolasesfamily43	39	44
SH3domain	9	9
EAP30/Vps36family	1	1
PI31proteasomeregulatorN-terminal	1	1
NADbindingdomainof6-phosphogluconatedehydrogenase	15	19
DIE2/ALG10family	1	1
N2,N2-dimethylguanosinetRNAmethyltransferase	1	1
Asparaginase,N-terminal	5	5
Mitochondrialcarrierprotein	25	27
Thermolysinmetallopeptidase,alpha-helicaldomain	1	1
Oxoglutarateandiron-dependentoxygenasedegradationC-term	1	1
Phospholipase/Carboxylesterase	7	7
ExocystcomplexcomponentSec3	1	1
Rieske[2Fe-2S]domain	0	0
Permeaseforcytosine/purines,uracil,thiamine,allantoin	19	21
Glycosylhydrolasesfamily2,TIMbarreldomain	5	5
Domainofunknownfunction(DUF5601)	1	1
Kelchmotif	9	9
Platelet-activatingfactoracetylhydrolase,isoformII	7	7
Copperamineoxidase,enzymedomain	13	15
Seryl-tRNAsynthetaseN-terminaldomain	1	1
Aldehydedehydrogenasefamily	83	87
Prolyl-tRNAsynthetase,C-terminal	3	1
LIMdomain	3	3
Eukaryoticporin	1	1
RNAhelicase(UPF2interactingdomain)	1	1
Glycosylhydrolasefamily7	31	31
SH2domain	1	1

DWNNdomain	1	1
MmgE/PrpDN-terminaldomain	3	3
NmrA-likefamily	164	152
2-oxoaciddehydrogenasesacyltransferase(catalyticdomain)	7	7
NucleoporinFGrepeatregion	3	3
Alpha/betahydrolasefamily	64	67
Polysaccharidedeacetylase	13	15
Chitinsynthase	9	9
UDP-glucose/GDP-mannosedehydrogenasefamily,centraldomain	5	5
Voltagegatedchloridechannel	3	3
Rapamycin-insensitivecompanionofmTOR,N-term	1	1
Trypsin-likepeptidasedomain	3	3
Alkalimetalcation/H+antiporterNha1Cterminus	1	1
HomodimerisationdomainofSGTA	1	1
SecondBRCTdomainonNijmegensyndromebreakageprotein	1	1
TRF2-interactingtelomericprotein/Rap1-Cterminaldomain	1	1
MetalbindingdomainofAda	1	1
Vaultproteininter-alpha-trypsindomain	3	3
N-terminaldomainoflipoylsynthaseofRadical_SAMfamily	1	1
Pectatelyasesuperfamilyprotein	3	3
Thioredoxin	15	15
Multicopperoxidase	23	25
Domainofunknownfunction(DUF4387)	1	3
GDSL-likeLipase/Acylhydrolasefamily	31	31
Oxidoreductasemolybdopterinbindingdomain	9	7
shortchaindehydrogenase	243	243
Galactoseoxidase,centraldomain	5	5
CHATdomain	44	44
D-isomerspecific2-hydroxyaciddehydrogenase,NADbindingdomain	19	19
GlutathioneS-transferase,N-terminaldomain	31	31

Sulfatase-modifyingfactorenzyme1	3	3
N-terminaldomainofoxidoreductase	3	3
Alpha-kinasefamily	3	3
ATPadenylyltransferaseC-terminaldomain	5	5
Malesterilityprotein	15	19
DipeptidylpeptidaseIV(DPPIV)N-terminalregion	1	1
JAB1/Mov34/MPN/PAD-1ubiquitinprotease	1	1
Aconitasefamily(aconitatehydratase)	5	5
Glycosylhydrolasesfamily18	43	48
Endoplasmicreticulum-basedfactorforassemblyofV-ATPase	1	1
Fumarylacetoacetate(FAA)hydrolasefamily	30	30
Proteinofunknownfunction(DUF3140)	1	1
PQlooprepeat	5	5
Flavin-bindingmonooxygenase-like	67	73
Protein-tyrosinephosphatase	1	1
lactate/malatedehydrogenase,alpha/betaC-terminaldomain	1	1
FADdependentoxidoreductase	83	85
PPRrepeat	3	3
Striatinfamily	1	1
Glycerol-3-phosphateacyltransferaseC-terminalregion	1	1
Domainofunknownfunction(DUF4139)	1	1
FYVEzincfinger	1	1
PeptidasefamilyM28	7	7
Phosphoribosylglycinamidesynthetase,ATP-grasp(A)domain	2	3
N-terminaldomainofribosephosphatepyrophosphokinase	1	1
KuCterminaldomainlike	1	1
COG4transportprotein	3	3
Dihydrofolatereductase	1	1
Dynaminfamily	15	17
Beta-ketoacylsynthase,N-terminaldomain	17	17

Telomererecombination	3	3
OPToligopeptidetransporterprotein	21	21
WSCdomain	23	21
C-terminalregionofMMR_HSR1domain	1	1
MCMP-loopdomain	5	5
RibosomalproteinL7/L12C-terminaldomain	1	1
NOG1N-terminalhelicaldomain	1	1
Tyrosyl-DNAphosphodiesterase	1	1
Fungaltrichotheceneeffluxpump(TRI12)	7	9
Subunit21ofMediatorcomplex	1	1
Fattyaciddesaturase	13	13
Eukaryotictranslationinitiationfactor2alphasubunit	1	1
Originrecognitioncomplex(ORC)subunit4C-terminus	1	1
Domainofunknownfunction(DUF383)	1	1
InhibitorofApoptosisdomain	1	1
Biotin-proteinligase,Nterminal	1	1
Acetyl-coenzymeAtransporter1	1	1
MOSCdomain	11	11
Bromodomain	9	11
ENTHdomain	3	3
ProbableN6-adeninemethyltransferase	1	1
CRAL/TRIOdomain	5	5
Importinrepeat6	1	1
EnolaseC-terminaldomain-like	13	13
Proteinofunknownfunction(DUF3638)	11	15
Importin-betaN-terminaldomain	1	1
Pyridoxal-phosphatedependentenzyme	21	21
Transglutaminase-likesuperfamily	3	3
eIF4-gamma/eIF5/eIF2-epsilon	1	1
Glycosylhydrolasesfamily32N-terminaldomain	8	9

Fes/CIP4,andEFC/F-BARhomologydomain	1	1
alpha/betahydrolasefold	150	156
Permeasefamily	5	5
Glutathione-dependentformaldehyde-activatingenzyme	19	21
CCR4-Notcomplexcomponent,Not1	1	1
Thiaminepyrophosphateenzyme,C-terminalTPPbindingdomain	3	3
Amidohydrolasefamily	35	33
Na+dependentnucleosidetransporterC-terminus	1	1
Condensationdomain	7	7
Pleckstrinhomologydomain	7	5
Clr5domain	17	17
Domainofunknownfunction(DUF3425)	41	49
Polynucleotidekinase3phosphatase	1	1
Zincfinger,C3HC4type(RINGfinger)	11	11
Molybdopterin-bindingdomainofaldehydedehydrogenase	3	3
JmjCdomain,hydroxylase	5	5
Glycerophosphoryldiesterphosphodiesterasefamily	5	5
RNApolymeraseI-specifictranscription-initiationfactor	1	1
Sec1family	1	1
Helicaseassociateddomain(HA2)	1	1
KetopantoatereductasePanE/ApbA	9	5
VAD1AnalogofStAR-relatedlipidtransferdomain	3	3
eRF1domain2	1	1
LysMdomain	11	11
NADPH-dependentFMNreductase	7	7
X-Prodipeptidyl-peptidase(S15family)	15	15
Aldo/ketoreductasefamily	69	67
Acyl-CoAdehydrogenase,N-terminaldomain	1	1
CrcB-likeprotein,CamphorResistance(CrcB)	1	1
(2R)-phospho-3-sulfolactatesynthase(ComA)	1	1

RibonucleaseIIIdomain	3	3
TranscriptionfactorPAP1	1	1
Piwidomain	7	7
Sodium/calciumexchangerprotein	15	17
Prenyltransferaseandsqualeneoxidaserepeat	3	3
Hexokinase	5	5
S-adenosylmethioninesynthetase,C-terminaldomain	1	1
AICARFT/IMPCHasebienzyme	1	1
Questinoxidase-like	3	3
Annexin	3	3
OxidoreductaseNAD-bindingdomain	7	9
CNHdomain	1	1
NACdomain	3	3
Zinc-ribbon,C4HC2type	1	1
TranscriptionfactorS-II(TFIIS)	1	1
Phosphoinositide3-kinasefamily,accessorydomain(PIKdomain)	1	1
HCO3-transporterfamily	3	3
Pre-PUA-likedomain	1	1
Dak1domain	9	7
Cationtransportprotein	11	13
Haemolysin-IIIrelated	3	3
Invertebrate-AID/APOBEC-deaminase	1	1
Rab-GTPase-TBCdomain	13	13
Pyridinenucleotide-disulphideoxidoreductase	47	53
Transketolase,thiaminediphosphatebindingdomain	7	7
Class-IIDAHPsynthetasefamily	1	1
chorismatebindingenzyme	1	1
Amidase	65	74
Proteinofunknownfunction(DUF917)	3	3
Cytoskeletal-regulatorycomplexEFhand	5	5

BRO1-likedomain	1	1
Vesiculovirusphosphoprotein	1	1
Histidinephosphatasesuperfamily(branch1)	17	17
Possiblelysinedecarboxylase	3	3
PANdomain	20	20
BOP1NT(NUC169)domain	1	1
Putativenuclearenvelopeorganisationprotein	1	1
Pre-SETmotif	1	1
PolyApolymeraseheaddomain	1	1
FungalN-terminaldomainofSTANDproteins	19	15
RWDdomain	1	1
Domainofunknownfunction(DUF1929)	5	5
TranslationinitiationfactorSUI1	5	5
Flavincontainingamineoxidoreductase	56	56
Phosphoribosyltransferasedomain	3	3
Glycosylhydrolasefamily71	5	5
10TMAcylTransferasedomainfoundinCas1p	1	1
Poly-adenylatebindingprotein,uniquedomain	1	1
Electrontransferflavoprotein-ubiquinoneoxidoreductase,4Fe-4S	1	1
Thioredoxin-likedomain	1	1
Las1-like	1	1
mRNAcappingenzyme	5	5
Arrestin(orS-antigen),N-terminaldomain	3	3
TranscriptionfactorTfb2	1	1
DENN(AEX-3)domain	3	3
Uncharacterizedconservedprotein(DUF2278)	5	5
MannosidaseIg/CBM-likedomain	3	3
Dioxygenase	41	43
HydantoinaseB/oxoprolinase	3	3
Leucyl-tRNAsynthetase,Domain2	1	1

Adenylosuccinatesynthetase	5	5
SterolmethyltransferaseC-terminal	1	1
Methyltransferasedomain	196	200
tRNAsynthetaseclassIIcoredomain(G,H,P,SandT)	1	1
VariantSH3domain	3	3
PutativeGTPaseactivatingproteinforArf	9	9
EsterasePHBdepolymerase	1	1
Prion-inhibitionandpropagation	27	23
Nicotinatephosphoribosyltransferase(NAPRTase)family	1	1
TCP-1/cpn60chaperoninfamily	1	1
Phosphotransferaseenzymefamily	47	49
Alpha-L-fucosidase	3	3
PhoD-likephosphatase	5	5
Enoyl-(Acylcarrierprotein)reductase	104	122
Maltoseacetyltransferase	3	3
Adaptorcomplexesmediumsubunitfamily	1	1
Gprotein-coupledglucosereceptorregulatingGpa2	5	5
Cupin	47	45
2OG-Fe(II)oxygenasesuperfamily	27	29
Arylsulfotransferase(ASST)	5	5
tRNAsynthetasesclassI(I,L,MandV)	1	1
RAVEprotein1Cterminal	1	1
GTPase-activatorproteinforRas-likeGTPase	3	3
Histidine-specificmethyltransferase,SAM-dependent	5	5
DNAmismatchrepairproteinMlh1C-terminus	1	1
Aflatoxinregulatoryprotein	1	1
Ceramidase	3	3
AcyclicterpeneutilisationfamilyproteinAtuA	3	3
Cupindomain	17	15
Retinalpigmentepithelialmembraneprotein	21	21

Ionchannel	5	5
Peroxidase	27	25
SETdomain	19	28
Aminomethyltransferasefolate-bindingdomain	3	3
Lethalgiantlarvae(Lgl)like,C-terminal	1	1
Sec63Brldomain	3	3
Proteasome-substrate-sizeregulator,midregion	1	1
HECT-domain(ubiquitin-transferase)	3	3
Melibiase	5	3
Sugar-bindingN-terminaldomain	3	3
PCRFdomain	1	1
Peptidasedimerisationdomain	5	5
Glycosylhydrolasefamily49N-terminalIg-likedomain	1	1
ScavengermRNAdecappingenzyme(DcpS)N-terminal	1	1
GammatubulincomplexcomponentC-terminal	1	1
RIO1family	3	3
RPN1N-terminaldomain	1	1
LrgB-likefamily	3	3
Porphobilinogendeaminase,dipyromethanecofactorbindingdomain	1	1
SRP54-typeprotein,GTPasedomain	1	1
NUDIXdomain	22	22
Metallo-beta-lactamasesuperfamily	31	31
High-temperature-induceddauer-formationprotein	1	1
Cupin-likedomain	11	11
GATAzincfinger	19	19
CyclinD1bindingdomain	1	1
Pyruvatekinase,barreldomain	1	1
AAAliddomain	3	3
DASHcomplexsubunitSpc34	1	1
Oligonucleotide/oligosaccharide-binding(OB)-fold	3	3

NACHTdomain	57	53
Commoncentraldomainoftyrosinase	29	35
Spb1C-terminaldomain	1	1
tRNAsynthetasesclassI(M)	1	1
FADlinkedoxidases,C-terminaldomain	11	13
Proteinofunknownfunction(DUF3723)	13	15
Ca2+insensitiveEFhand	1	1
G-patchdomain	3	3
GlutathioneS-transferaseN-terminaldomain	3	3
Aldolase/RraA	7	7
Bacterialtandemrepeatdomain1	1	1
SPRYdomain	1	1
AlphagalactosidaseA	3	3
Oxysterol-bindingprotein	5	5
Phosphoenolpyruvatecarboxykinase	1	1
Glycosylhydrolasefamily67middledomain	5	5
Proteintyrosinephosphatase-likeprotein,PTPLA	3	3
Tti2family	1	1
BacterialsignallingproteinNterminalrepeat	15	11
CoA-transferasefamilyIII	15	15
UvrD-likehelicaseC-terminaldomain	1	1
Domainofunknownfunction(DUF2014)	1	1
STEliketranscriptionfactor	1	1
Glycosylhydrolasefamily3Nterminaldomain	7	7
Dehydratasefamily	3	3
Pyruvatephosphatedikinase,AMP/ATP-bindingdomain	1	1
Domainofunknownfunction(DUF4743)	1	1
Calpainfamilycysteineprotease	25	29
Poly(A)polymerasecentraldomain	1	1
Zinc-fingerofnitricoxidesynthase-interactingprotein	1	1

Fungalysinmetallopeptidase(M36)	1	1
Anaerobicribonucleoside-triphosphatereductase	1	1
Transketolase,pyrimidinebindingdomain	3	3
Glycosyltransferasefamily28N-terminaldomain	25	23
Bacterialtransferasehexapeptide(sixrepeats)	3	3
Tannaseandferuloylesterase	23	25
Alpha-L-rhamnosidaseN-terminaldomain	1	1
Domainofunknownfunction(DUF427)	1	1
Alphaamylase,catalyticdomain	11	7
Domainofunknownfunction(DUF4470)	3	3
FADbindingdomainofDNAphotolyase	1	1
XPC-bindingdomain	1	1
ZincfingerC-x8-C-x5-C-x3-Htype(andsimilar)	9	9
HelicaseC-terminaldomain	1	1
BRE1E3ubiquitinligase	1	1
Sulfotransferasedomain	27	23
Molybdopterinoxidoreductase	3	3
Mannosyltransferaseputative	5	5
MetallopeptidasefamilyM24	7	7
RadicalSAMsuperfamily	1	1
Glycosylhydrolasefamily30betasandwichdomain	1	1
NB-ARCdomain	9	7
fungalSTANDN-terminalGoodbyedomain	11	11
FG-GAP-likerepeat	3	3
Uracilphosphoribosyltransferase	1	1
Zn-fingerinRanbindingproteinandothers	1	1
Cytochromeb5-likeHeme/Steroidbindingdomain	11	11
Xrn1helicaldomain	1	1
Domainofunknownfunction(DUF4111)	1	1
Domainofunknownfunction(DUF382)	1	1

Arb2domain	1	1
Membranemagnesiumtransporter	1	1
SLA1homologydomain1,SHD1	1	1
SnoaL-likepolyketidecyclase	13	13
Glycosylhydrolasesfamily35	5	5
Oxidoreductasefamily,C-terminalalpha/betadomain	3	3
EamA-liketransporterfamily	1	1
Dyneinassociatedprotein	1	1
Animalhaemperoxidase	7	9
Glycosylhydrolasefamily115	3	3
Prp19/Pso4-like	1	1
Thiaminepyrophosphateenzyme,N-terminalTPPbindingdomain	23	23
GlyoxaloxidaseN-terminus	1	1
Eukaryotic-typecarbonicanhydrase	5	5
DomainofUnknownFunction(DUF913)	1	1
Domainofunknownfunction(DUF1771)	1	1
Insulinase(PeptidasefamilyM16)	3	1
TypeI3-dehydroquinase	1	1
Shikimatedehydrogenasesubstratebindingdomain	1	1
Phospholipid-translocatingP-typeATPaseC-terminal	1	1
Nsp1-likeC-terminalregion	1	1
14-3-3protein	7	7
GETcomplexsubunitGET2	1	1
Transmembraneaminoacidtransporterprotein	31	29
Glycosylhydrolasesfamily15	1	1
Proteinofunknownfunction(DUF974)	1	1
IBRdomain,ahalfRING-fingerdomain	11	11
Alanineracemase,N-terminaldomain	3	1
Aspartylprotease	1	1
ATP-graspN-terminaldomain	1	1

GDP-mannose4,6dehydratase	5	3
Proteasomesubunit	7	7
Domainofunknownfunction(DUF1993)	5	5
Aminoacidkinasefamily	3	3
UbiAprenyltransferasefamily	3	3
Serineaminopeptidase,S33	23	23
Serinedehydratasealphachain	1	1
TaurinecatabolismdioxygenaseTauD,TfdAfamily	15	16
PeptidasefamilyM13	3	3
CVNHdomain	1	1
Arrestin(orS-antigen),C-terminaldomain	7	7
MCMAAA-liddomain	1	1
ScaffoldproteinNfu/NifUNterminal	1	0
Dihydrouridinesynthase(Dus)	5	5
Anaphase-promotingcomplexsubunit4WD40domain	1	1
ErgosterolbiosynthesisERG4/ERG24family	9	9
SAMdomain(Sterilealphamotif)	1	1
Sulfatase	23	21
Proteinofunknownfunction(DUF933)	1	1
Bacterialalpha-L-rhamnosidaseC-terminaldomain	1	1
CentromerekinetochorecomponentCENP-Thistonefold	5	5
Fusaricacidresistanceprotein-like	1	1
6-phosphogluconatedehydrogenase,C-terminaldomain	3	3
PULdomain	1	1
GTPcyclohydrolaseNterminal	1	1
Enoyl-CoAhydratase/isomerase	17	19
WWdomain	9	9
Uncharacterizedalpha/betahydrolasedomain(DUF2235)	19	17
ATPsynthasesubunitC	1	1
Ribonuclease2-5A	1	1

GlucosidaseIIbetasubunit-likeprotein	1	1
Proteinofunknownfunction(DUF3445)	3	5
VHSdomain	3	3
Ribosomebiogenesisprotein,C-terminal	1	1
RanBP1domain	3	3
Ig-likedomainfromnexttoBRCA1gene	1	1
MannitoldehydrogenaseC-terminaldomain	1	1
Eukaryoticaspartylprotease	30	30
Asp/Glu/Hydantoinracemase	11	9
Peroxidase,family2	13	11
NAD(P)-bindingRossmann-likedomain	21	27
tRNAsynthetasesclassII(A)	1	1
Glycosidehydrolasefamily2C-terminaldomain5	1	1
CoatomerWDassociatedregion	1	1
PLU-1-likeprotein	1	1
Proteinofunknownfunction(DUF3292)	1	1
Candidaagglutinin-like(ALS)	3	1
NAD(P)H-binding	46	49
KetopantoatereductasePanE/ApbACterminal	5	3
Glycosylhydrolasefamily79C-terminalbetadomain	5	5
NYNdomain	5	5
Cid1familypolyApolymerase	1	1
Proteinofunknownfunction(DUF1593)	9	11
HomeoboxKNdomain	11	11
Pectinesterase	8	8
EVEdomain	1	1
MoaFC-terminaldomain	1	1
NDT80/PhoGlikeDNA-bindingfamily	7	7
PeptidaseA4family	5	5
ATP-graspdomain	1	1

Pyridoxal-dependentdecarboxylase,pyridoxalbindingdomain	3	3
DSHCT(NUC185)domain	1	1
Iron/zincpurpleacidphosphatase-likeproteinC	1	1
Glycosyltransferasefamilygroup2	5	7
Glycosylhydrolasefamily59	1	1
TransportproteinAvl9	1	1
Domainofunknownfunction(DUF4419)	5	5
Ninonebinding(NOB1)Zn-ribbonlike	1	1
Cyclophilintypepeptidyl-prolylcis-transisomerase/CLD	9	9
ACTdomain	1	1
lactate/malatedehydrogenase,NADbindingdomain	3	3
Glycosyltransferasesgroup1	9	9
NADdependentepimerase/dehydratasefamily	58	60
Amidohydrolase	57	53
3-hydroxyacyl-CoAdehydrogenase,NADbindingdomain	3	5
ThreoninesynthaseNterminus	1	1
Dienelactonehydrolasefamily	27	27
RAMsignallingpathwayprotein	1	1
MembraneboundO-acyltransferasefamily	3	1
DHHA2domain	1	1
hATfamilyC-terminaldimerisationregion	94	496
Glycosylhydrolasesfamily28	21	19
Metallo-peptidasefamilyM12	1	1
FattyacidsynthasesubunitalphaAcylcarrierdomain	1	1
SRF-typetranscriptionfactor(DNA-bindinganddimerisationdomain)	9	15
Thiolase,C-terminaldomain	1	1
CoenzymeAtransferase	3	1
THOcomplexsubunit2N-terminus	1	1
EZH2N-terminaldomain	3	3
Seedmaturationprotein	1	1

Cationeffluxfamily	9	9
X-Prodipeptidyl-peptidaseC-terminalnon-catalyticdomain	3	3
Tetrahydrofolatedehydrogenase/cyclohydrolase,catalyticdomain	1	1
GlycinecleavagesystemP-protein	1	1
Pyoverdine/dityrosinebiosynthesisprotein	1	1
Glycosyltransferasesugar-bindingregioncontainingDXDmotif	5	5
Tyrosinephosphatasefamily	3	3
mRNAcappingenzyme,catalyticdomain	1	1
Telomeraseribonucleoproteincomplex-RNAbindingdomain	1	1
S-adenosyl-L-homocysteinehydrolase,NADbindingdomain	1	1
RibonucleaseT2family	3	3
EPSPsynthase(3-phosphoshikimate1-carboxyvinyltransferase)	1	1
Carbohydrateesterase2N-terminal	1	1
Glutamate/Leucine/Phenylalanine/Valinedehydrogenase	3	5
ERCC3/RAD25/XPBC-terminalhelicase	1	1
HNHendonuclease	25	31
GLEYAdomain	5	7
RecQzinc-binding	3	0
LicDfamily	1	1
ANTHdomain	1	1
TAP-likeprotein	3	3
Frequencyclockprotein	17	17
Proteinofunknownfunction(DUF4246)	11	9
SIR2-likedomain	1	1
Pyridoxamine5'-phosphateoxidase	7	7
haloaciddehalogenase-likehydrolase	3	3
Acyltransferasedomain	3	1
Poly(ADP-ribose)polymerase,regulatorydomain	3	3
Glycosylhydrolasesfamily6	1	1
NuclearporeproteinNUP188C-terminaldomain	1	1

Indole-3-glycerolphosphatesynthase	1	1
Apg6BARAdomain	1	1
Integrasecoredomain	1	1
Glycosylhydrolasefamily76	13	13
PAP2superfamily	15	13
Polyketidesynthasedehydratase	1	3
OTT_1508-likedeaminase	25	25
FAD-bindingdomain	3	7
Iron-containingalcoholdehydrogenase	19	21
SesBdomainonfungaldeath-pathwayprotein	1	1
Dihydrodipicolinatesynthetasefamily	13	11
Glycosylhydrolasefamily65,N-terminaldomain	5	5
Uncharacterisedproteinfamily(UPF0183)	1	1
Formate/nitritetransporter	3	3
Chromatetransporter	5	5
Phagelysozyme	3	5
Caleosinrelatedprotein	5	5
Glycosylhydrolasefamily1	35	35
RasGEFdomain	1	1
Putativeserinedehydratasedomain	1	1
Proteinofunknownfunction(DUF3659)	5	5
Squalene-hopenecyclaseN-terminaldomain	1	1
MmgE/PrpDC-terminaldomain	1	1
tRNAsynthetasesclassI(R)	1	1
Mis12-Mtw1proteinfamily	13	13
Pirin	3	3
Orsellinicacid/F9775biosynthesisclusterproteinD	33	79
Zinccarboxypeptidase	11	11
SRR1	5	5
Clathrinadaptorcomplexsmallchain	1	1

SHNi-TPR	1	1
Zinc-bindingdomain	1	1
Transmembraneproteins14C	3	3
3-betahydroxysteroiddehydrogenase/isomerasefamily	11	9
Secretorylipase	7	7
Proteinofunknownfunction(DUF4238)	3	5
SMP-30/Gluconolactonase/LRE-likeregion	11	11
BTB/POZdomain	40	38
Cyclin-dependentkinaseregulatorysubunit	1	1
CPSFAsubunitregion	3	3
Phytanoyl-CoAdioxygenase(PhyH)	20	20
Domainofunknownfunction(DUF4345)	3	3
Kinesinmotordomain	5	5
Cysteine-richTMmodulestresstolerance	1	1
Proteinofunknownfunction(DUF3759)	1	1
Amino-transferaseclassIV	13	15
Alcoholacetyltransferase	11	11
YCII-relateddomain	5	5
SAMdependentcarboxylmethyltransferase	5	5
Aromaticaminoacidlyase	3	3
Lipocalin-likedomain	1	1
2EXRfamily	46	49
EpoxidehydrolaseNterminus	11	13
Isoprenylcysteinecarboxylmethyltransferase(ICMT)family	7	5
Majorintrinsicprotein	11	11
Nucleotide-diphospho-sugartransferase	3	3
Acetyltransferase(GNAT)family	45	43
IncreasedlossofmitochondrialDNAprotein1	1	1
EthDdomain	13	15
ChrRCupin-likedomain	5	5

Lactonase,7-bladedbeta-propeller	7	9
MycotoxinbiosynthesisproteinUstYa	3	3
Polyketidecyclase/dehydraseandlipidtransport	8	10
Indoleamine2,3-dioxygenase	7	7
NUBPLiron-transferP-loopNTPase	1	1
Basicregionleucinezipper	7	11
Proteintyrosineandserine/threoninekinase	3	1
Proteinofunknownfunction(DUF3455)	7	11
Ubiquinol-cytochromeCreductasecomplex14kDsubunit	1	1
Hsp20/alphacrystallinfamily	7	9
Up-regulatedDuringSeptation	13	13
Lipase(class3)	5	5
Glycosylhydrolasesfamily16	49	61
Uncharacterizedproteinconservedinbacteria(DUF2263)	5	5
Aldose1-epimerase	1	1
CorA-likeMg2+transporterprotein	9	5
GDSL-likeLipase/Acylhydrolase	39	39
Pro-kumamolisin,activationdomain	5	5
Cytidineanddeoxycytidylatedeaminasezinc-bindingregion	1	1
Necrosisinducingprotein(NPP1)	5	7
Proteinofunknownfunction(DUF2439)	1	1
EndoribonucleaseL-PSP	21	21
BRCTdomain,aBRCA1C-terminusdomain	1	1
FungalTransformingacidiccoiled-coil(TACC)proteins	1	1
Yip1domain	1	1
Bacterialphospho-glucoseisomeraseC-terminalSISdomain	1	1
Acetyltransferase(GNAT)domain	27	27
Metal-independentalpha-mannosidase(GH125)	3	3
Transferasefamily	24	24
alpha-L-rhamnosidase	41	41

Haem-containingdehydratase	7	7
F-boxdomain	21	23
Iontransportprotein	1	1
S1/P1Nuclease	1	1
Glycosyltransferasefamily8	9	9
O-methyltransferasedomain	21	27
GDP/GTPexchangefactorSec2p	1	1
3-hydroxyacyl-CoAdehydrogenase,C-terminaldomain	1	1
Trichodienesynthase(TRI5)	1	1
Proteinofunknownfunction(DUF1349)	3	3
Phosphoglyceratekinase	1	1
HpcH/HpaIaldolase/citratelyasefamily	19	15
Serinecarboxypeptidase	24	24
Histidinoldehydrogenase	1	1
TctoxincomplexTcAC-terminalTcB-bindingdomain	1	1
galactosyltransferaseGMA12/MNN10family	5	5
Sir2family	3	3
YetA-likeprotein	1	1
Eukaryoticproteinofunknownfunction(DUF829)	5	5
OrganicsolutetransporterOstalpha	3	3
F-box-like	31	31
Prolinedehydrogenase	3	3
Predictedmembraneprotein(DUF2306)	5	5
Phosphoenolpyruvatephosphomutase	5	5
Porphyromonas-typepeptidyl-argininedeiminase	3	3
Proteinofunknownfunction(DUF2456)	1	1
Ecdysteroidkinase-likefamily	11	11
KinetochoreSim4complexsubunitFTA2	10	11
Lysinemethyltransferase	3	3
Glycosyltransferaselikefamily2	5	5

StressresponsiveA/BBarrelDomain	5	5
homogentisate1,2-dioxygenase	3	3
Cys/MetmetabolismPLP-dependentenzyme	19	19
Terpenesynthasefamily2,C-terminalmetalbinding	11	13
Fungalproteinkinase	5	5
Cupinsuperfamily(DUF985)	3	3
Dihydroorotatedehydrogenase	1	1
DomainofUnknownFunction(DUF1907)	3	3
HDdomain	9	11
Haloaciddehalogenase-likehydrolase	15	15
Glycosidehydrolase131catalyticN-terminaldomain	1	1
Thioesterase-likesuperfamily	15	15
Glycosylhydrolasesfamily39	3	3
MeioticcellcortexC-terminalpleckstrinhomology	1	1
GlycosylHydrolaseFamily88	3	5
Lysophospholipasecatalyticdomain	5	5
Domainofunknownfunction(DUF1989)	3	1
Autophagocytosisassociatedprotein,active-sitedomain	5	4
SMI1/KNR4family(SUKH-1)	3	3
ADP-ribosylglycohydrolase	7	7
EsteraseFrsA-like	5	3
bZIPtranscriptionfactor	27	30
Mating-typeproteinMATalpha1HMG-box	1	1
Domainofunknownfunction(DUF1338)	9	9
Matingtypeprotein1-1-2ofunknownfunction	1	1
Proteinofunknownfunction(DUF1479)	11	9
ATPsynthasesubunitD	1	1
HMG(highmobilitygroup)box	5	5
GDA1/CD39(nucleosidephosphatase)family	1	1
ER-GolgitraffickingTRAPPIcomplex85kDasubunit	1	1

XPGdomaincontaining	1	1
Ubiquitin3bindingproteinBut2C-terminaldomain	5	5
CytochromecoxidasesubunitVIa	1	1
Phenazinebiosynthesis-likeprotein	7	7
Glycosylhydrolasefamily12	11	11
Helix-loop-helixDNA-bindingdomain	15	15
Sodium:solutesymporterfamily	3	3
RhamnogalacturonanIlyasesbeta-sheetdomain	1	1
Alginatelyase	5	5
Hypotheticalmethyltransferase	1	1
PredictedAdoMet-dependentmethyltransferase	1	1
pfkBfamilycarbohydratekinase	3	3
ChorismatemutasetypeII	1	1
Fungalproteinofunknownfunction(DUF1752)	19	19
Rhodanese-likedomain	5	5
SodiumBileacidsymporterfamily	5	5
Naturalresistance-associatedmacrophageprotein	5	5
NUDEprotein,C-terminalconservedregion	1	1
Dynactinp62family	1	1
Bacterialproteinofunknownfunction(DUF924)	3	3
TranscriptionfactorIIA,alpha/betasubunit	1	1
Hypoxiainducedproteinconservedregion	3	3
DNApolymeraseLigase(LigD)	1	1
UDP-glucoronosylandUDP-glucosyltransferase	7	7
Ankyrinrepeat	245	223
Trypsin	7	7
Proteinofunknownfunction(DUF3632)	5	5
Pyridoxal-dependentdecarboxylaseconserveddomain	11	11
Forkheaddomain	1	1
CoenzymeQ(ubiquinone)biosynthesisproteinCoq4	1	1

Transaldolase/Fructose-6-phosphatealdolase	7	7
Proteinofunknownfunction(DUF1688)	1	1
Acetoacetatedecarboxylase(ADC)	8	8
DJ-1/PfpIfamily	14	14
SaccharopinedehydrogenaseNADPbindingdomain	5	5
Astacin(PeptidasefamilyM12A)	5	7
Caspasedomain	11	11
Rasfamily	21	21
Bacteriorhodopsin-likeprotein	15	19
Fungalfruitbodylectin	1	1
Proteinofunknownfunction(DUF3237)	11	11
CFEMdomain	23	25
RibDC-terminaldomain	1	1
Antibioticbiosynthesismonooxygenase	7	7
Glycosyltransferasefamily90	7	7
Peroxisomalbiogenesisfactor11(PEX11)	3	3
Alpha/betahydrolasedomain	1	0
Thioesterasesuperfamily	9	7
MAPEGfamily	7	5
Tryptophandimethylallyltransferase	7	5
Phosphoesterasefamily	3	1
PP-loopfamily	1	1
Pex2/Pex12aminoterminalregion	1	1
vonWillebrandfactortypeAdomain	3	7
Serinehydrolase(FSH1)	3	3
LITAF-likezincribbondomain	11	11
Domainofunknownfunction(DUF1772)	3	3
Proteinofunknownfunction(DUF2985)	3	3
WD40-likeBetaPropellerRepeat	1	1
Ferritin-likedomain	3	5

Familyofunknownfunction(DUF5519)	3	3
ClassIIAldolaseandAdducinN-terminaldomain	5	5
HPC2andubinucleindomain	1	1
Golgiphosphoprotein3(GPP34)	1	1
Zincfingerprotein	1	1
CASC3/BarentszeIF4AIIIbinding	1	1
Familyofunknownfunction(DUF6060)	2	1
Proteinofunknownfunction(DUF3176)	9	9
Ribose/GalactoseIsomerase	3	3
FtsJ-likemethyltransferase	5	5
Isochorismatasefamily	11	11
MCRAfamily	1	3
PeptidasefamilyM3	3	3
Purinenucleosidepermease(NUP)	1	1
Cysteine-richsecretoryproteinfamily	15	19
FAR-17a/AIG1-likeprotein	1	1
Putativecyclase	11	11
Domainofunknownfunction(DUF4267)	11	9
TelomericsinglestrandedDNAbindingPOT1/CDC13	1	1
RibosomalproteinS14p/S29e	3	3
Beta-eliminatinglyase	1	1
Egh16-likevirulencefactor	9	7
TM2domain	1	3
Glycosyltransferasefamily64domain	3	3
hTAFII28-likeproteinconservedregion	1	1
GTPcyclohydrolaseI	1	1
Transientreceptorpotential(TRP)ionchannel	1	1
Glycine-richdomain-containingprotein-like	1	1
RING-variantdomain	1	1
Alpha-L-arabinofuranosidaseC-terminaldomain	5	5

Stc1domain	1	1
Rhomboidfamily	3	3
Luciferase-likemonooxygenase	5	5
Ham1family	1	1
ProteinHRI1	1	1
ECL1/2/3zincbindingproteins	5	7
K+potassiumtransporter	5	3
Arginosuccinatesynthase	3	3
Choline/ethanolaminekinase	3	3
Flavoprotein	7	7
HrpE/YscL/FliHandV-typeATPasesubunitE	1	1
Vacuolarproteinsorting-associatedprotein62	1	1
Transposase	73	104
U3-containing90Spre-ribosomalcomplexsubunit	1	1
Cryptococcalmannosyltransferase1	9	9
Pathogeneffector	3	3
Las17-bindingproteinactinregulator	5	7
Competence-damagedprotein	1	1
2'-5'RNAligasesuperfamily	1	1
Flavinreductaselikedomain	5	5
Meioticallyup-regulatedgenefamily	5	13
Sybindin-likefamily	3	3
Phosphatetransporterfamily	5	5
LanthioninesynthetaseC-likeprotein	1	1
Matrixin	1	1
Phosphatidylglycerollysyltransferase,C-terminal	1	1
Glutamineamidotransferasesclass-II	1	1
Hypotheticalglycosylhydrolase6	3	3
RQCdomain	1	1
GINScomplexprotein	3	3

Gamma-glutamylcyclotransferase,AIG2-like	6	6
Citratesynthase,C-terminaldomain	1	1
Glycosylhydrolasecatalyticcore	3	3
HicAtoxinofbacterialtoxin-antitoxin,	1	1
Familyofunknownfunction(DUF5695)	3	3
Spt20family	1	1
RNAdependentRNApolymerase	9	11
AFG1-likeATPase	1	1
Lipoxygenase	3	3
Sodium/hydrogenexchangerfamily	9	15
ActivatorofmitoticmachineryCdc14phosphataseactivationC-term	1	1
RPA43OBdomaininRNAPolI	1	1
Hemeoxygenase	1	1
Righthandedbetahelixregion	3	3
Putativeamidoligaseenzyme	5	5
Tuberculosisnecrotizingtoxin	1	1
Acyltransferasefamily	3	3
IonchannelregulatoryproteinUNC-93	3	3
Domainofunknownfunction(DUF4385)	1	1
Alpha-galactosyl-bindingfungallectin	1	1
Nucleoporinautopeptidase	1	1
Spherulation-specificfamily4	3	1
Glycoside-hydrolasefamilyGH114	3	3
Domainofunknownfunction(DUF5703)	1	1
Protein-argininedeiminase(PAD)	9	9
Exonuclease	15	15
PeptidasefamilyS41	1	1
Reactivemitochondrialoxygenspeciesmodulator1	1	1
Beta-1,3-glucanase	13	17
TENA/THI-4/PQQCfamily	1	1

C-terminaldomainof1-Cysperoxiredoxin	1	1
Glycosylhydrolasefamily53	3	3
Deuterolysinmetalloprotease(M35)family	1	1
Glutaredoxin	3	3
Bacterialextracellularsolute-bindingprotein	3	5
G-proteinalphasubunit	7	7
Arrestin_Nterminallike	3	3
Pregnancy-associatedplasmaprotein-A	3	3
ExpansinC-terminaldomain	1	1
CysteinedioxygenasetypeI	1	1
Scytalonedehydratase	1	3
Hsp70protein	19	19
Phenolicaciddecarboxylase(PAD)	1	1
Glucose-repressibleproteinGrg1	3	3
Mpv17/PMP22family	7	7
3-octaprenyl-4-hydroxybenzoatecarboxy-lyase	9	9
Cytochromec	15	15
Fic/DOCfamily	9	9
CopperfistDNAbindingdomain	1	1
VITfamily	1	1
EXPERA(EXPandedEBPsuperfamily)	5	5
Carboxymuconolactonedecarboxylasefamily	11	11
Putativelumazine-binding	3	1
Glycosyltransferasefamily2	33	29
Erg28likeprotein	1	1
Ulp1proteasefamily,C-terminalcatalyticdomain	11	15
Domainofunknownfunction(DUF4451)	5	5
Translocon-associatedprotein(TRAP),alphasubunit	1	1
Est1DNA/RNAbindingdomain	5	13
Tubulin-tyrosineligasefamily	3	3

Creatinase/ProlidaseN-terminaldomain	1	1
HeterokaryonincompatibilityproteinHet-C	3	3
Thiolase-likeproteintype1additionalC-terminaldomain	1	1
50Sribosome-bindingGTPase	5	5
Proteinofunknownfunction(DUF3533)	7	7
Putativeesterase	5	5
Uncharacterizedproteinconservedinbacteria(DUF2264)	1	1
Carbohydratebindingdomain	1	3
Beta-L-arabinofuranosidase,GH127	5	5
Fungalcellulosebindingdomain	3	3
Xyloseisomerase-likeTIMbarrel	5	9
D-arabinono-1,4-lactoneoxidase	1	1
Alternativeoxidase	1	1
PHPdomain	1	1
Erv1/Alrfamily	1	1
Familyofunknownfunction(DUF6055)	1	1
Hydroxymethylglutaryl-coenzymeAreductase	7	15
Glycosyltransferasefamily17	3	3
SNF5/SMARCB1/INI1	3	3
Phosphatidylserinedecarboxylase	7	11
NMT1-likefamily	1	1
PKDdomain	1	1
Caulimovirusviroplasmin	1	1
Proteinofunknownfunction(DUF2722)	3	1
Chromo(CHRromatinOrganisationMOdifier)domain	25	29
StageIIsporulationproteinE(SpoIIE)	1	1
Reticulon	9	7
C2H2typezinc-finger(2copies)	5	5
NuclearfragileXmentalretardation-interactingprotein1(NUFIP1)	1	1
Nuclearporecomplexscaffold,nucleoporins186/192/205	3	1

Zinc-finger	3	5
Lipopolysaccharidekinase(Kdo/WaaP)family	3	3
CyanatelyaseC-terminaldomain	1	1
Carbonicanhydrase	7	7
Cerato-platanin	1	1
Ctrcoppertransporterfamily	5	5
UV-endonucleaseUvdE	1	1
Innermembranecomponentdomain	1	1
gag-polypeptideofLTRcopia-type	5	3
Linalooldehydratase/isomerase	5	5
Proteinofunknownfunction(DUF1749)	1	1
AAAATPasedomain	3	3
DomainfoundinDishevelled,Egl-10,andPleckstrin(DEP)	1	1
Shortcoiled-coilprotein	5	7
Aminotransferaseclass-V	7	7
Septin	5	5
Putativeoxalocrotonatetautomeraseenzyme	1	1
SlimemoldcyclicAMPreceptor	5	5
Histidinephosphatasesuperfamily(branch2)	9	11
Domainofunknownfunction(DUF2431)	1	1
Damage-controlphosphataseARMT1-likedomain	1	1
Anhydro-N-acetylmuramicacidkinase	1	1
Thiamine-bindingprotein	1	3
NADHubiquinoneoxidoreductase,20Kdsubunit	1	1
Calcium-activatedchloridechannel	3	3
PDZ-likedomain	3	0
T5orf172domain	11	11
HemerythrinHHEcationbindingdomain	7	7
CentromereproteinScm3	1	1
Proteinofunknownfunction(DUF3807)	1	1

Vacuolarsorting38andautophagy-relatedsubunit14	1	1
Conservedhypotheticalprotein(DUF2461)	3	3
FAM32A	1	1
PescadilloN-terminus	1	1
Proteinofunknownfunction(DUF659)	5	17
CentromereDNA-bindingproteincomplexCBF3subunit,domain2	3	10
BRCA1CTerminus(BRCT)domain	1	1
Wingedhelix-turnhelix	1	1
Adenosinedeaminase	5	5
CHAPdomain	1	3
AdoMetdependentprolinedi-methyltransferase	3	1
Berberineandberberinelike	1	1
Neuraminidase-likedomain	1	1
7transmembranereceptor(Secretinfamily)	3	3
WLMdomain	3	3
PutativeFMN-bindingdomain	5	3
WGRdomain	1	1
Glycosylhydrolasesfamily11	1	1
ProkaryoticphospholipaseA2	3	3
HSF-typeDNA-binding	7	7
Nineteencomplex-relatedprotein2	1	1
LSMdomain	13	12
Homeodomain	5	5
ZIPZinctransporter	3	3
Bestrophin,RFP-TM,chloridechannel	5	7
SurvivalproteinSurE	5	5
PSP1C-terminalconservedregion	1	0
MYNDfinger	11	56
Erythromycinesterase	3	6
Domainofunknownfunction(DUF1996)	3	3

Nis1family	3	3
Copperamineoxidase,N2domain	1	1
PhospholipaseDActivesitemotif	1	1
Ringfingerdomain	13	11
Alpha-acetolactatedecarboxylase	3	0
Eukaryoticelongationfactor5Ahypusine,DNA-bindingOBfold	1	1
Domainofunknownfunction(DUF4939)	1	0
Proteinofunknownfunction(DUF3429)	1	1
ER-boundoxygenasempaB/B'/Rubberoxygenase,catalyticdomain	5	5
L-asparaginaseII	1	5
PASfold	1	1
CationtransportATPase(P-type)	1	0
Reversetranscriptase-like	1	3
FAR1DNA-bindingdomain	1	0
NAT,N-acetyltransferase,ofN-acetylglutamatesynthase	1	1
Proteinofunknownfunction(DUF3645)	1	0
PEP-utilisingenzyme,PEP-bindingdomain	1	0
Serineincorporator(Serinc)	1	1
SUKH-4immunityprotein	1	3
Cyclin,C-terminaldomain	1	0
TFIIEalphasubunit	1	1
Nuclearcap-bindingproteinsubunit3	1	1
SNAREdomain	1	1
RING-H2zincfingerdomain	3	3
CASPCterminal	1	1
Fibrillarin	1	1
mRNAcleavageandpolyadenylationfactorCLP1P-loop	1	1
DASHcomplexsubunitDad1	1	1
MitochondrialATPsynthaseepsilonchain	1	1
Mak10subunit,NatCN(alpha)-terminalacetyltransferase	1	1

Skp1family,tetramerisationdomain	1	1
V-typeATPase116kDasubunitfamily	1	1
ARP2/3complex16kDasubunit(p16-Arc)	1	1
Sds3-like	1	1
Proteinofunknownfunction(DUF1757)	1	1
Mechanosensitiveionchannel	1	1
Proteinofunknownfunction(DUF3128)	1	1
RibosomalL15	1	1
Complex1protein(LYRfamily)	5	5
Vps53-like,N-terminal	1	1
Dyneinlightchaintype1	1	1
Domainofunknownfunction(DUF1708)	1	1
FirstLongindomainofINTU,CCZ1andHPS4	1	1
Proteinphosphatase2C	1	1
Putativetransmembranefamily234	1	1
RegioninClathrinandVPS	1	1
Proteinofunknownfunction(DUF2462)	1	1
Coatomerepsilonsubunit	1	1
Proteinofunknownfunction(DUF1365)	1	1
ARID/BRIGHTDNAbindingdomain	3	3
SUR7/PalIfamily	7	7
MitochondrialbiogenesisAIM24	3	3
Rad52/22familydouble-strandbreakrepairprotein	1	1
ORMDLfamily	1	1
Frag1/DRAM/Sfk1family	1	1
tRNAsynthetasesclassI(WandY)	5	5
Tim10/DDPfamilyzincfinger	1	1
CUEdomain	1	1
PPRrepeatfamily	1	1
ALG6,ALG8glycosyltransferasefamily	1	1

Sin3associatedpolypeptidep18(SAP18)	1	1
Fcf2pre-rRNAprocessing	1	1
Histone-liketranscriptionfactor(CBF/NF-Y)andarchaealhistone	5	5
ExocystcomplexsubunitSec15-like	1	1
RNArecognitionmotif2	1	1
Sad1/UNC-likeC-terminal	1	1
PutativezincfingerinN-recognin(UBRbox)	1	1
KinetochorecomplexFta4ofSim4subunit,orCENP-50	1	1
Rftprotein	1	1
FungalpheromonematingfactorSTE2GPCR	1	1
N-acetylglucosaminyltransferasecomponent(Gpi1)	1	1
Proteinofunknownfunction(DUF1275)	3	1
WH1domain	1	1
RibosomalproteinL37e	1	1
Membranetransportprotein	3	3
RaptorN-terminalCASPaselikedomain	1	1
ABC1atypicalkinase-likedomain	1	1
MOZ/SASfamily	1	1
CytochromecoxidaseassemblyproteinPET191	1	1
Pal1cellmorphologyprotein	1	1
TranscriptioninitiationfactorTFIIDsubunitA	1	1
N-terminaldomainofCBF1interactingco-repressorCIR	1	1
Iron-sulphurclusterbiosynthesis	1	1
NucleoporinproteinNdc1-Nup	1	1
Gamma-glutamyltranspeptidase	3	3
TranscriptionfactorTfb4	1	1
Anaphase-promotingcomplex,subunit10(APC10)	1	1
linkerhistoneH1andH5family	1	1
D123	1	1
Proteinofunknownfunction(DUF3716)	1	17

ChromatinassociatedproteinKTI12	1	1
Uncharacterisedproteinfamily,YAP/Alf4/glomulin	1	1
Sterylacetylhydrolase	1	1
Sedlin,N-terminalconservedregion	3	3
PrpFprotein	1	1
CENP-SassociatingCentromereproteinX	1	1
Mitoticcheckpointregulator,MAD2B-interacting	1	1
S25ribosomalprotein	1	1
Macrophagemigrationinhibitoryfactor(MIF)	1	1
Proteinofunknownfunction(DUF2420)	1	1
Myotubularin-likephosphatasedomain	1	1
Der1-likefamily	3	3
TruBfamilypseudouridylatesynthase(Nterminaldomain)	1	1
RF-1domain	1	1
Urm1(Ubiquitinrelatedmodifier)	1	1
CSLzincfinger	1	1
CutCfamily	1	1
Alkalinephosphatase	1	1
Spt4/RpoE2zincfinger	1	1
Frataxin-likedomain	1	1
Chitinsynthaseexportchaperone	1	1
KAPfamilyP-loopdomain	1	1
ConservedhypotheticalATPbindingprotein	1	1
Rpp14/Pop5family	1	1
RibosomalL27eproteinfamily	1	1
TPR/MLP1/MLP2-likeprotein	1	1
DUF218domain	3	3
HistonechaperonedomainCHZ	1	1
Cyclin	43	44
Telomererepeatbindingfactor(TRF)	1	1

UncharacterizedproteinfamilyUPF0029	1	1
Eukaryoticproteinofunknownfunction(DUF866)	1	1
Innercentromereprotein,ARKbindingregion	1	1
TranscriptioninitiationfactorIID,18kDsubunit	3	3
Leucinecarboxylmethyltransferase	1	1
Beta-glucosidase(SUNfamily)	3	3
MitochondrialK+-H+exchange-related	1	1
Domainofunknownfunction(DUF4149)	1	1
SOR/SNZfamily	1	1
Phosphatidylinositol-4-phosphate5-Kinase	1	1
Nucleosidediphosphatekinase	1	1
MitochondrialimportproteinPam17	1	1
RibosomebiogenesisproteinSLX9	1	1
SMODSandSLOG-associating2TMeffectordomain	1	1
zinc-RINGfingerdomain	1	1
Spo7-likeprotein	1	1
Inositolmonophosphatasefamily	3	3
DehydroquinaseclassII	1	1
Myb/SANT-likeDNA-bindingdomain	1	1
U4/U6.U5smallnuclearribonucleoproteins	1	1
Acyl-coenzymeA:6-aminopenicillanicacidacyl-transferase	1	1
Whi5like	1	1
Thi4family	1	1
MitochondrialF1-F0ATPsynthasesubunitFoffungi	1	1
ETCcomplexIsubunitconservedregion	1	1
RibosomalproteinS19e	1	1
Universalstressproteinfamily	1	1
MBOAT,membrane-boundO-acyltransferasefamily	5	5
CDP-alcoholphosphatidyltransferase	3	3
Gti1/Pac2family	3	3

ARD/ARD'family	1	1
Proline-richnuclearreceptorcoactivatormotif	1	1
Fructose-bisphosphatealdolaseclass-II	7	7
Mediatorcomplexprotein	1	1
Clpprotease	1	1
Lipid-dropletassociatedhydrolase	1	1
TypeIIBDNAtopoisomerase	1	1
Dcp1-likedecappingfamily	1	1
AmmoniumTransporterFamily	5	5
BAGdomain	1	1
Proteinofunknownfunction(DUF1348)	1	1
LamB/YcsFfamily	1	1
Septationproteinetd1	1	1
Ornithinecyclodeaminase/mu-crystallinfamily	1	1
Ubiquinol-cytochromeCreductasehingeprotein	1	1
CyclinMtransmembraneN-terminaldomain	1	1
Fungalpotassiumchannel	1	1
Cytidylyltransferase-like	3	3
CHYzincfinger	3	3
Proteinofunknownfunction(DUF2457)	1	1
ATPsynthase,Delta/Epsilonchain,beta-sandwichdomain	1	1
TATA-bindingproteininteracting(TIP20)	1	1
DNA/RNAnon-specificendonuclease	1	1
Apc13pprotein	1	1
Uncharacterisedprotein(DUF2406)	1	1
RhoGEFdomain	1	1
RTC4-likedomain	1	1
ThiSfamily	1	1
HITzincfinger	1	1
RibosomalproteinL36e	1	1

CIDdomain	1	1
Yos1-like	1	1
Proteinphosphatase2AregulatoryBsubunit(B56family)	1	1
Proteinofunknownfunction(DUF962)	1	1
PWWPdomain	1	1
DHHCpalmitoyltransferase	1	1
ChromatinremodellingcomplexRsc7/Swp82subunit	3	3
Proteinofunknownfunction(DUF1765)	1	1
RibosomalproteinL11methyltransferase(PrmA)	1	1
Dualspecificityphosphatase,catalyticdomain	7	7
CPL(NUC119)domain	1	1
Triose-phosphateTransporterfamily	1	1
Fungaldomainofunknownfunction(DUF1746)	1	1
Bromodomainassociated	1	1
HistoneacetyltransferasesubunitNuA4	1	1
PWIdomain	1	1
Carbohydratekinase	1	1
Phosphomethylpyrimidinekinase	1	1
Mysoin-bindingmotifofperoxisomes	1	1
RibosomalproteinL34	1	1
VitaminB6photo-protectionandhomoeostasis	1	1
Mediatorcomplexsubunit16	1	1
Proteinofunknownfunction(DUF1674)	1	1
HistonedeacetylationproteinRxt3	1	1
60sAcidicribosomalprotein	1	1
D-Tyr-tRNA(Tyr)deacylase	1	1
SAC3/GANPfamily	1	1
SisterchromatidcohesionproteinDcc1	1	1
RHOproteinGDPdissociationinhibitor	1	1
ExonucleaseV-a5'deoxyribonuclease	1	1

FKBP-typepeptidyl-prolylcis-transisomerase	1	1
C2domain	1	1
Nitronatemonooxygenase	3	3
Tht1-likenuclearfusionprotein	1	1
ComplexIintermediate-associatedprotein30(CIA30)	1	1
Mannosyltransferase(PIG-V)	1	1
Proteinofunknownfunction(DUF2433)	1	1
Fungalhydrophobin	4	4
RNAligase	3	3
Domainofunknownfunction(DUF4291)	1	1
Proteinofunknownfunction(DUF3112)	1	1
GPR1/FUN34/yaaHfamily	5	5
TrypsinInhibitorlikecysteinerichdomain	1	1
SISdomain	3	3
MRC1-likedomain	1	1
UbiquinonebiosynthesisproteinCOQ7	3	3
DNAreplicationfactorCdt1C-terminaldomain	1	1
GlycogenrecognitionsiteofAMP-activatedproteinkinase	1	1
RibosomalproteinL22p/L17e	1	1
Serinehydroxymethyltransferase	1	1
Ribosomalprotein60SL18and50SL18e	1	1
DDHDdomain	1	1
SOSresponseassociatedpeptidase(SRAP)	1	1
Down-regulatedinmetastasis	1	1
PeptidaseM50B-like	1	1
DNApolymerasephi	1	1
Rad9	1	1
Prephenatedehydrogenase	1	1
Atg29N-terminaldomain	1	1
PXdomain	3	3

N-acetyltransferase	7	7
Rogdileucinezippercontainingprotein	1	1
Bacterialextracellularsolute-bindingproteins,family5Middle	1	1
Fumble	1	1
TranscriptionfactorPcc1	1	1
3'-5'exonuclease	5	5
Ricin-typebeta-trefoillectindomain-like	1	1
MIOREXcomplexcomponent7	1	1
Prefoldinsubunit	5	5
SigmadomainonNACHT-NTPases	1	1
RibosomalproteinS26e	1	1
Gcd10pfamily	1	1
Chorismatesynthase	1	1
MitochondrialribosomalproteinS25	1	1
CytochromecoxidasesubunitVIIc	1	1
Ima1N-terminaldomain	1	1
GYFdomain	1	1
RNAmethyltransferase	7	7
Cytidylyltransferasefamily	1	1
Proteinofunknownfunction(DUF3636)	1	1
RibonucleaseH2non-catalyticsubunit(Ylr154p-like)	1	1
TubulinbindingcofactorA	1	1
RibosomalproteinL24e	1	1
MA3domain	1	1
Interferon-induced6-16family	1	1
Legume-likelectinfamily	1	1
PLD-likedomain	3	3
Sulphurtransport	1	1
EisosomecomponentPIL1	1	1
Inorganicpyrophosphatase	1	1

MT-A70	1	1
Cytochromeoxidasecassembly	1	1
RibosomalproteinL13e	1	1
Pam16	1	1
MitochondrialgenomemaintenanceMGM101	1	1
TB2/DP1,HVA22family	1	1
Sec1-bindingregionofMso1	1	1
Calreticulinfamily	1	1
Cenp-Okinetochorecentromerecomponent	1	1
EssentialproteinYae1,Nterminal	1	1
Mitochondrialinner-membrane-boundregulator	1	1
Cofilin/tropomyosin-typeactin-bindingprotein	3	3
HITdomain	1	1
Etoposide-inducedprotein2.4(EI24)	1	1
Protoglobin	1	1
Arylesterase	3	3
DAHPsynthetaseIfamily	1	1
EMG1/NEP1methyltransferase	1	1
ConservedoligomericcomplexCOG6	1	1
AutophagyproteinATG9	1	1
SGT1protein	1	1
Lecithin:cholesterolacyltransferase	1	1
Proteinofunknownfunction(DUF2013)	1	1
Gar1/Naf1RNAbindingregion	1	1
ATPsynthasecomplexsubunith	1	1
CP2transcriptionfactor	1	1
EukaryotictranslationinitiationfactoreIF2A	1	1
Retrotransposongagprotein	1	1
Nucleotide-sugartransporter	1	1
OsmC-likeprotein	1	1

AAA-ATPaseVps4-associatedprotein1	1	1
HistonechaperoneRttp106-like	1	1
ArgininosuccinatelyaseC-terminal	1	1
Proteinofunknownfunction(DUF1264)	1	1
MeiosisproteinSPO22/ZIP4like	1	1
NLIinteractingfactor-likephosphatase	1	1
MediatorofRNApolymeraseIItranscriptioncomplexsubunit8	1	1
Acylphosphatase	1	1
RibosomalproteinS13/S18	1	1
Hepatocellularcarcinoma-associatedantigen59	1	1
Transcriptionalregulator	1	1
Alg9-likemannosyltransferasefamily	1	1
Putativedeath-receptorfusionprotein(DUF2428)	1	1
Proteinofunknownfunction(DUF3074)	1	1
AutophagyreceptorATG43	1	1
ChaC-likeprotein	1	1
Proteinofunknownfunction(DUF1223)	1	1
Nitroreductasefamily	1	1
AcidPhosphatase	1	1
PHdomain	1	1
Phosphatidatecytidylyltransferase,mitochondrial	1	1
ATPsynthaseEchain	1	1
MitochondrialribosomalproteinL27	1	1
Utp14protein	1	1
HECT-likeUbiquitin-conjugatingenzyme(E2)-binding	1	1
WH2motif	1	1
MitochondrialribosomalproteinL37	1	1
PeptidasefamilyM49	1	1
Inosine-uridinepreferringnucleosidehydrolase	1	1
Mitochondrialpyruvatecarriers	1	1

MitochondrialribosomalproteinL51/S25/CI-B8domain	1	1
Proteinofunknownfunction(DUF1687)	1	1
RibosomalproteinL14	1	1
ATPsynthaseDchain,mitochondrial(ATP5H)	1	1
PhosphatidylinositolN-acetylglucosaminyltransferase	1	1
SOH1	1	1
Thaumatinfamily	1	1
Domainofunknownfunction(DUF4336)	1	1
Salttolerancedown-regulator	1	1
PGAP1-likeprotein	1	1
Nucleolarprotein,Nop52	1	1
L-lysine6-monooxygenase(NADPH-requiring)	3	3
RibosomalproteinS19	3	3
6-O-methylguanineDNAmethyltransferase,DNAbindingdomain	1	1
ERCC4domain	1	1
Semialdehydedehydrogenase,NADbindingdomain	1	1
Phosphoglucomutase/phosphomannomutase,alpha/beta/alphadomainI	1	1
Cadmiumresistancetransporter	1	1
ExocystcomplexcomponentSec6	1	1
RibonucleaseP40kDa(Rpp40)subunit	1	1
Tim44-likedomain	1	1
RibosomalproteinS7p/S5e	1	1
PASdomain	3	3
Sodium:dicarboxylatesymporterfamily	1	1
Leucinerichrepeat	1	1
Transcriptionalactivatorofglycolyticenzymes	1	5
Fungalchitosanaseofglycosylhydrolasegroup75	1	1
RNApolymeraseIIIRPC4	1	1
N-terminaldomainonNACHT_NTPaseandP-loopNTPases	2	2
Phospholipase_D-nucleaseN-terminal	1	1

Mature-T-CellProliferationItype	1	1
Eukaryotictranslationinitiationfactor3subunit7(eIF-3)	1	1
Got1/Sft2-likefamily	1	1
Acetokinasefamily	1	1
HMG-boxdomain	1	1
O-methyltransferase	29	35
Condensincomplexsubunit2	1	1
Memo-likeprotein	1	1
Ssu72-likeprotein	1	1
AhpC/TSAfamily	1	1
Solutecarrierfamily35	1	1
CLN3protein	1	1
Ribosomebiogenesisregulatoryprotein(RRS1)	1	1
Mitochondrialribosomaldeath-associatedprotein3	1	1
RepairproteinRad1/Rec1/Rad17	1	1
Eukaryoticinitiationfactor4E	1	1
TRAPPtraffickingsubunitTrs65	1	1
DNAreplicationregulatorSLD3	1	1
BNRrepeat-containingfamilymember	1	1
RapidALkalinizationFactor(RALF)	1	1
Pyroglutamylpeptidase	1	1
DADfamily	1	1
GAFdomain	1	1
SANT/Myb-likedomainofDAMP1	1	1
Pentatricopeptiderepeatdomain	1	1
SUZdomain	1	1
Utp25,U3smallnucleolarRNA-associatedSSUprocessomeprotein25	1	1
Alteredinheritanceofmitochondria5	1	1
DomainfoundinIF2B/IF5	1	1
Glycosyltransferasefamily28C-terminaldomain	1	0

Uncharacterizedconservedprotein(DUF2183)	3	3
CoproporphyrinogenIIIoxidase	1	1
HOOKdomain	1	1
LYAR-typeC2HCzincfinger	1	1
R3Hdomain	1	1
KaiC	1	1
Domainofunknownfunction(DUF202)	3	3
Ubiquitin-2likeRad60SUMO-like	1	1
NMD3family	1	1
16SrRNAmethyltransferaseRsmB/F	3	3
LETM1-likeprotein	1	1
PeptidasefamilyC69	1	1
Vacuolarproteinsorting38	1	1
Guanylatekinase	1	1
Fattyacidhydroxylasesuperfamily	3	3
EXSfamily	1	1
Ppx/GppAphosphatasefamily	1	1
3-hydroxyanthranilicaciddioxygenase	1	1
YT521-B-likedomain	1	1
NucleoporinNup120/160	1	1
ATPsynthaseregulationproteinNCA2	1	1
Pterin4alphacarbinolaminedehydratase	1	1
Dpy-30motif	1	1
CatalyticLigBsubunitofaromaticring-openingdioxygenase	3	3
NADHubiquinoneoxidoreductasesubunitNDUFA12	3	3
Calponinhomology(CH)domain	1	1
Chitinrecognitionprotein	1	1
Glycosylhydrolasefamily62	1	1
3-dehydroquinatesynthase	1	1
Chromatinmodification-relatedproteinEAF7	1	1

RING-typezinc-finger	1	1
RNApolymerasesM/15Kdsubunit	1	1
Surfeitlocusprotein5subunit22ofMediatorcomplex	1	1
Proteinofunknownfunction(DUF3431)	1	1
Histoneacetylationprotein	1	1
Alb1	1	1
Signalpeptidase,peptidaseS26	1	1
ReplicationfactorAprotein3	1	1
RibosomalproteinL4/L1family	1	1
Domainofunknownfunction(DUF4188)	1	1
Ribonuclease-III-like	1	1
Domainofunknownfunction(DUF4048)	1	1
TranscriptionfactorOpi1	1	1
CyclinC-terminaldomain	1	1
Syntaxin6,N-terminal	1	1
Domainofunknownfunction(DUF1857)	1	1
CDC45-likeprotein	1	1
ExocystcomplexcomponentSec5	1	1
mRNAcappingenzyme,betachain	1	1
PeptidaseC65Otubain	1	1
Isy1-likesplicingfamily	1	1
Bacterialproteinofunknownfunction(DUF885)	1	0
UncharacterisedACR,YagEfamilyCOG1723	1	1
KinetochoreproteinMis14like	1	1
VesiclecoatproteininvolvedinGolgitoplasmamembranetransport	1	1
DSBA-likethioredoxindomain	1	1
betasubunitofN-acylethanolamine-hydrolyzingacidamidase	1	1
Bacteriocin-protection,YdeIorOmpD-Associated	1	1
YhhNfamily	1	1
Proteinofunknownfunction(DUF1295)	1	1

Macrodomain	1	1
Stretch-activatedCa2+-permeablechannelcomponent	1	1
Eisosomeprotein1	1	1
AdaptinNterminalregion	1	1
Single-strandbindingproteinfamily	1	1
PheromoneAreceptor	1	1
Sterol-sensingdomainofSREBPcleavage-activation	1	1
B-blockbindingsubunitofTFIIIC	1	1
GolginsubfamilyAmember7/ERF4family	1	1
RibosomalproteinL1p/L10efamily	1	1
MinimalbindingmotifofHap4forbindingtoHap2/3/5	1	1
SBF-likeCPAtransporterfamily(DUF4137)	1	1
RibosomalproteinS21	1	1
Capsularpolysaccharidesynthesisprotein	1	1
Prolineracemase	1	1
N2227-likeprotein	1	1
Eukaryoticmembraneproteinfamily	1	1
U1zincfinger	1	1
Integrasezincbindingdomain	1	0
TFIIICsubunittriplebarreldomain	1	1
Ornithinedecarboxylaseantizyme	1	1
INO80complexsubunitIes4	1	1
ThiopurineS-methyltransferase(TPMT)	1	1
OTU-likecysteineprotease	1	1
Hexapeptiderepeatofsuccinyl-transferase	1	1
PeptidasefamilyS58	1	1
PPIC-typePPIASEdomain	1	1
tRNApseudouridinesynthaseD(TruD)	1	1
Copper/zincsuperoxidedismutase(SODC)	1	0
Uncharacterisedproteinfamily(UPF0139)	1	1

BacterialIg-likedomain(group3)	1	1
Bucentaurorcraniofacialdevelopment	1	1
Domainofunknownfunction(DUF4602)	1	1
ESCRT-IIcomplexsubunit	1	1
Nucleosidetransporter	1	1
Cytochromec/c1hemelyase	1	1
BUD22	1	1
Integralperoxisomalmembraneperoxin	1	1
Acyltransferase	9	7
Lungseventransmembranereceptor	1	1
PutativeS-adenosyl-L-methionine-dependentmethyltransferase	1	1
Haspinlikekinasedomain	1	1
CytoplasmictRNA2-thiolationprotein2	1	1
pinin/SDK/memA/proteinconservedregion	1	1
ThreonylandAlanyltRNAsynthetasesecondadditionaldomain	3	3
ElectrontransferflavoproteinFAD-bindingdomain	1	1
39SmitochondrialribosomalproteinL46	1	1
U3snoRNAassociated	1	1
Nucleoside2-deoxyribosyltransferaselike	1	1
4Fe-4Sdiclusterdomain	1	1
MybDNA-bindinglike	1	1
Ribosomalproteins50S-L15,50S-L18e,60S-L27A	1	1
Mtf2family	1	1
CytochromecoxidasebiogenesisproteinCmc1like	1	1
Membrane-associatingdomain	1	1
POTfamily	5	5
Orotidine5'-phosphatedecarboxylase/HUMPSfamily	1	1
Carbohydrate/starch-bindingmodule(family21)	1	1
Lyticpolysaccharidemono-oxygenase,cellulose-degrading	1	1
High-affinitynickel-transportprotein	1	1

RmlDsubstratebindingdomain	1	1
Glyoxalase-likedomain	3	3
PBPsuperfamilydomain	1	1
Glyceratekinasefamily	1	1
Mus7/MMS22family	1	1
Pex14N-terminaldomain	1	1
PAPA-1-likeconservedregion	1	1
Proteinofunknownfunction(DUF4050)	1	1
Gon7family	1	1
HbrB-like	1	1
LIM-domainbindingprotein	1	1
EMC6	1	1
PHD-finger	3	3
UAAtransporterfamily	3	3
AMPdeaminase	1	1
Putativepeptidasefamily	1	1
TatDrelatedDNase	1	1
RNApolymeraseRpb3/Rpb11dimerisationdomain	1	1
Phosphatidylethanolamine-bindingprotein	1	1
LVIVDrepeat	1	1
Glycosylhydrolasesfamily17	1	1
MAC/Perforindomain	1	1
Domainofunknownfunction(DUF4604)	1	1
CytochromecoxidasesubunitVb	1	1
GCN5-likeprotein1(GCN5L1)	1	1
Ctf8	1	1
Putativediphthamidesynthesisprotein	1	1
DASHcomplexsubunitDad3	1	1
Sugar-tranasporters,12TM	1	1
Polyprenylsynthetase	1	1

CorehistoneH2A/H2B/H3/H4	1	1
UbiquitinfusiondegradationproteinUFD1	1	1
Proteinofunknownfunction(DUF726)	1	1
EutQ-likecupindomain	1	5
PRA1familyprotein	1	1
Aminoglycoside3-N-acetyltransferase	1	1
Arsenicalpumpmembraneprotein	1	1
Glucosamine-6-phosphateisomerases/6-phosphogluconolactonase	1	1
Diphthamidesynthase	1	1
Squalene/phytoenesynthase	1	1
TranscriptionfactorIwr1	1	1
MINDYdeubiquitinase	0	1
GlucoseinhibiteddivisionproteinA	0	1
Aromaticacidexporterfamilymember2	0	1
MOSCN-terminalbetabarreldomain	0	1
ABC-2familytransporterprotein	0	1
Proteinofunknownfunction(DUF1682)	0	1
Zinc-fingerofC2H2type	0	1
BEDzincfinger	0	3
Thymidylatesynthase	0	1
HypervirulenceassociatedproteinsTUDORdomain	0	1
Bladdercancer-relatedproteinBC10	0	1
PeptidaseC26	0	1

Supplementary data 2-Table 4 : predicted unique-gene families of *Fusarium oxysporum*. f. sp *albedinis* strains.		
**Gene families**	***Fusarium oxysporum*. f. sp *albedinis* 133.**	***Fusarium oxysporum*. f. sp *albedinis* 9**
	**Gene count**	**Gene count**
Prion-inhibitionandpropagation	1	0
FungalZn(2)-Cys(6)binuclearclusterdomain	1	24
FADbindingdomain	3	13
Asparaginesynthase	1	0
AMP-bindingenzyme	1	1
Fungalspecifictranscriptionfactordomain	19	41
Ankyrinrepeats(3copies)	15	43
DDEsuperfamilyendonuclease	41	273
Chromoshadowdomain	1	0
FMN-dependentdehydrogenase	1	7
AuxiliaryActivityfamily9(formerlyGH61)	1	1
RegulatorofGproteinsignalingdomain	1	1
hATfamilyC-terminaldimerisationregion	21	334
Possiblelysinedecarboxylase	1	0
Responseregulatorreceiverdomain	1	0
Transcriptionalactivatorofglycolyticenzymes	1	3
WGRdomain	5	1
MOSCdomain	1	0
CentromereDNA-bindingproteincomplexCBF3subunit,domain2	1	31
Beta-lactamase	5	9
WDdomain,G-betarepeat	1	9
NACHTdomain	1	9
Enoyl-CoAhydratase/isomerase	1	0
Sulfatepermeasefamily	1	5
Chromatetransporter	1	3

Multicopperoxidase	1	0
Tetratricopeptiderepeat	1	9
Proteinofunknownfunction(DUF3638)	1	0
Cyclin,N-terminaldomain	1	1
Reversetranscriptase(RNA-dependentDNApolymerase)	13	160
Calcium-dependentchannel,7TMregion,putativephosphate	1	5
Sodium/calciumexchangerprotein	1	5
Tc5transposaseDNA-bindingdomain	5	43
shortchaindehydrogenase	3	5
TransposaseIS4	19	85
LysMdomain	3	0
Sugar(andother)transporter	3	9
Heterokaryonincompatibilityprotein(HET)	3	21
MajorFacilitatorSuperfamily	9	9
Domainofunknownfunction(DUF5071)	1	0
Orsellinicacid/F9775biosynthesisclusterproteinD	3	25
WSCdomain	1	3
AIG1family	1	1
CytochromeP450	9	25
Fungalproteinkinase	1	1
alpha/betahydrolasefold	3	1
Whi5like	1	1
GlutathioneS-transferase,C-terminaldomain	1	1
Proteinofunknownfunction(DUF1593)	1	0
RNaseH	10	23
Glycosylhydrolasesfamily18	3	7
CHATdomain	1	11
Cellulase(glycosylhydrolasefamily5)	1	1
Flavin-bindingmonooxygenase-like	1	5
NADH:flavinoxidoreductase/NADHoxidasefamily	1	3

Ulp1proteasefamily,C-terminalcatalyticdomain	1	1
Phosphatidylserinedecarboxylase	1	0
MAC/Perforindomain	1	1
Proteinofunknownfunction(DUF4238)	1	0
Glyoxalase/Bleomycinresistanceprotein/Dioxygenasesuperfamily	1	0
Methyltransferasedomain	1	5
CFEMdomain	3	1
Phosphotransferaseenzymefamily	3	0
Berberineandberberinelike	1	1
HNHendonuclease	1	1
Serinecarboxypeptidase	1	1
PIF1-likehelicase	1	17
Acetyltransferase(GNAT)family	1	0
NADdependentepimerase/dehydratasefamily	1	0
DnaJdomain	3	3
Amino-transferaseclassIV	1	0
Cationtransportprotein	1	0
Basicregionleucinezipper	1	0
GAG-pre-integrasedomain	1	1
Fumarylacetoacetate(FAA)hydrolasefamily	1	1
Commoncentraldomainoftyrosinase	1	0
NmrA-likefamily	1	17
Cysteine-richsecretoryproteinfamily	1	3
Permeaseforcytosine/purines,uracil,thiamine,allantoin	3	1
Retrotransposongagprotein	1	1
FAR1DNA-bindingdomain	1	0
BRCA1CTerminus(BRCT)domain	1	0
Helitronhelicase-likedomainatN-terminus	1	99
Endonuclease-reversetranscriptase	1	19
Tubulin-tyrosineligasefamily	1	0

Poly(ADP-ribose)polymerasecatalyticdomain	3	7
DEAD/DEAHboxhelicase	1	5
Uncharacterizedalpha/betahydrolasedomain(DUF2235)	1	7
Acetyltransferase(GNAT)domain	1	0
RNA3'-terminalphosphatecyclase	1	0
GLEYAdomain	1	0
Aminoacidpermease	1	3
L-asparaginaseII	1	0
Sodium:neurotransmittersymporterfamily	0	1
Alpha-acetolactatedecarboxylase	0	1
RecQzinc-binding	0	3
Histidinekinase-,DNAgyraseB-,andHSP90-likeATPase	0	5
Glycosylhydrolasefamily1	0	1
MULEtransposasedomain	0	86
Bul1Cterminus	0	1
Bromodomain	0	3
E1-E2ATPase	0	1
Mis12-Mtw1proteinfamily	0	3
N-terminaldomainofNWDNACHT-NTPase	0	7
Melibiase	0	1
GlutathioneS-transferase,N-terminaldomain	0	1
PEP-utilisingenzyme,PEP-bindingdomain	0	1
RNaseH-likedomainfoundinreversetranscriptase	0	15
Putativelumazine-binding	0	1
CoAbindingdomain	0	1
Glycosylhydrolasefamily3C-terminaldomain	0	5
Subtilasefamily	0	1
Dihydrodipicolinatesynthetasefamily	0	1
AlcoholdehydrogenaseGroES-likedomain	0	1
Ringfingerdomain	0	1

Chitinsynthase	0	1
Matrixin	0	1
AcyclicterpeneutilisationfamilyproteinAtuA	0	1
tRNAsynthetasesclassII(D,KandN)	0	1
Dak1domain	0	1
Zincfinger,C2H2type	0	5
Aldehydedehydrogenasefamily	0	7
GMCoxidoreductase	0	1
HelicaseconservedC-terminaldomain	0	5
Sulfotransferasedomain	0	3
Integrasezincbindingdomain	0	1
Trypsin-likepeptidasedomain	0	5
Middleorthirddomainofpeptidase_M16	0	1
Proteinofunknownfunction(DUF3435)	0	1
Proteinkinasedomain	0	19
Proteinofunknownfunction(DUF659)	0	17
Proteinofunknownfunction(DUF4246)	0	3
Putativeserinedehydratasedomain	0	1
Regulatorofchromosomecondensation(RCC1)repeat	0	1
ScaffoldproteinNfu/NifUNterminal	0	1
AdoMetdependentprolinedi-methyltransferase	0	1
Proteinofunknownfunction(DUF1275)	0	1
CoenzymeAtransferase	0	1
CoA-transferasefamilyIII	0	1
PutativeFMN-bindingdomain	0	1
Ubiquitincarboxyl-terminalhydrolase	0	1
26SproteasomesubunitRPN7	0	1
ABC-2typetransporter	0	1
Glycosyltransferasefamily28N-terminaldomain	0	1
Domainofunknownfunction(DUF1989)	0	1

ABCtransporter	0	3
Alphaamylase,catalyticdomain	0	1
Frequencyclockprotein	0	5
TctoxincomplexTcAC-terminalTcB-bindingdomain	0	1
Caspasedomain	0	3
bZIPtranscriptionfactor	0	3
Phosphorylasesuperfamily	0	3
ABCtransportertransmembraneregion	0	1
Las17-bindingproteinactinregulator	0	1
Egh16-likevirulencefactor	0	1
3-oxo-5-alpha-steroid4-dehydrogenase	0	1
TCP-1/cpn60chaperoninfamily	0	1
Peroxidase,family2	0	1
GDSL-likeLipase/Acylhydrolase	0	1
RTA1likeprotein	0	1
Amidohydrolase	0	1
MYNDfinger	0	1
Proteinofunknownfunction(DUF2985)	0	1
Patatin-likephospholipase	0	5
Chromo(CHRromatinOrganisationMOdifier)domain	0	5
Pleckstrinhomologydomain	0	3
ER-boundoxygenasempaB/B'/Rubberoxygenase,catalyticdomain	0	1
EsteraseFrsA-like	0	1
BacterialsignallingproteinNterminalrepeat	0	1
helix-turn-helix,Psqdomain	0	7
3-betahydroxysteroiddehydrogenase/isomerasefamily	0	3
Domainofunknownfunction(DUF1996)	0	3
Beta-lactamasesuperfamilydomain	0	3
Proteinofunknownfunction(DUF3533)	0	1
BTB/POZdomain	0	1

Proteinofunknownfunction(DUF3716)	0	9
Beta-ketoacylsynthase,N-terminaldomain	0	1
Spherulation-specificfamily4	0	1
Fungalproteinofunknownfunction(DUF1752)	0	5
Aldo/ketoreductasefamily	0	1
Reticulon	0	1
GATAzincfinger	0	1
Alginatelyase	0	1
HSF-typeDNA-binding	0	1
Ca2+regulatorandmembranefusionproteinFig1	0	1
Dienelactonehydrolasefamily	0	3
HpcH/HpaIaldolase/citratelyasefamily	0	1
Integrasecoredomain	0	1
Sulfatase	0	1
Glycosylhydrolasesfamily43	0	1
Glycosylhydrolasesfamily28	0	2
SurvivalproteinSurE	0	1
Myb-likeDNA-bindingdomain	0	1
Proteinofunknownfunction(DUF3429)	0	1
Zincknuckle	0	1
OTT_1508-likedeaminase	0	1
BacteriallowtemperaturerequirementAprotein(LtrA)	0	1
UV-endonucleaseUvdE	0	1
FtsJ-likemethyltransferase	0	1
Proteinphosphatase2AregulatoryBsubunit(B56family)	0	1
Conservedhypotheticalprotein(DUF2461)	0	1
Pyridinenucleotide-disulphideoxidoreductase	0	3
EutQ-likecupindomain	0	1
FADdependentoxidoreductase	0	1
BEDzincfinger	0	1

Eukaryoticproteinofunknownfunction(DUF829)	0	3
Glycosylhydrolasefamily45	0	1
vonWillebrandfactortypeAdomain	0	1
Proteinofunknownfunction(DUF2722)	0	1
Up-regulatedDuringSeptation	0	1
Amidase	0	1
Meioticallyup-regulatedgenefamily	0	3
Enoyl-(Acylcarrierprotein)reductase	0	1
Extracellulartail,of10TMputativephosphatetransporter	0	1
NAD(P)-bindingRossmann-likedomain	0	1
Proteinofunknownfunction(DUF3455)	0	1
PAP2superfamily	0	1
Linalooldehydratase/isomerase	0	1
Ferricreductaseliketransmembranecomponent	0	1
PutativeERtransporter,6TM,N-terminal	0	1
Eukaryoticaspartylprotease	0	1
AminotransferaseclassIandII	0	1
Glycosylhydrolasefamily3Nterminaldomain	0	1
Familyofunknownfunction(DUF6060)	0	1
SnoaL-likedomain	0	1
OxidoreductaseNAD-bindingdomain	0	1
Globin	0	1
Glycosylhydrolasesfamily32Cterminal	0	1
