# Supplementary material for: In silico comparative genomic analysis unravels a new candidate protein arsenal specifically associated with Fusarium oxysporum f. sp. albedinis pathogenesis
Source: Sci Rep. 2022 Nov 9;12:19098. doi: 10.1038/s41598-022-21858-1 (PMC9646873; doi:10.1038/s41598-022-21858-1)
Supplement: Supplementary file 3 — Supplementary Information 3. [file 41598_2022_21858_MOESM3_ESM.pdf]

Supplementary data 3-Table 1 : predicted secreted effectors of the analyzed formea speciales belonging to Fusarium oxysporum species complex.

Stains	Core-secretome				accessory-secretome				Unique-secretome			
	Cytoplasmic effectors withe Unknown Function	Cytoplasmic effectors withe known Function	Apoplastic effectors withe Unknown Function	Apoplastic effectors withe known Function	Cytoplasmic effectors withe Unknown Function	Cytoplasmic effectors withe known Function	Apoplastic effectors withe Unknown Function	Apoplastic effectors withe known Function	Cytoplasmic effectors withe Unknown Function	Cytoplasmic effectors withe known Function	Apoplastic effectors withe Unknown Function	Apoplastic effectors withe known Function
F.o.f.sp.apiiNRRL38295	162	547	31	49	777	608	215	101	37	33	7	3
F.o.f.sp.coriandrii3-2	154	536	31	51	770	623	232	106	2	0	11	2
F.o.f.sp.coriandriiGL306	160	543	34	54	797	636	231	102	5	1	1	0
F.o.f.sp.vasinfectumLA1E	152	534	32	49	610	508	174	88	73	75	13	11
F.o.f.sp.spinaciaeFus254	152	585	33	54	669	577	175	88	91	73	10	5
F.o.f.sp.momordicae90NF2-1	159	559	31	52	613	542	195	99	10	1	1	0
F.o.f.sp.momordicaeNRRL26413	139	514	35	50	639	561	196	92	4	1	0	23
F.o.f.sp.lagenariae01-03008	138	552	31	51	683	556	200	90	79	34	7	3
F.o.f.sp.fragariae160609	154	543	34	48	701	574	196	98	44	11	3	0
F.o.f.sp.niveumR1	153	556	35	54	526	494	189	97	120	168	25	15
F.o.f.sp.spinaciaeMF15	139	568	32	49	709	564	199	105	124	63	21	6
F.o.f.sp.nicotianaeFt-1512	159	528	37	49	607	505	182	98	68	22	7	4
F.o.f.sp.vasinfectumNRRL31665	163	612	34	52	620	529	178	90	100	59	16	7
F.o.f.sp.liliiFol39	152	574	34	43	612	538	181	93	100	50	19	9
F.o.f.sp.fragariaeBRIP5168a	162	542	35	52	507	462	168	89	52	14	9	2
F.o.f.sp.LagenariaeLag3-1	150	544	32	59	514	469	175	85	102	31	17	7
F.o.f.sp.koae44	159	538	33	47	401	399	138	79	83	20	13	7
F.o.f.sp.conglutinans58385	153	513	33	50	884	652	212	113	3	0	73	32
F.o.f.sp.conglutinansFGL03-6	153	510	36	54	765	664	174	105	45	4	3	0
F.o.f.sp.conglutinansrace254008	142	533	36	52	786	610	212	108	5	2	0	36
F.o.f.sp.melonisFom005	152	566	35	51	615	555	206	109	130	65	19	4
F.o.f.sp.niveumFon002	151	544	37	49	602	534	198	97	128	58	18	7
F.o.f.sp.cucumerinumFoc018	138	527	31	51	809	651	217	118	85	39	9	1
F.o.f.sp.cucumerinumFoc030	142	574	34	54	803	659	221	111	80	32	5	4
F.o.f.sp.Raphani54005	133	527	34	52	683	565	199	95	117	66	19	11
F.o.f.sp.MelongenaeJ-71	159	582	34	52	567	503	171	94	117	67	16	13
F.o.f.sp.pisiHDV247	159	520	33	54	668	552	184	113	104	63	10	8
F.o.f.sp.liniF282	145	598	33	54	540	470	158	85	30	24	9	4
F.o.f.sp.liniF324	157	547	37	58	525	468	157	95	43	27	10	3
F.o.f.sp.cubense160527	170	548	32	58	428	474	167	86	198	63	46	10
F.o.f.sp.ciceris38-1	146	541	29	46	437	490	153	67	338	126	57	23
F.o.f.sp.gladioliG76	130	533	28	54	598	504	178	93	257	142	34	19

F.o.f.sp.lycopersiciFol014	153	545	31	51	666	510	176	100	3	1	0	47
F.o.f.sp.lycopersiciFol074	142	546	31	51	711	581	184	111	26	8	2	0
F.o.f.sp.Melonis26406	137	545	30	52	628	579	192	101	125	106	32	8
F.o.f.sp.albedinis133	175	550	33	55	1120	639	208	129	71	18	3	2
F.o.f.sp.albedinis9	158	562	31	51	627	600	200	115	46	25	1	2
F.o.f.sp.matthiolaePHW726	155	512	33	47	723	572	174	94	76	21	5	0
F.o.f.sp.tulipaeTu67	151	526	32	49	696	546	164	105	103	71	16	5
F.o.f.sp.apii207	138	526	32	50	582	560	193	104	159	104	54	9
F.o.f.sp.radicis-cucumerinumForc024	150	535	36	50	568	508	190	102	11	2	3	0
F.o.f.sp.radicis-cucumerinumForc031	161	546	34	53	580	502	189	107	2	1	0	16
F.o.f.sp.gladioliG2	131	516	32	50	656	551	173	105	75	40	11	5
F.o.f.sp.cepaeFoC125	149	549	32	47	694	619	191	100	3	1	0	14
F.o.f.sp.cepaeFoCFus2	134	537	31	55	603	627	196	97	6	1	0	20
F.o.f.sp.medicaginisFom-5190a	146	541	34	51	552	514	181	101	107	54	14	4
F.o.f.sp.Radicis-lycopersici26381	157	535	33	48	549	456	172	98	112	50	14	7
F.o.f.sp.nicotianaeFt-Rob	150	528	29	50	533	492	169	98	84	31	10	2
F.o.f.sp.narcissiN139	142	560	30	49	738	668	209	111	93	54	6	2
F.o.f.sp.narcissiNa5	150	532	29	52	778	584	212	111	91	48	12	5
F.o.f.sp.cubenseC1HIR9889	174	579	35	60	334	359	135	73	251	109	57	12

Supplementary data 3-Table 2 : predicted effector families of *Fusarium oxysporum*.f. sp. *albedinis* strains		
**Effectors families**	***Fusarium oxysporum*. f. sp. *albedinis* 133**	***Fusarium oxysporum*. f. sp. *albedinis* 9**
	**Effector count**	**Effector count**
Thioredoxin	15	5
Righthandedbetahelixregion	5	11
Glycosylhydrolasesfamily43	15	13
Fasciclindomain	7	9
Subtilasefamily	12	13
Glucanosyltransferase	1	1
Thioredoxin-likedomain	1	1
DnaJCterminaldomain	1	17
GlutathioneS-transferase,N-terminaldomain	7	5
DnaJdomain	11	13
Tuberculosisnecrotizingtoxin	1	1
AuxiliaryActivityfamily9(formerlyGH61)	9	3
Lyticpolysaccharidemono-oxygenase,cellulose-degrading	3	1
Glycosylhydrolasesfamily11	5	7
Pregnancy-associatedplasmaprotein-A	3	28
Bacterialextracellularsolute-bindingprotein	3	17
Pectinesterase	8	14
S1/P1Nuclease	1	7
GlucosidaseIIbetasubunit-likeprotein	3	3
Autophagy-relatedprotein27	1	13
Glycosylhydrolasesfamily28	9	3
Pectatelyase	27	9
Glycosylhydrolasesfamily16	18	5
GDSL-likeLipase/Acylhydrolasefamily	13	1
Phosphoesterasefamily	1	8
Polysaccharidedeacetylase	7	6
Trypsin	3	1
Fungalhydrophobin	5	11
60sAcidicribosomalprotein	1	9
EsterasePHBdepolymerase	5	1
Asparaginase	3	3
emp24/gp25L/p24family/GOLD	7	5
Cerato-platanin	7	3
SUR7/PalIfamily	1	1
Necrosisinducingprotein(NPP1)	7	1
Pathogeneffector putativenecrosis-inducingfactor	3	3
FKBP-typepeptidyl-prolylcis-transisomerase	3	1

Hsp70protein	1	5
Lipase(class3)	1	19
Inorganicpyrophosphatase	1	1
PeptidasefamilyM28	1	1
CFEMdomain	9	5
Glycosyltransferasesugar-bindingregioncontainingDXDmotif	1	1
LicDfamily	3	1
Alternariaalternataallergen1	1	1
CHRDdomain	1	1
Cysteine-richsecretoryproteinfamily	18	1
ElectrontransferDM13	1	1
ribonuclease	17	3
Glycoside-hydrolasefamilyGH114	5	1
NucleotideexchangefactorSIL1	1	7
Histidinephosphatasesuperfamily(branch1)	5	1
Zinccarboxypeptidase	3	7
HydrophobicsurfacebindingproteinA	1	1
MLdomain	1	1
Glycerophosphoryldiesterphosphodiesterasefamily	1	1
WSCdomain	7	1
EndoplasmicReticulumOxidoreductin1(ERO1)	1	5
Histidinephosphatasesuperfamily(branch2)	1	3
Glycosylhydrolasefamily45	1	6
Alpha-L-arabinofuranosidaseB,catalytic	1	3
Glycosylhydrolasefamily7	3	1
LysMdomain	9	5
PANdomain	6	1
Putativenuclearenvelopeorganisationprotein	1	7
Platelet-activatingfactoracetylhydrolase,isoformII	1	1
Phagelysozyme	3	1
Cupin	17	1
Proteinofunknownfunction(DUF3455)	3	3
EthDdomain	5	3
Aldo/ketoreductasefamily	11	1
Glycosidehydrolase131catalyticN-terminaldomain	1	7
Glycosylhydrolasefamily12	7	1
Phospholipase/Carboxylesterase	1	1
Alginatelyase	1	1
ChorismatemutasetypeII	1	1
Astacin(PeptidasefamilyM12A)	5	1

Eukaryoticaspartylprotease	3	5
Familyofunknownfunction(DUF6060)	1	1
FADdependentoxidoreductase	3	1
Egh16-likevirulencefactor	7	1
Cutinase	5	1
Glycosylhydrolasefamily10	1	7
SMP-30/Gluconolactonase/LRE-likeregion	3	3
Proteinofunknownfunction(DUF3237)	5	1
ExpansinC-terminaldomain	1	3
Translocon-associatedprotein(TRAP),alphasubunit	1	1
CHAPdomain	1	9
Glycosylhydrolasesfamily18	7	3
Spt4/RpoE2zincfinger	1	1
Nis1family	1	1
Pro-kumamolisin,activationdomain	1	5
TrypsinInhibitorlikecysteinerichdomain	1	1
Calreticulinfamily	1	1
Dienelactonehydrolasefamily	5	4
Glycosyltransferasefamily17	1	1
Fungalchitosanaseofglycosylhydrolasegroup75	1	5
RapidALkalinizationFactor(RALF)	1	1
GLEYAdomain	1	5
Chitinrecognitionprotein	1	1
SurvivalproteinSurE	1	1
Glycosylhydrolasefamily62	1	0
Cyclophilintypepeptidyl-prolylcis-transisomerase/CLD	5	1
RibonucleaseT2family	1	1
PBPsuperfamilydomain	1	5
Rieske[2Fe-2S]domain	0	1
Beta-glucosidase(SUNfamily)	1	13
ProkaryoticphospholipaseA2	3	27
Phosphatidylethanolamine-bindingprotein	1	1
PeptidaseA4family	1	3
Tc5transposaseDNA-bindingdomain	24	17
hATfamilyC-terminaldimerisationregion	15	1
Nse4C-terminal	1	1
D-isomerspecific2-hydroxyaciddehydrogenase,NADbindingdomain	3	1
Ubiquitin-bindingdomain	1	1
C2HE/C2H2/C2HCzinc-bindingfinger	1	1
OxidoreductaseNAD-bindingdomain	3	1

Proteinprenyltransferasealphasubunitrepeat	1	1
Actincytoskeleton-regulatorycomplexproteinEND3	1	1
Zincfinger,C2H2type	13	1
RibosomalS13/S15N-terminaldomain	1	1
Synaptobrevin	3	3
PPIC-typePPIASEdomain	1	1
Cofilin/tropomyosin-typeactin-bindingprotein	7	1
UncharacterizedproteinfamilyUPF0029	1	1
EF-handdomainpair	7	1
Pre-mRNAsplicingfactor	1	1
Domainofunknownfunction(DUF4187)	1	1
RibosomalproteinL4/L1family	1	1
TFA2Wingedhelixdomain2	1	7
Velvetfactor	1	1
Signalpeptidasesubunit	1	1
3-hydroxyacyl-CoAdehydrogenase,NADbindingdomain	1	3
Cullinbinding	1	1
RibosomalproteinS5,C-terminaldomain	1	1
CSLzincfinger	3	1
SWIB/MDM2domain	1	1
MannitoldehydrogenaseC-terminaldomain	1	1
Ribosome-associatedcomplexheaddomain	1	3
Galactose-1-phosphateuridyltransferase,N-terminaldomain	1	1
2Fe-2Siron-sulfurclusterbindingdomain	1	1
RNApolymeraseIIIsubunitRpc25	1	1
CDK-activatingkinaseassemblyfactorMAT1	1	1
Ribosomal_S17N-terminal	1	7
JAB1/Mov34/MPN/PAD-1ubiquitinprotease	1	16
ATPasefamilyassociatedwithvariouscellularactivities(AAA)	7	1
Coiled-coildomaincontainingprotein(DUF2052)	1	1
Acyl-CoAdehydrogenase,C-terminaldomain	1	1
Zinc-binding	7	1
WDdomain,G-betarepeat	1	1
Multiproteinbridgingfactor1	1	7
Cytokine-inducedanti-apoptosisinhibitor1,Fe-Sbiogenesis	1	3
Aft1HRRdomain	1	3
bZIPtranscriptionfactor	16	1
RibosomalfamilyS4e	1	1
Ubiquitincarboxyl-terminalhydrolase	3	3
NucleotideexchangefactorFes1	1	1

Pyridoxine5'-phosphateoxidaseC-terminaldimerisationregion	1	1
ATPsynthasesubunitC	1	1
TranscriptioninitiationfactorIIA,gammasubunit	1	1
PP-loopfamily	1	5
Proteasomesubunit	1	1
RPELrepeat	1	1
Sof1-likedomain	1	5
2OG-Fe(II)oxygenasesuperfamily	9	1
Ubiquitin-conjugatingenzyme	22	1
Nucleoporincomplexsubunit54	1	7
RibosomalproteinL6	3	1
EB1-likeC-terminalmotif	1	1
RibosomalProteinsL2,C-terminaldomain	3	1
CHORD	1	1
ReplicationfactorCC-terminaldomain	1	1
Glutathione-dependentformaldehyde-activatingenzyme	15	1
IEC3subunitoftheIno80complex,chromatinre-modelling	1	1
AN1-likeZincfinger	3	1
Nuf2family	1	1
TranscriptionfactorTFIIBrepeat	1	3
F-boxonlyproteinC-terminalregion	1	1
TranscriptionfactorTFIIDcomplexsubunit8C-term	1	1
Uricase	1	7
NIPSNAP	1	1
Calcineurin-likephosphoesterase	3	1
CTDkinasesubunitgammaCTK3	1	1
DEAD/DEAHboxhelicase	3	1
Gelsolinrepeat	1	1
C-terminusofhistoneH2A	1	1
R3Hdomain	1	1
Rasfamily	25	1
SBDSproteinC-terminaldomain	1	1
Chromoshadowdomain	3	1
EF-1guaninenucleotideexchangedomain	1	1
2-oxoaciddehydrogenasesacyltransferase(catalyticdomain)	1	1
HSCBC-terminaloligomerisationdomain	1	1
RibosomalproteinL7Ae/L30e/S12e/Gadd45family	1	3
SWIRMdomain	1	1
Zinc-fingerdouble-strandedRNA-binding	3	3
Lowmolecularweightphosphotyrosineproteinphosphatase	1	1

Nucleotidyltransferase	1	1
Acetyltransferase(GNAT)family	35	1
BTB/POZdomain	15	1
DSS1/SEM1family	1	1
PXdomain	3	21
ARP2/3complex20kDasubunit(ARPC4)	1	1
Peroxidase,family2	5	1
Cupindomain	13	1
Nucleartransportfactor2(NTF2)domain	1	1
MafB19-likedeaminase	1	1
Shikimatekinase	1	1
YqcI/YcgGfamily	1	1
RibosomalproteinL21e	1	3
RNApolymeraseRpb8	1	1
ComplexIintermediate-associatedprotein30(CIA30)	1	1
Tim44-likedomain	1	9
NFACTproteinRNAbindingdomain	1	1
RibosomalproteinS7e	1	3
MYNDfinger	3	1
SURF1family	1	1
Domainofunknownfunction(DUF4452)	1	1
RibosomalS3Aefamily	1	3
Hptdomain	1	1
Grap2andcyclin-D-interacting	1	1
AKAP72'5'RNAligase-likedomain	1	1
Rtf2RING-finger	1	1
TranscriptionfactorS-II(TFIIS)	3	3
Sin3bindingregionofhistonedeacetylasecomplexsubunitSAP30	1	3
Familyofunknownfunction(DUF5308)	1	1
Proteolipidmembranepotentialmodulator	1	7
RibonucleaseIIIdomain	1	1
Heatshockfactorbindingprotein1	1	3
CobW/HypB/UreG,nucleotide-bindingdomain	1	1
Peptidyl-tRNAhydrolasePTH2	1	1
Pyoverdine/dityrosinebiosynthesisprotein	1	13
Autophagy-relatedprotein101	1	3
NADH-ubiquinoneoxidoreductaseB18subunit(NDUFB7)	1	3
Putativeamidoligaseenzyme	1	1
Histone-liketranscriptionfactor(CBF/NF-Y)andarchaealhistone	11	7
Ringhydroxylatingalphasubunit(catalyticdomain)	1	1

IGRproteinmotif	1	1
GlycinecleavageH-protein	3	1
Rtr1/RPAP2family	1	1
Putativeheavy-metal-binding	1	15
ERproteinPkr1	1	1
Alpha/betahydrolasefamily	5	11
Dephospho-CoAkinase	1	11
D-mannosebindinglectin	1	15
CSdomain	1	1
Helix-loop-helixDNA-bindingdomain	19	45
A49-likeRNApolymeraseIassociatedfactor	1	7
Familyofunknownfunction(DUF5572)	1	3
GDSL-likeLipase/Acylhydrolase	15	1
Tim10/DDPfamilyzincfinger	5	1
NAD(P)H-binding	3	1
SKIP/SNWdomain	1	1
RibosomalproteinS21e	1	5
RibosomalproteinL14p/L23e	3	1
shortchaindehydrogenase	32	1
Peptidemethioninesulfoxidereductase	1	1
Oxidoreductasefamily,NAD-bindingRossmannfold	7	1
Rhodanese-likedomain	7	1
RNApolymeraseIspecificinitiationfactor	1	1
ETCcomplexIsubunitconservedregion	3	1
TaurinecatabolismdioxygenaseTauD,TfdAfamily	3	24
Clathrinlightchain	1	11
Serineaminopeptidase,S33	1	5
RibosomalproteinL22p/L17e	3	1
RibosomalproteinL16p/L10e	3	1
ATPsynthasedelta(OSCP)subunit	1	1
Alanineracemase,N-terminaldomain	1	11
Ribosomerecyclingfactor	1	1
RibosomalproteinL36	3	3
RNApolymerasesN/8kDasubunit	1	1
RibosomalproteinS18	1	1
Lysinemethyltransferase	1	1
Interferon-relateddevelopmentalregulator(IFRD)	1	5
Cytochromeb5-likeHeme/Steroidbindingdomain	7	1
Cupinsuperfamily(DUF985)	1	1
TranscriptionalCoactivatorp15(PC4)	1	1

Mitochondriallargesubunitribosomalprotein(Img2)	1	1
RibosomalproteinL44	1	1
DSBA-likethioredoxindomain	1	1
PredictedSAM-dependentRNAmethyltransferase	1	1
25SrRNA(adenine(2142)-N(1))-methyltransferase,Bmt2	1	5
Fungalproteinofunknownfunction(DUF2015)	1	1
Mis12protein	1	1
Proteinofunknownfunction(DUF3767)	1	5
Myb-likeDNA-bindingdomain	9	1
tRNAsynthetasesclassI(WandY)	1	1
SAMdomain(Sterilealphamotif)	1	3
Thioesterase-likesuperfamily	5	1
NifU-likeNterminaldomain	1	1
HORMAdomain	1	1
Aldose1-epimerase	1	1
PETassemblyofcytochromecoxidase,mitochondrial	1	3
Vacuolar(H+)-ATPaseGsubunit	1	1
Uncharacterisedproteinfamily(UPF0203)	1	1
RNApolymeraseIItranscriptionmediatorcomplexsubunit9	1	1
CBP4	1	1
RibosomalproteinL24e	1	1
Redoxin	3	3
HDdomain	3	37
Copper/zincsuperoxidedismutase(SODC)	3	11
SPFHdomain/Band7family	1	3
Cnl2/NKP2familyprotein	1	7
Uncharacterizedconservedprotein	1	3
RibosomalproteinL30p/L7e	1	1
6-O-methylguanineDNAmethyltransferase,DNAbindingdomain	3	1
DNApolymerasedelta,subunit4	1	1
Prefoldinsubunit	5	3
Cwf15/Cwc15cellcyclecontrolprotein	1	1
Familyofunknownfunction(DUF5321)	1	1
RibosomalproteinS10p/S20e	1	1
MitochondrialribosomalproteinL31	1	1
Gamma-glutamylcyclotransferase,AIG2-like	3	9
Transcriptionalregulationofmitochondrialrecombination	1	1
HomeoboxKNdomain	1	1
RNasePsubunitPop3	1	28
RibosomalproteinS7p/S5e	3	1

Lowtemperatureviabilityprotein	1	3
GDP/GTPexchangefactorSec2p	1	1
CytochromecoxidasesubunitVa	1	1
NLIinteractingfactor-likephosphatase	1	1
RibosomalproteinS9/S16	1	1
Pyridoxamine5'-phosphateoxidaselike	1	1
Extracellularmutantprotein11	1	1
Large-conductancemechanosensitivechannel,MscL	1	1
Leucine-richrepeat	1	1
Non-classicalexportprotein1	1	1
Spc24subunitofNdc80	1	1
S4domain	1	1
EndoribonucleaseL-PSP	15	1
Lateendosomal/lysosomaladaptorandMAPKandMTORactivator	1	1
MED7protein	1	5
Proteinofunknownfunction(DUF1168)	1	1
RNAsePRpr2/Rpp21/SNM1subunitdomain	1	1
RibosomalproteinL31e	1	1
PCIdomain	1	1
RegulatorofVps4activityintheMVBpathway	1	1
Methyltransferasedomain	25	7
Shortcoiled-coilprotein	7	1
Initiationfactor2subunitfamily	1	1
SNOglutamineamidotransferasefamily	1	3
CTLH/CRAC-terminaltoLisHmotifdomain	1	1
CatalyticLigBsubunitofaromaticring-openingdioxygenase	1	1
Autophagyprotein16(ATG16)	1	1
Guanylatekinase	3	1
Thioesterasedomain	1	1
Peptidyl-tRNAhydrolase	3	1
Chalconeisomeraselike	1	1
RibosomalproteinL13	3	1
DJ-1/PfpIfamily	1	1
CHCHdomain	1	3
SCO1/SenC	1	1
Complex1_LYR-like	5	1
Pyridoxamine5'-phosphateoxidase	5	1
Sir2family	1	1
SelRdomain	1	1
Whi5like	3	1

TranscriptionfactorsubunitMed10ofMediatorcomplex	1	1
RNApolymeraseRpb6	1	1
MitochondrialribosomalproteinL51/S25/CI-B8domain	3	1
Domainofunknownfunction(DUF4748)	1	3
Proteinofunknownfunction(DUF3602)	5	1
Magobinding	1	1
RibosomalproteinS8e	1	1
ERCC4domain	1	15
RNApolymeraseRpb4	1	1
RNArecognitionmotif.(a.k.a.RRM,RBD,orRNPdomain)	11	1
G-patchdomain	3	1
Cytochromec/c1hemelyase	1	1
Nucleotide-diphospho-sugartransferase	1	1
Murligasemiddledomain	1	1
UBXdomain	1	1
MitochondrialribosomalproteinsubunitL20	1	1
Isocitrate/isopropylmalatedehydrogenase	1	3
R3H-associatedN-terminaldomain	1	1
Mitochondrial18KDaprotein(MTP18)	1	5
Translationinitiationfactor1A/IF-1	1	3
She9/Mdm33family	1	1
Sas10/Utp3/C1Dfamily	1	1
U-boxdomain	1	1
TIM21	1	3
Iron-sulfurclusterassemblyprotein	1	12
BindingdomainofDNArepairproteinErcc1(rad10/Swi10)	1	1
DehydrogenaseE1component	1	1
Las17-bindingproteinactinregulator	1	1
Breastcarcinomaamplifiedsequence2(BCAS2)	1	1
Hyaluronan/mRNAbindingfamily	1	3
DNLzincfinger	1	1
MitochondrialexportproteinSom1	1	1
RibosomalproteinL34e	1	1
MitochondrialimportreceptorsubunitTom22	1	1
YEATSfamily	1	1
CytochromeCoxidasecopperchaperone(COX17)	1	1
Spc19	1	1
RibosomalproteinS16	1	1
PLAC8family	1	1
Acetyltransferase(GNAT)domain	5	1

Domainofunknownfunction(DUF1993)	3	1
SWIMzincfinger	1	1
ARP2/3complexARPC3(21kDa)subunit	1	1
RibosomalproteinS19	3	7
Dihydroneopterinaldolase	1	1
alpha/betahydrolasefold	7	1
Conidiationprotein6	3	1
Nnf1	1	1
Rdxfamily	1	1
GGLdomain	1	9
UBA-likedomain	1	1
Mitochondrial39-SribosomalproteinL47(MRP-L47)	1	1
Yqey-likeprotein	1	3
RibosomalproteinL23	1	1
39SribosomalproteinL53/MRP-L53	1	1
Splicingfactor3Bsubunit10(SF3b10)	1	1
Ribosome-assemblyprotein3	1	1
CSN8/PSMD8/EIF3Kfamily	3	1
THUMPdomain	1	3
Erv1/Alrfamily	1	1
Proteinkinasedomain	11	1
Domainofunknownfunction(DUF1917)	1	1
Ubiquitincarboxyl-terminalhydrolase,family1	1	1
UncharacterisedACR,YggUfamilyCOG1872	1	1
CUEdomain	1	1
Ribosomalproteins50S-L18Ae/60S-L20/60S-L18A	1	5
HYPKUBAdomain	1	1
ATPsynthase	17	1
Pet100	1	3
UncharacterisedproteinfamilyUPF0047	1	3
Zinc-fingerdomain	1	5
RibosomalproteinL1p/L10efamily	3	1
G10protein	1	1
RNApolymeraseRpb3/Rpb11dimerisationdomain	3	1
CVNHdomain	3	1
ECL1/2/3zincbindingproteins	3	1
Glucose-repressibleproteinGrg1	5	1
4'-phosphopantetheinyltransferasesuperfamily	1	1
ConservedhypotheticalATPbindingprotein	1	3
RibosomalproteinL6e	1	1

RibosomalL29protein	1	1
HCNGP-likeprotein	1	1
Proteinofunknownfunction(DUF3712)	1	1
PHD-finger	1	1
PeptidaseinhibitorI78family	1	1
TranslationmachineryassociatedTMA7	1	1
DNAdirectedRNApolymerase,7kDasubunit	1	1
Coiled-coildomain-containingprotein56	1	1
Sds3-like	1	1
FungalZn(2)-Cys(6)binuclearclusterdomain	5	3
Exonuclease	1	1
Ankyrinrepeats(manycopies)	3	1
Vacuolarimportanddegradationprotein	1	1
RibosomalproteinS11	1	1
NADARdomain	1	1
LSMdomain	9	1
Phytanoyl-CoAdioxygenase(PhyH)	1	1
Oxidoreductase-likeprotein,N-terminal	1	1
Alternativeoxidase	1	1
Adenylatekinase	1	1
Adenylylsulphatekinase	1	1
Modifierofrudimentary(Mod(r))protein	1	1
Ubiquitin-2likeRad60SUMO-like	1	1
Complex1protein(LYRfamily)	7	1
Flavoprotein	3	1
SCP-2steroltransferfamily	1	3
CytochromecoxidasebiogenesisproteinCmc1like	3	5
RibosomalproteinL17	1	1
Mitochondrialimport2	1	1
Mitochondrialribosomalsubunitprotein	1	1
Tctex-1family	1	1
RibosomalL28family	1	1
Disorderedregionofunknownfunction(DUF5315)	1	9
RibosomalproteinS12/S23	1	1
Mo25-like	1	1
Bromodomain	5	1
ProteasomematurationfactorUMP1	1	1
Basicregionleucinezipper	7	7
FAM192A/Fyv6,N-terminaldomain	1	1
Glutaredoxin	1	1

Hsp20/alphacrystallinfamily	7	1
CentromereproteinH(CENP-H)	1	5
MitochondrialATPsynthaseBchainprecursor(ATP-synt_B)	1	1
CBF/Mak21family	1	1
CCAAT-bindingtranscriptionfactor(CBF-B/NF-YA)subunitB	1	1
DASHcomplexsubunitDad4	1	1
Profilin	1	1
CytochromecoxidaseassemblyproteinCOX16	1	3
Ribonucleotidereductaseinhibitor	1	1
Translationmachinery-associatedprotein16	1	1
Mitochondrialglycoprotein	1	5
Carbonicanhydrase	5	1
FADbindingdomain	5	1
Gammainterferoninduciblelysosomalthiolreductase(GILT)	1	1
HITdomain	1	1
TAP42-likefamily	1	1
DASHcomplexsubunitAsk1	1	1
5-formyltetrahydrofolatecyclo-ligasefamily	1	1
TranslationinitiationfactorIF-3,C-terminaldomain	1	1
Proteinofunknownfunction(DUF1115)	1	1
BCCIP	1	1
ATPsynthase(F/14-kDa)subunit	1	9
CytochromeoxidasecsubunitVIb	3	1
DNAreplicationandcheckpointprotein	1	11
Tyrosinephosphatasefamily	1	1
RibosomalproteinS13/S18	1	1
Trm112p-likeprotein	1	1
COG(conservedoligomericGolgi)complexcomponent,COG2	1	1
Vacuolarproteinsorting-associatedprotein62	1	1
RibosomalproteinL19	1	1
TranscriptionfactorTFIIHcomplexsubunitTfb5	1	1
RibosomalL22eproteinfamily	1	3
Anp1	1	1
Celldivisioncontrolprotein14,SINcomponent	1	1
Enoyl-CoAhydratase/isomerase	5	1
RibosomalproteinS8	3	1
MoaEprotein	1	1
Rix1complexcomponentinvolvedin60Sribosomematuration	1	1
Cellulase(glycosylhydrolasefamily5)	1	1
DNArepairproteinCrb2Tudordomain	1	3

Sen15protein	1	1
Yippeezinc-binding/DNA-binding/Mis18,centromereassembly	1	1
SCA7,zinc-bindingdomain	1	1
RibosomalL37aeproteinfamily	1	1
VanZlikefamily	1	1
BolA-likeprotein	1	1
RecQmediatedgenomeinstabilityprotein	1	1
Phosphopantetheineattachmentsite	1	1
Utp11protein	1	1
Pyridinenucleotide-disulphideoxidoreductase	1	1
Eukaryoticinitiationfactor4E	1	1
Rox3mediatorcomplexsubunit	1	3
HemerythrinHHEcationbindingdomain	5	1
RibosomalproteinS6	1	1
OHCUdecarboxylase	1	1
HITzincfinger	1	1
OsmC-likeprotein	1	1
Domainofunknownfunction(DUF5102)	1	1
MitochondrialATPaseinhibitor,IATP	1	1
MitochondrialribosomalproteinmL59	1	3
Proteinphosphataseinhibitor	1	3
Glu-tRNAGlnamidotransferaseCsubunit	1	1
Domainofunknownfunction(DUF3425)	7	3
TIP41-likefamily	1	11
PeptidaseM76family	1	5
RegulatorofGproteinsignalingdomain	1	1
helix-turn-helix,Psqdomain	11	106
Conservedmidregionofcactin	1	19
Zincfinger,ZZtype	1	1
Stress-inducedbacterialacidophilicrepeatmotif	5	1
DDEsuperfamilyendonuclease	41	1
Poly(ADP-ribose)polymerasecatalyticdomain	7	1
Extracellulartail,of10TMputativephosphatetransporter	3	1
TransposaseIS4	29	3
Ankyrinrepeats(3copies)	10	7
Zincknuckle	7	3
ribosomalL5PfamilyC-terminus	1	1
RNaseH	11	5
RibosomalproteinL35Ae	1	1
BCS1Nterminal	1	1

Nas2N_terminaldomain	1	3
Ubiquitinfamily	3	1
Proteinofunknownfunction(DUF3140)	1	1
N-terminaldomainofoxidoreductase	1	1
Subunit21ofMediatorcomplex	1	1
CRAL/TRIOdomain	1	1
(2R)-phospho-3-sulfolactatesynthase(ComA)	1	1
NACdomain	1	1
Pre-PUA-likedomain	1	3
Vesiculovirusphosphoprotein	1	9
MULEtransposasedomain	5	3
Phosphotransferaseenzymefamily	5	1
Aflatoxinregulatoryprotein	1	1
Metallo-beta-lactamasesuperfamily	1	1
CoAbindingdomain	1	1
NADbindingdomainof6-phosphogluconatedehydrogenase	1	1
HelicaseconservedC-terminaldomain	1	1
MCMAAA-liddomain	1	1
MOSCdomain	1	3
Annexin	1	1
Dioxygenase	11	1
CytochromeP450	19	1
Seedmaturationprotein	1	1
Zinc-bindingdehydrogenase	3	1
Cyclin,N-terminaldomain	5	1
Tetratricopeptiderepeat	3	1
SesBdomainonfungaldeath-pathwayprotein	1	7
Clathrinadaptorcomplexsmallchain	1	1
Zinc-bindingdomain	1	11
Cyclin-dependentkinaseregulatorysubunit	1	1
Cysteine-richTMmodulestresstolerance	1	1
Proteinofunknownfunction(DUF3759)	1	1
MycotoxinbiosynthesisproteinUstYa	1	9
Proteinofunknownfunction(DUF3445)	1	1
Oxysterol-bindingprotein	1	1
Ubiquinol-cytochromeCreductasecomplex14kDsubunit	1	1
Uncharacterizedproteinconservedinbacteria(DUF2263)	1	1
Carbon-nitrogenhydrolase	1	1
Haem-containingdehydratase	1	1
F-boxdomain	3	7

SnoaL-likedomain	7	1
FungalN-terminaldomainofSTANDproteins	1	5
galactosyltransferaseGMA12/MNN10family	3	1
Eukaryoticproteinofunknownfunction(DUF829)	1	1
KRdomain	1	1
Aminoacidpermease	1	1
DomainofUnknownFunction(DUF1907)	1	1
Fungalspecifictranscriptionfactordomain	5	1
Autophagocytosisassociatedprotein,active-sitedomain	1	1
NmrA-likefamily	26	1
ATPsynthasesubunitD	1	1
HMG(highmobilitygroup)box	1	5
CytochromecoxidasesubunitVIa	1	3
Orsellinicacid/F9775biosynthesisclusterproteinD	15	5
TranslationinitiationfactorSUI1	3	1
Fungalproteinofunknownfunction(DUF1752)	7	1
Bacterialproteinofunknownfunction(DUF924)	1	1
Asp/Glu/Hydantoinracemase	1	1
Polyketidecyclase/dehydraseandlipidtransport	3	7
Caspasedomain	3	7
Fungalfruitbodylectin	1	1
Antibioticbiosynthesismonooxygenase	3	1
Prion-inhibitionandpropagation	3	1
Thioesterasesuperfamily	3	1
YCII-relateddomain	1	3
Frequencyclockprotein	1	1
Ferritin-likedomain	1	1
Golgiphosphoprotein3(GPP34)	1	1
Ribose/GalactoseIsomerase	3	1
ADP-ribosylglycohydrolase	1	1
NACHTdomain	5	5
AAAdomain	1	1
RibosomalproteinS14p/S29e	3	1
GATAzincfinger	5	3
Glycosyltransferasefamily64domain	1	1
Heterokaryonincompatibilityprotein(HET)	5	1
Enoyl-(Acylcarrierprotein)reductase	5	7
Sulfotransferasedomain	3	1
Choline/ethanolaminekinase	1	1
2EXRfamily	3	1

2'-5'RNAligasesuperfamily	1	3
GINScomplexprotein	1	3
SETdomain	3	1
LITAF-likezincribbondomain	5	1
Domainofunknownfunction(DUF4385)	1	1
CysteinedioxygenasetypeI	1	5
Scytalonedehydratase	1	1
Cytochromec	17	4
Fic/DOCfamily	8	5
Carboxymuconolactonedecarboxylasefamily	3	1
RNaseH-likedomainfoundinreversetranscriptase	3	3
Glyoxalase/Bleomycinresistanceprotein/Dioxygenasesuperfamily	7	5
Amino-transferaseclassIV	1	1
GMCoxidoreductase	1	1
3-octaprenyl-4-hydroxybenzoatecarboxy-lyase	1	1
C2H2typezinc-finger(2copies)	5	1
SRR1	1	1
Chromo(CHRromatinOrganisationMOdifier)domain	7	1
gag-polypeptideofLTRcopia-type	1	1
NUDIXdomain	5	5
Putativeoxalocrotonatetautomeraseenzyme	1	1
Proteinofunknownfunction(DUF1479)	1	3
Phosphatidylserinedecarboxylase	1	3
Thiamine-bindingprotein	1	1
NAD(P)-bindingRossmann-likedomain	3	1
HNHendonuclease	9	5
T5orf172domain	5	3
Proteinofunknownfunction(DUF3295)	7	1
Conservedhypotheticalprotein(DUF2461)	1	1
FAM32A	1	5
Proteinofunknownfunction(DUF659)	1	3
Asparaginase,N-terminal	1	1
Wingedhelix-turnhelix	1	1
Adenosinedeaminase	1	1
Aldehydedehydrogenasefamily	1	13
WLMdomain	3	1
Transposase	33	1
Homeodomain	1	3
Putativeserineesterase(DUF676)	1	1
Copperamineoxidase,N2domain	1	1

Patatin-likephospholipase	1	1
O-methyltransferasedomain	1	1
Alpha-acetolactatedecarboxylase	1	1
Reversetranscriptase(RNA-dependentDNApolymerase)	15	1
HSF-typeDNA-binding	1	1
Helitronhelicase-likedomainatN-terminus	9	1
Pleckstrinhomologydomain	1	1
FAR1DNA-bindingdomain	1	1
HpcH/HpaIaldolase/citratelyasefamily	1	1
Uncharacterizedalpha/betahydrolasedomain(DUF2235)	1	1
non-haemdioxygenaseinmorphinesynthesisN-terminal	1	1
Beta-1,3-glucanase	1	3
Responseregulatorreceiverdomain	1	1
Endonuclease-reversetranscriptase	1	1
FMN-dependentdehydrogenase	1	1
14-3-3protein	1	1
Nuclearcap-bindingproteinsubunit3	1	3
ARP2/3complex16kDasubunit(p16-Arc)	1	1
Proteinofunknownfunction(DUF3128)	1	3
RibosomalL15	1	1
Proteinofunknownfunction(DUF2462)	1	1
Fcf2pre-rRNAprocessing	1	1
KinetochorecomplexFta4ofSim4subunit,orCENP-50	1	3
KinetochoreSim4complexsubunitFTA2	3	1
RibosomalproteinL37e	1	1
CytochromecoxidaseassemblyproteinPET191	1	1
Iron-sulphurclusterbiosynthesis	1	3
ChromatinassociatedproteinKTI12	1	1
CENP-SassociatingCentromereproteinX	1	1
Macrophagemigrationinhibitoryfactor(MIF)	1	1
RF-1domain	1	1
Frataxin-likedomain	1	1
KAPfamilyP-loopdomain	1	1
Rpp14/Pop5family	1	1
SnoaL-likepolyketidecyclase	3	1
HistonechaperonedomainCHZ	1	1
MitochondrialK+-H+exchange-related	1	1
Phosphatidylinositol-4-phosphate5-Kinase	1	1
MitochondrialimportproteinPam17	1	5
Zincfinger,C3HC4type(RINGfinger)	1	1

DehydroquinaseclassII	1	1
MitochondrialF1-F0ATPsynthasesubunitFoffungi	1	1
RibosomalproteinS19e	1	1
BAGdomain	1	1
Ubiquinol-cytochromeCreductasehingeprotein	1	1
Proteinofunknownfunction(DUF2457)	1	1
Putativeesterase	1	1
Apc13pprotein	1	3
Uncharacterisedprotein(DUF2406)	1	1
RibosomalproteinL36e	1	1
Dualspecificityphosphatase,catalyticdomain	1	1
Sybindin-likefamily	1	1
Clr5domain	3	1
Proteinofunknownfunction(DUF1674)	1	1
D-Tyr-tRNA(Tyr)deacylase	1	1
RHOproteinGDPdissociationinhibitor	1	1
Bacterialtransferasehexapeptide(sixrepeats)	1	1
Haloaciddehalogenase-likehydrolase	3	1
RanBP1domain	1	1
MIOREXcomplexcomponent7	1	1
RibosomalproteinS26e	1	1
TubulinbindingcofactorA	1	1
EisosomecomponentPIL1	1	1
Pam16	1	1
Cenp-Okinetochorecentromerecomponent	1	1
EMG1/NEP1methyltransferase	1	1
AAA-ATPaseVps4-associatedprotein1	1	1
ArgininosuccinatelyaseC-terminal	1	1
NB-ARCdomain	1	1
Acylphosphatase	1	1
Transcriptionalregulator	1	1
AutophagyreceptorATG43	1	1
ChaC-likeprotein	1	1
Proteinofunknownfunction(DUF1223)	1	1
ATPsynthaseEchain	1	1
MitochondrialribosomalproteinL27	1	1
Mitochondrialpyruvatecarriers	1	1
Proteinofunknownfunction(DUF1687)	1	1
RibosomalproteinL14	5	1
ATPsynthaseDchain,mitochondrial(ATP5H)	1	1

SOH1	1	1
Thaumatinfamily	1	3
NADdependentepimerase/dehydratasefamily	1	1
Phosphoglucomutase/phosphomannomutase,alpha/beta/ alphadomainI	1	1
Maltoseacetyltransferase	1	1
ADP-ribosylationfactorfamily	1	1
Transcriptionalactivatorofglycolyticenzymes	1	1
N-terminaldomainonNACHT_NTPaseandP-loopNTPases	1	1
DADfamily	1	1
Alteredinheritanceofmitochondria5	1	1
CoproporphyrinogenIIIoxidase	1	1
LYAR-typeC2HCzincfinger	1	1
Domainofunknownfunction(DUF202)	1	1
LETM1-likeprotein	1	1
AlcoholdehydrogenaseGroES-likedomain	1	1
3-hydroxyanthranilicaciddioxygenase	1	1
Dpy-30motif	1	1
Alb1	1	1
NADHubiquinoneoxidoreductasesubunitNDUFA12	1	1
Fructose-bisphosphatealdolaseclass-II	1	1
Isy1-likesplicingfamily	1	1
KinetochoreproteinMis14like	1	1
Ringfingerdomain	3	0
Bacteriocin-protection,YdeIorOmpD-Associated	1	0
RibosomalproteinS21	3	0
CHYzincfinger	1	0
Proteinofunknownfunction(DUF3723)	1	0
UbiquinonebiosynthesisproteinCOQ7	1	0
Pirin	1	0
Catalase	1	0
Bucentaurorcraniofacialdevelopment	1	0
BUD22	1	0
ThreonylandAlanyltRNAsynthetasesecondadditionaldomain	1	0
50Sribosome-bindingGTPase	1	0
39SmitochondrialribosomalproteinL46	1	0
4Fe-4Sdiclusterdomain	1	0
MybDNA-bindinglike	1	0
Mtf2family	1	0
Amidase	1	0
TatDrelatedDNase	1	0

GCN5-likeprotein1(GCN5L1)	1	0
Ctf8	1	0
DASHcomplexsubunitDad3	1	0
CorehistoneH2A/H2B/H3/H4	1	0
EutQ-likecupindomain	1	0
WGRdomain	3	0

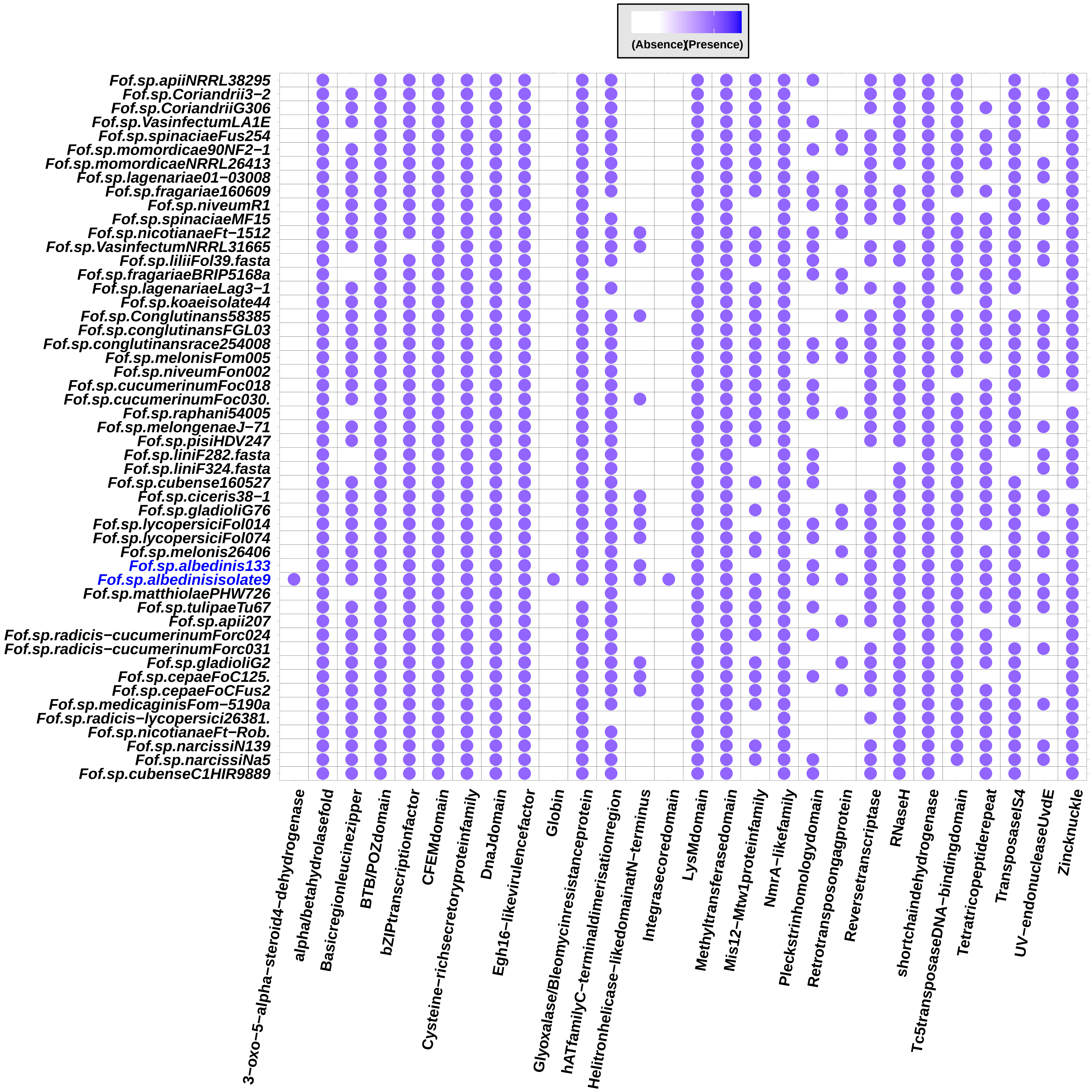

Figure 1 : Presence/absence map of Foa unique effector distribution in the effectore repertoire of Foa and other analyzed formae speciales. The bubbles indicate the presence of Foa unque effectors and the empty boxes represent their absence in each strain. Abbreviations: (Fusarium); o (oxysporum); f (forma); sp (specialis). The names following the abbreviations represent the names of the formae speciales; the abbreviations coming after the names of the formae speciales correspond to the strains belonging to these formae speciales (F.o.f.sp. albedinis 133: Fusarium osysporum forma specialis albedinis Foa 133).
