# Supplementary material for: In silico comparative genomic analysis unravels a new candidate protein arsenal specifically associated with Fusarium oxysporum f. sp. albedinis pathogenesis
Source: Sci Rep. 2022 Nov 9;12:19098. doi: 10.1038/s41598-022-21858-1 (PMC9646873; doi:10.1038/s41598-022-21858-1)
Supplement: Supplementary file 4 — Supplementary Information 4. [file 41598_2022_21858_MOESM4_ESM.pdf]

**Supplementary data 4 : Table 1 : predicted secreted CAZYme families /sub-families of *Fusarium oxysporum*.f. sp. *albedinis* strains**					
***Fusarium oxysporum*. f. sp. *albedinis* 133**			***Fusarium oxysporum*. f. sp. *albedinis* 9**		
**CAZYme classes**	**CAZYme families/sub-families**	**CAZYme count**	**CAZYme classes**	**CAZYme families/sub-families**	**CAZYme count**
AA	AA1_3	5	AA	AA1_3	4
AA	AA2	3	AA	AA3_2	3
AA	AA3_2	3	AA	AA3_3	1
AA	AA3_3	1	AA	AA5_2	5
AA	AA5_2	5	AA	AA6	1
CBM	CBM1	7	AA	AA9	1
CBM	CBM18	2	CBM	CBM1	8
CBM	CBM20	1	CBM	CBM18	2
CBM	CBM32	4	CBM	CBM32	4
CBM	CBM38	1	CBM	CBM38	1
CBM	CBM42	1	CBM	CBM42	1
CE	CE1	2	CE	CE1	2
CE	CE4	1	CE	CE4	2
CE	CE5	4	CE	CE5	4
CE	CE8	1	CE	CE8	1
CE	CE12	1	CE	CE12	1
CE	CE16	1	CE	CE16	1
GH	GH3	16	GH	GH2	4
GH	GH5_5	2	GH	GH3	18
GH	GH5_7	1	GH	GH5_5	1
GH	GH5_9	1	GH	GH5_7	1

GH	GH5_15	1	GH	GH5_15	2
GH	GH6	1	GH	GH6	1
GH	GH7	2	GH	GH7	2
GH	GH10	3	GH	GH10	3
GH	GH11	3	GH	GH11	3
GH	GH12	2	GH	GH12	2
GH	GH13_1	1	GH	GH13_1	1
GH	GH15	1	GH	GH13_25	1
GH	GH17	3	GH	GH13_40	1
GH	GH18	3	GH	GH16_1	1
GH	GH27	1	GH	GH17	3
GH	GH28	8	GH	GH18	4
GH	GH31	2	GH	GH27	1
GH	GH32	1	GH	GH28	8
GH	GH35	3	GH	GH31	2
GH	GH36	1	GH	GH32	1
GH	GH37	1	GH	GH35	3
GH	GH43_5	2	GH	GH36	1
GH	GH47	2	GH	GH37	1
GH	GH49	1	GH	GH38	1
GH	GH53	1	GH	GH43_1	1
GH	GH54	1	GH	GH43_5	2
GH	GH55	1	GH	GH43_11	1
GH	GH62	1	GH	GH47	5
GH	GH63	1	GH	GH49	1
GH	GH65	1	GH	GH51	1
GH	GH67	2	GH	GH53	1
GH	GH75	2	GH	GH54	1

GH	GH76	2	GH	GH55	1
GT	GT2	4	GH	GH62	1
GT	GT4	2	GH	GH63	1
GT	GT31	3	GH	GH65	1
GT	GT32	1	GH	GH67	2
GT	GT33	1	GH	GH75	2
GT	GT39	3	GH	GH76	2
GT	GT57	1	GH	GH133	1
GT	GT58	1	GT	GT1	3
GT	GT59	1	GT	GT2	3
PL	PL1_2	1	GT	GT4	3
PL	PL1_4	5	GT	GT32	1
PL	PL1_7	3	GT	GT33	1
PL	PL3_2	6	GT	GT39	2
PL	PL4_1	1	GT	GT48	1
PL	PL4_3	2	GT	GT57	1
			GT	GT66	1
			PL	PL1_2	1
			PL	PL1_4	5
			PL	PL1_7	3
			PL	PL3_2	6
			PL	PL4_1	1
			PL	PL4_3	2
